# A Liver-Centric Multiscale Modeling Framework for Xenobiotics

**DOI:** 10.1371/journal.pone.0162428

**Published:** 2016-09-16

**Authors:** James P. Sluka, Xiao Fu, Maciej Swat, Julio M. Belmonte, Alin Cosmanescu, Sherry G. Clendenon, John F. Wambaugh, James A. Glazier

**Affiliations:** 1 Biocomplexity Institute Indiana University Bloomington, Bloomington, IN 47405-7105, United States of America; 2 National Center for Computational Toxicology Office of Research and Development US EPA, Research Triangle Park, NC 27711, United States of America; Montana State University Bozeman, UNITED STATES

## Abstract

We describe a multi-scale, liver-centric in silico modeling framework for acetaminophen pharmacology and metabolism. We focus on a computational model to characterize whole body uptake and clearance, liver transport and phase I and phase II metabolism. We do this by incorporating sub-models that span three scales; Physiologically Based Pharmacokinetic (PBPK) modeling of acetaminophen uptake and distribution at the whole body level, cell and blood flow modeling at the tissue/organ level and metabolism at the sub-cellular level. We have used standard modeling modalities at each of the three scales. In particular, we have used the Systems Biology Markup Language (SBML) to create both the whole-body and sub-cellular scales. Our modeling approach allows us to run the individual sub-models separately and allows us to easily exchange models at a particular scale without the need to extensively rework the sub-models at other scales. In addition, the use of SBML greatly facilitates the inclusion of biological annotations directly in the model code. The model was calibrated using human *in vivo* data for acetaminophen and its sulfate and glucuronate metabolites. We then carried out extensive parameter sensitivity studies including the pairwise interaction of parameters. We also simulated population variation of exposure and sensitivity to acetaminophen. Our modeling framework can be extended to the prediction of liver toxicity following acetaminophen overdose, or used as a general purpose pharmacokinetic model for xenobiotics.

## Introduction

Pharmacological and toxicological processes occur across a wide range of spatial and temporal scales and include multiple organ systems. An in silico pharmacological model must include sub models that cover these multiple scales and multiple tissues relevant to human medicine and toxicology. We have developed a liver-centered, mechanism based, multiscale in silico simulation framework for xenobiotic toxicity and metabolism that incorporates four key biological scales: (1) Population variation scale, (2) Physiologically Based Pharmacokinetic (PBPK) whole body scale, (3) Tissue and multicellular scale, and (4) Sub-cellular signaling and metabolic pathways scale. Our multiscale in silico framework focuses on the liver, a critical organ in many toxicological, pharmacological, normal and disease processes. As an example of linking these models, from the whole body to the sub-cellular, we present a model of acetaminophen (*APAP*, paracetamol in most of the world) Absorption, Distribution, Metabolism and Excretion (ADME). We link existing open source tools into an aggregate model instead of building a single monolithic model. This approach allows us to leverage not only pre-existing tools but also pre-existing models at the individual scales.

The liver is the primary metabolizing organ of the body and is the first line of defense against toxicants. The liver is a “massively parallel device” that presents unique challenges and opportunities for modeling not present in other organs. The liver’s central role in metabolism places it at the center of many pharmacological and toxicological processes. In addition, the liver is a key player in both the body’s homeostatic control processes and in a number of important human diseases such as hypercholesterolemia, obesity and Type II diabetes. Our choice of APAP as the reference compound was based on the extensive literature on its pharmacology and toxicology, including extensive data in both humans and laboratory animals. APAP is a widely used over the counter analgesic and fever reducer. APAP’s therapeutic index (ratio of toxic dose to therapeutic dose) is unusually small for an over the counter medication. Overdose results in rapid centrilobular necrosis (cell death in the vicinity of the smallest draining veins) of the liver, which can lead to liver failure and death. APAP is the leading cause of acute liver failure in the Western world [1].

The liver is a critical organ with essential functions in uptake, storage and metabolism of amino acids, lipids, carbohydrates, and vitamins [2]. The liver also plays a central role in metabolism and elimination of xenobiotics. Its anatomical position in the blood flow between the gastro-intestinal (GI) tract and systematic circulation allows the liver to act as a gateway linking the GI system with the rest of the body [3]. The liver is composed of millions of functional units, termed hepatic lobules, that are “plumbed” by the liver vasculature in a highly parallel layout. When a portion of blood (e.g., a red blood cell) transits the liver it passes through exactly one lobule. Blood enters the lobule via portal triads, consisting of an arteriole, venule and bile duct, and after passing through the sinusoid network drains into the lobule’s central vein. Each hepatic lobule consists of several cell types. Hepatocytes, the parenchymal cell of the liver, constitute the majority of liver’s mass and carry out the majority of the metabolism of nutrients and toxins. Non-parenchymal cells, including Kupffer cells, liver stellate (Ito) cells and sinusoidal endothelial cells, make up the remainder of the liver’s mass and also contribute to the liver’s functions. Kupffer cells are resident macrophages of the liver that contribute to normal physiology and homeostasis as well as participating in immune responses to liver injury [4, 5]. Stellate cells contribute to flow sensing and regulatory functions and become activated in response to liver damage [6] (see [7] for a comprehensive review). Fenestrated (porous) endothelial cells form the linings of the capillary vessel walls, the liver sinusoids, and dynamically regulate vessel size and blood flow in response to mechanical and chemical signals. The endothelial cell lined sinusoids are separated from the hepatocytes by the perisinusoidal space (Space of Disse) that contains blood plasma and microvilli from the hepatocytes, which facilitates rapid transfer of compounds between the blood and the parenchyma of the lobule. In addition to the sinusoids the lobule contains a second flow path, the bile canaliculi, that transport bile from the hepatocytes back to the portal triad where it is collected into the bile ducts.

Each lobule displays heterogeneous capabilities both in terms of blood flow and hepatocyte activity. In each lobule, hepatocytes radiate from the center to the periphery separated by a network of sinusoids. Blood flow enters the hepatic lobule via the hepatic artery and hepatic portal vein, flows through the network of sinusoids past the hepatocytes, and empties into the lobule’s central vein. Hepatocytes situated in different regions of the hepatic lobule have different expression levels of transporter proteins and different metabolic enzymes profiles resulting in regional differences in metabolism across the lobule and sinusoid [8]. In addition, the highly interconnected network of sinusoids results in complex blood flow within each lobule [9].

APAP is extensively metabolized in the liver via both Phase I and Phase II pathways [10]. Phase II pathways convert APAP into sulfate and glucuronide conjugates. Phase I metabolism, primarily via Cytochrome P450s (*CYP*) 2E1 and 1A2, generates N-acetyl-p-benzoquinone imine (*NAPQI*), an alkylating agent capable of covalently modifying a number of cellular components. The majority of NAPQI reacts with cellular glutathione (*GSH*) giving a thiol adduct NAPQI-GSH. In cases of APAP overdose, depletion of cellular GSH is thought to be causative in hepatocyte death (necrosis). Acute overdose of APAP results in extensive necrosis of hepatocytes in the centrilobular regions of the liver [11]. Although it is not fully understood how the depletion of GSH progresses to necrosis, it’s believed that reactive oxygen species play a significant role [12]. The combination of Phase I and II metabolism is a common feature of xenobiotic metabolism and applies, to varying degrees, to most xenobiotics.

**Fig 1:**
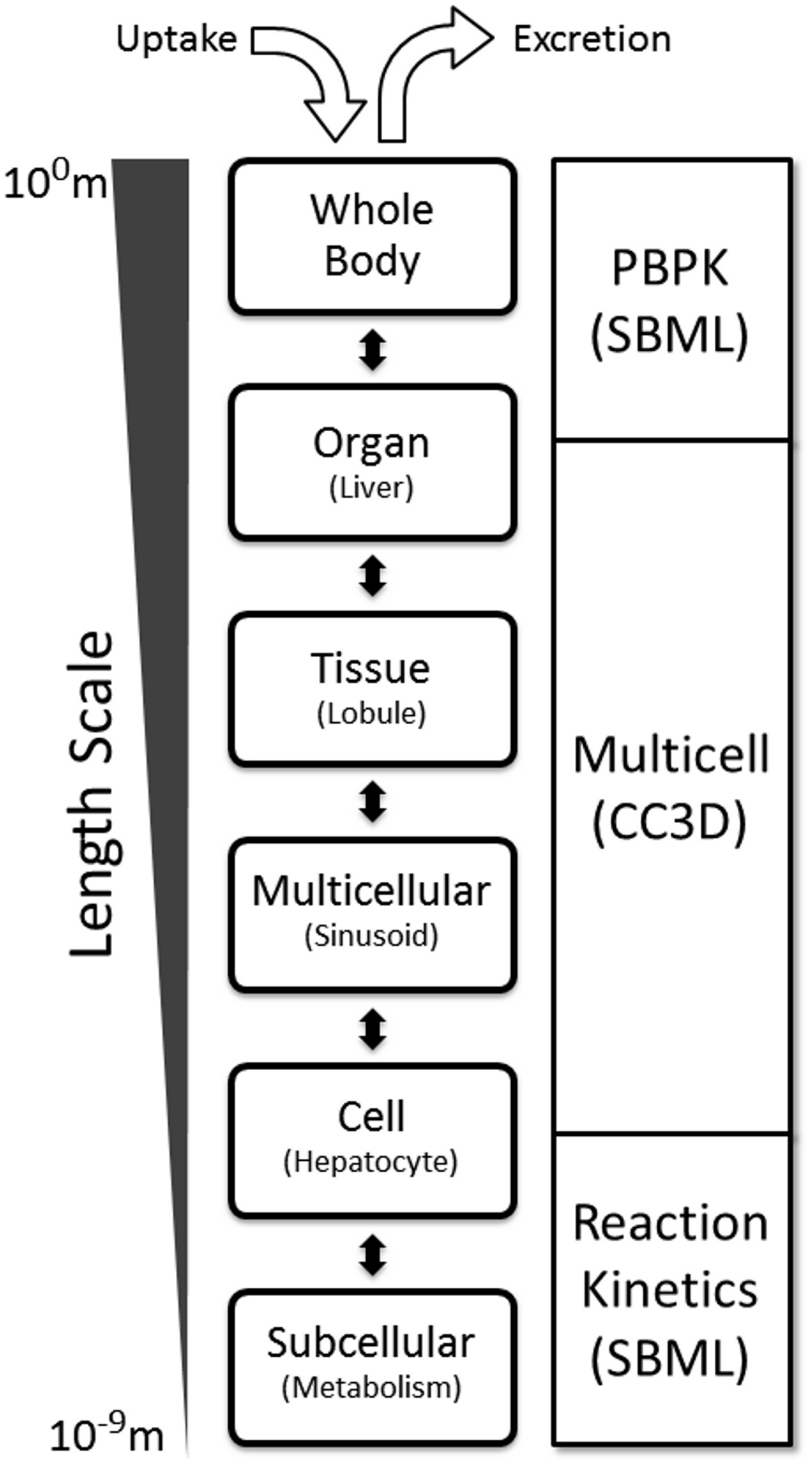
Biological scales and modeling modalities. Length scales (left) of biological processes (center) and the corresponding modeling modalities (right).

**Computational models of liver function and toxicity:** Modeling a toxicological or pharmacological event requires the integration of processes that spread across different spatial and temporal scales as shown in Fig 1. At the whole body level, compounds are absorbed from the GI tract, distributed among the tissues and organs, and filtered and excreted by the kidneys. The compound diffuses within the blood, and partitions between the serum, serum proteins (such as albumin) and blood cells. At the liver tissue level, compounds enter the liver via the hepatic artery and portal vein, flow through the network of sinusoids within the hepatic lobules, and empties into the hepatic central vein. The compound transports, generally via both passive (diffusive) and active transport pathways, into and out of the hepatocytes lining the sinusoids. At the sub-cellular level, metabolic pathways within the hepatocytes convert the compound into metabolites. The metabolites are transported (either actively or passively) back into the blood circulation or into the bile pathway. Hepatocytes may proliferate, migrate or die, in response to the compound or its metabolites. Gene expression patterns orchestrate the spatial distribution of the metabolic processes within the lobule. To computationally model the multiple scales as well as their interactions we must integrate temporally and spatially across the relevant tissues.

Many studies have used whole-body PBPK models to describe APAP ADME [13–19]. These models are able to reproduce the ADME data for APAP and its metabolites and are validate against *in vivo* data. Some of these models elaborate the description of APAP metabolism and toxicity through integration of a more detailed subcellular genome-scale flux balance metabolism model [14] or kinetic model of GSH depletion [15, 18]. Other studies focused on systems biology models of APAP metabolism and GSH homeostasis, such as [20]. Ben-Shachar *et. al* [19] developed an extended PBPK model of APAP metabolism in humans. Their model includes transport and metabolism of APAP, to the glucuronide and sulfate metabolites, as well as GSH metabolism. This model was calibrated using *in vitro* human data for APAP and the two major metabolites and models the toxicity of APAP as well as the effectiveness of clinical interventions for APAP overdoses.

One important aspect missing from these models is the spatial architecture of the metabolizing organ and the spatial distribution of metabolic enzymes such as CYP450 2E1 at different lobular locations. A model of APAP metabolism that treats the liver as a “well stirred” compartment may miss or underestimate the heterogeneity of the liver’s responses. With advancement of imaging techniques, recent modeling studies increasingly incorporate organ-specific structural and spatial features, in addition to biochemical properties of endogenous or exogenous compounds, into their representation of the liver and in understanding metabolism and toxicity [21–23]. Perhaps the most complete attempt at reconstructing the complex microvasculature of a liver lobule was accomplished by Hoehme *et. al* [21]. To our knowledge, this reconstructed lobule has not been used to model blood flow within the lobule, or microdosimetry of individual hepatocytes. The reconstructed lobule has also been combined with a whole-body PBPK model and a two or three compartment ODE based lobule model, giving a multiscale model that couples from the whole-body to liver-lobule zone [22, 23].

Additional models of the complex blood flow within a liver lobule have been created using advection-diffusion models in which the liver lobule is treated as a homogeneous, though perhaps anisotropic, medium [24]. These models make predictions on qualities such as the blood flow rate and pressure drop across a lobule but do not model discrete sinusoids or hepatocytes. Additional advection-diffusion models have been developed that combine the lobule scale with a whole body scale [25] and that include cellular signaling and metabolic pathways [26]. These later models have been applied to a selection of compounds, such as caffeine and insulin, but have not been applied to APAP. Several of these projects are part of the German “Virtual Liver Network” (*VLN*) (http://www.virtual-liver.de), which has made progress in constructing multiscale, liver-centric modeling frameworks (*e*.*g*. [26–28]). The VLN project is attempting to incorporate submodels that span the entire range of biological scales.

Hunt and coworkers developed a physiologically based object-oriented in silico liver (ISL) model that characterizes hepatic disposition of compounds via geometrical representations of liver structure and function. This model included a blood flow network at the lobular level, small molecule transport and metabolism within individual simulated hepatocytes at the cellular level, all embedded in a whole-body PBPK model [29, 30]. The model was calibrated using human *in vitro* data and functional responses of ISL agents were mapped back to the *in vitro* data.

Wambaugh and Shah constructed a spatially heterogeneous sinusoidal network representing a hepatic lobule, and used the virtual lobule as a replacement for the liver compartment in a whole-body PBPK model. They then used this aggregate model to calculate tissue microdosimetry [31].

Diaz Ochoa and coworkers developed a multiscale model of the toxic effects of APAP [32]. In their model, the liver lobule was represented as a set of unbranched sinusoids and surrounding hepatocytes. Individual hepatocytes contained instances of the subcellular metabolic pathways. This liver lobule was then embedded in a whole-body PBPK. Movement of blood born APAP within the sinusoids was modeled using an advection-diffusion equation.

Most of the models described above suffer from an important limitation; it is difficult to reproduce or reuse the models since the source codes were not published. Even when source code is published it is often difficult to map biological concepts (e.g., chemical species, biological processes, etc.) in the publication to variables in the source code. To effectively make a model reusable requires that the model is encoded in a standard syntax. The standard syntax should be able to describe both the computation and the biology that the model represents.

**Scientific Goals:** We set out to develop a first attempt at a general purpose xenobiotic modeling framework capable of integrating three key biological scales in a shareable and well documented way. The scales range form the whole-body to the sub-cellular. Integration across these scales allows for the coupling of experimental data obtained at a variety of scales, ranging from ADME data describing uptake, distribution and clearance, to tissue and cell level microdosimetry in a key organ (liver), to subcellular reaction kinetics models describing metabolism. The framework is meant to be shareable and reusable, in whole or part, by other workers and in other biological modeling applications. Finally, the framework is meant to demonstrate annotation of the included biological concepts within the model files to the extent currently possible.

**Design Goals for a Multiscale—Multimodal Model:** We set out to build a multi-scale model of xenobiotic metabolism that conforms to the following design principles:

1. Combine multiple spatial and temporal scales to create a single composite model.
2. Use standard modeling formats for each scale where possible.
3. Annotate the model at each scale to the extent possible.
4. Reuse existing models for each scale where possible.
5. Minimize the effort required to modify (or replace) the sub-model at a particular scale without needing to extensively rework the entire model.

Reuse of existing models, and adherence to modeling standards at the particular scales, is a key aspect of the Responsible Conduct of Research. (http://www.nsf.gov/bfa/dias/policy/rcr.jsp)

**Fig 2:**
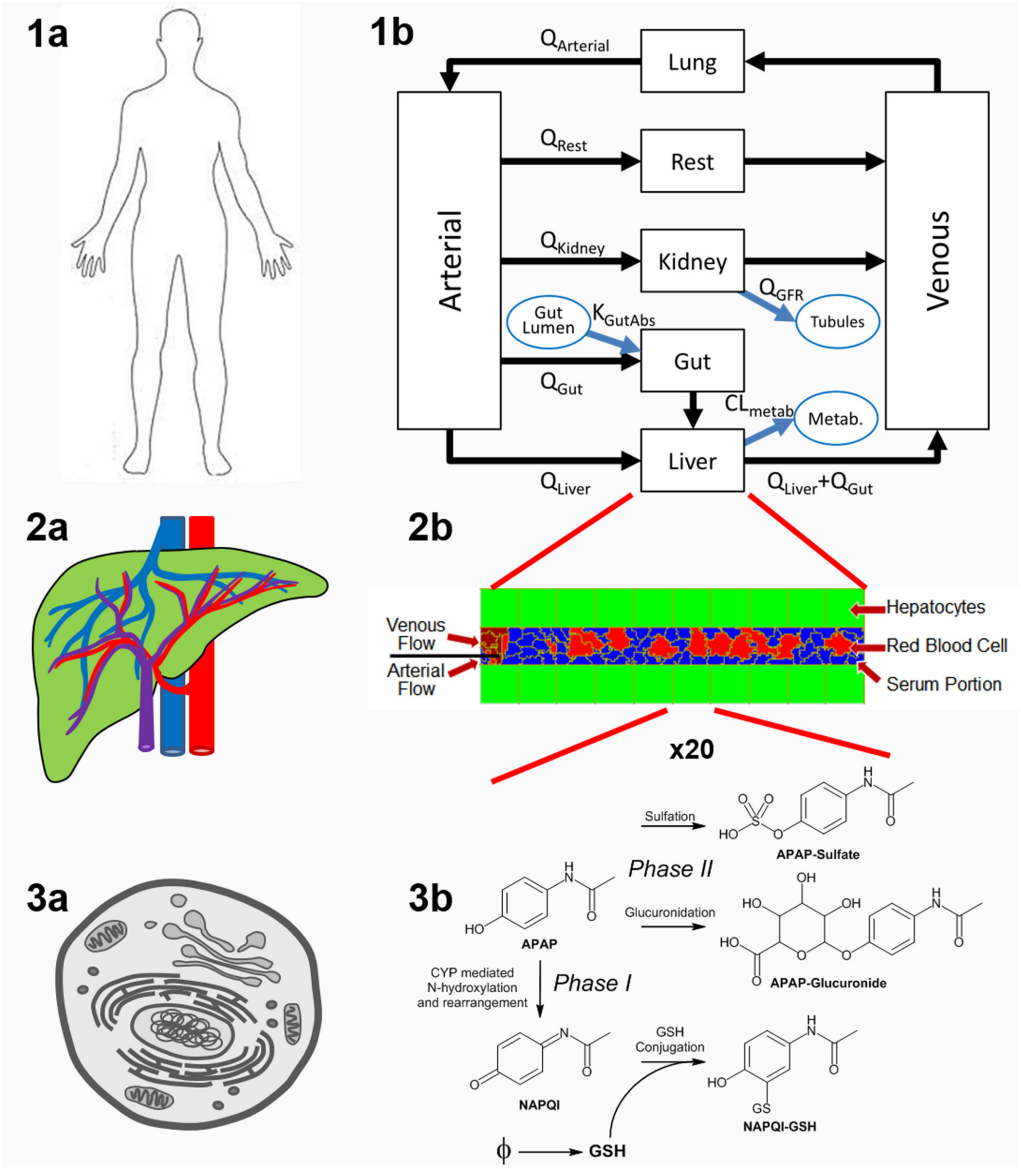
Description of modular structure of the model. Whole body uptake, distribution and clearance (1a) is represented as a PBPK model (1b) in SBML. The rectangular compartments represent tissues or blood compartments with blood flows between compartments indicated by black arrows. The compartment labeled “Rest” is the remainder of the body. The blue arrows and ovals represent non-blood borne transfer between compartments and organ lumens or, in the case of liver metabolism, an unspecified location external to the PBPK model. The tissue scale model of the liver (2a) is represented in CC3D as a single sinusoid modeled as a linear pipe lined with hepatocytes (green) (2b). Blood flow within the sinusoid consists of RBCs (red) and portions of serum (blue). Portions of serum are modeled as generalized cells. The hepatocyte (3a) metabolic pathways (3b) are model as a set of chemical reactions expressed in SBML.

**Model framework:** We introduce a multiscale modeling framework for small molecule, liver-centric, multiscale ADME modeling that bridges different temporal and spatial scales (Fig 1) and is built and executed using standard open-source software packages. At the whole-body level, we take advantage of PBPK modeling to characterize the disposition (*i*.*e*., absorption, distribution, metabolism and excretion) of APAP (Fig 2:1b). The PBPK model allows us to couple the tissue and lower scale models to the common routes of whole-body uptake and clearance, which is of critical importance [27]. In addition, the majority of *in vivo* data for xenobiotics in humans consists of xenobiotic concentrations measured in the blood or serum, which is well modeled by PBPK. At the organ and tissue levels, we construct a virtual liver based on parallel tube simplification and simulate blood flow within a sinusoid using the Cellular Potts Model [33, 34] (Fig 2:2b). At the sub-cellular level, we describe GSH synthesis and APAP metabolism (both Phase I and Phase II) within each hepatocyte using reaction kinetic models (Fig 2:3b). The composite model is flexible and adaptable and can be modified to represent different species, populations or xenobiotics. Each module in our framework is sufficiently complete to run in the absence of the other modules, which makes it easier to explore different modules and computational modalities at the individual scales. Our framework is a step towards developing defensible, annotated, shareable and reusable models of small molecule ADME and liver based metabolism and toxicity.

As a proof of concept drug for our multiscale framework we choose APAP. APAP is rapidly absorbed from GI tract following an oral dose, distributed across organs via the systemic circulation, metabolized by the liver and other tissues, and excreted by the kidneys. In humans, there are three primary metabolic pathways for APAP (Fig 2:3b). At therapeutic doses the majority of APAP is metabolized by the liver to form glucuronide and sulfate conjugates (APAPG and APAPS), both are non-toxic, non-therapeutic and rapidly cleared. 55% to 60% of the dose recovered in the urine is present as APAPG, and APAPS accounts for 30% to 35% [35, 36]. Only a small amount of unchanged APAP is detected in the urine. In the liver APAP also undergoes Phase I metabolism, mediated by CYP enzymes, to form the reactive moiety N-acetyl-p-benzoquinone imine (NAPQI). For pharmacological doses of APAP, NAPQI is effectively detoxified through conjugation with cellular Glutathione (GSH). However, high doses of APAP lead to overproduction of NAPQI and consequently depletion of hepatic GSH, which leads to necrosis.

In this work, we model a pharmacological doses of APAP and leave the modeling of APAP overdose and hepatocyte necrosis for future study. A simulated therapeutic dose of 20 mg/Kg orally administered to a 70 Kg (1.4g total dose) in silico male patient is used as the standard model. We are particularly interested in predicting the serum concentrations (ADME curves) of APAP, APAPG and APAPS for the 8 hours following the single oral dose.

## Models

Our workflow for developing a computational model includes three types of model definitions. The first model definition is of a *Biological Model* (also known as a conceptual model) that is typically created with the help of a biological domain expert. This model is often graphical in nature (for example Fig 2:1b, 2b and 3b) and describes what is known about the system and what is thought to be relevant to a particular problem. This level of modeling detail is familiar to biologists and typically includes a list of the parts and their interactions.

The second model definition is a of *Mathematical Model* where the components and processes of the biological model are expressed in mathematical forms that implement the physical and chemical interpretations of the biological components (as in Table 1). At this stage of model development rigorous definitions of the mathematical forms, the parameters and measurables of the model are defined. Here the modeler is forced to make significant assumptions about the behavior of the system and these assumptions challenge and add value to the starting biological model. The mathematical model is the point where the modeling workflow migrates from the biological domain to the physics, chemistry or engineering domains.

**Table 1:** Whole-body PBPK Model Rate Equations.

Transfer	Rate equation
*AGutlumen* → *CGut*	*kGutabs* ⋅ *AGutlumen*
*CGut* → *CLiver*	*QGut* ⋅ *CGut*/*VGut*
*CLiver* → *CVen*	(*QLiver* + *QGut*) ⋅ *CLiver* ⋅ *Rb*2*p*/(*Kl*2*p* ⋅ *Fup* ⋅ *VLiver*)
*CLiver* → *CMetabolized*	*CLmetab*. ⋅ *CLiver*/(*Kl*2*p* ⋅ *Fup*)
*CVen* → *CLung*	*QCardiac* ⋅ *CVen*/*VVen*
*CLung* → *CArt*	*QCardiac* ⋅ *CLung*/*VLung*
*CArt* → *CGut*	*QGut* ⋅ *CArt*/*VArt*
*CArt* → *CLiver*	*QLiver* ⋅ *CArt*/*VArt*
*CArt* → *CKidney*	*QKidney* ⋅ *CArt*/*VArt*
*CArt* → *CRest*	*QRest* ⋅ *CArt*/*VArt*
*CKidney* → *CVen*	*QKidney* ⋅ *CKidney* ⋅ *Rb*2*p*/(*Kk*2*p* ⋅ *Fup* ⋅ *VKidney*)
*CKidney* → *CTubules*	*Qgfr* ⋅ *CKidney*/(*Kk*2*p* ⋅ *VKidney*)
*CRest* → *CVen*	*QRest* ⋅ *CRest* ⋅ *Rb*2*p*/(*Kr*2*p* ⋅ *Fup* ⋅ *VRest*)

Model based on [31]. *A* = variable mass (*moles*) of compound in a volume-less compartment, *C* = variable mass (*moles*) of compound in a compartment, *Q* = volumetric flow rate (*L*/*sec*), *V* = compartment perfusable volume (*L*), *K* = compound partition coefficient (unitless), *F*_*up*_ = fraction unbound to protein (unitless), *R* = ratio (unitless). *A* and *C* are variables, the rest of the values are constants (parameters). The aggregated rate equations are available in the PBPK SBML file and in Table 3 of the S1 File.

The third level of model definition is the conversion of the mathematical model into a *Computational Model* where the components of the mathematical model are implemented in computational code. There may be implementation-dependent uncertainties introduced at this step if the computational representation does not adequately represent the mathematical model. Therefore, there is a verification step needed to insure that the computational model adequately represents the mathematical model. If the computational model uses an excepted compute platform then some of the verification effort can be avoided. For example, for a mathematical model consisting of a set of coupled ODEs the modeler could (1) create a solver from scratch, or they could (2) use a preexisting ODE solver package (e.g., MATLAB’s *dsolve* package), or if the ODEs represent a set of chemical reactions, they could (3) use a standard modeling platform like COPASI [37] (www.copasi.org) or CellDesigner [38] (www.celldesigner.org) specifically designed for that type of problem. In the first case it is difficult for a third party to verify the code since they would need access to both the code and to the computing environment. In addition, the linkage between biological concepts and the code may be obscure. In the second case third-party verification is somewhat easier since the reliability of the ODE solver package may have already been confirmed by other workers. Verification is also easier in the third case. The third case also has an important advantage over the first two in that it is often possible to *annotate* the source code by identifying the biological concepts that particular computational constructs, such as variables and subroutines, represent. It is only in the third case where it is practical to directly link the computational model back to the biological model.

For each of the three biological scales that we represent there are biological, mathematical and computational models, which we will describe in detail below.

### Biological models

A multi-scale in silico model is expected to characterize processes taking place at each scale, and to integrate the processes and scales in an appropriate manner. In our modeling framework, three different biological scales are taken into account.

1. The whole-body PBPK module describes the absorption, distribution and clearance of APAP. APAP is absorbed from GI tract, distributed throughout the body via the systematic circulation, and excreted by the kidneys. The liver receives both arterial and venous blood [9](Fig 2:1b). The same core PBPK model is also used to model the distribution and clearance of the APAP metabolites (APAPG and APAPS).
2. At the tissue scale we model a single hepatic sinusoid (Fig 2:2b). At the periportal end of the sinusoid compound-rich blood enters the sinusoid via both the hepatic artery and portal vein flows and flows past a row of non-motile hepatocytes. Blood is pushed from the periportal end to the central vein end of the sinusoid. The blood consists of red blood cells (RBCs, 40-45% of the total blood volume) and plasma. Small molecules are carried by the blood in one of three possible states: 1) free in solution (serum), (2) bound to serum proteins (e.g., albumin) or (3) bound to, or contained in, RBCs. Within the blood the compound can transfer between the three blood states (free, protein bound, and RBC bound). Only xenobiotic free in the serum is capable of being transported into the hepatocytes and that transfer occurs at the sinusoid-hepatocyte interface. Transfer between the blood and hepatocytes consists of both passive (diffusive) transfer and active transport. The active transport may be either inwardly or outwardly rectified. Transfer between the blood and hepatocytes is constant along the entire length of the sinusoid.
3. Each hepatocyte is equipped with its own copy of the pathways for Phase I and Phase II metabolism. Phase II metabolism includes glucuronidation and sulfation pathways. Both Phase II metabolites are nontoxic and are rapidly exported out of the hepatocytes into the blood. Phase I metabolism is also included and for APAP this pathway generates NAPQI. Phase I metabolites are assumed to be too reactive to escape the cell in which they were made. NAPQI reacts with the relatively high hepatocyte GSH concentration (10mM) [39, 40] forming NAPQI-GSH, which also does not exit the cell. Total NAPQI-GSH produced within a given cell is a marker of the total stress on the cell caused by the toxic metabolite [12]. Cells produce GSH at a slow rate and the total cellular concentration never exceeds 10mM. The rate of Phase I metabolism within the hepatocytes increases linearly from the periportal to centrilobular ends of the sinusoid to approximate the zonal distribution of the CYPs.

In addition to the three scales described above we can also simulate a population as a set of model replicates, each with individualized parameters.

### Mathematical and Computational models

We have used standard modeling modalities for each of the three scales introduced above. Subcellular reaction kinetics are modeled as a set of coupled ordinary differential equations (ODEs) using the Systems Biology Markup Language (*SBML*) [41, 42], which was specifically developed for reaction kinetic models such as this. The multicell sinusoid tissue model was modeled in two dimensions using Compucell3D (CC3D), an implementation of the Cellular Potts model. CC3D implements the flowing blood (RBCs and serum) within the sinusoid along with the fixed hepatocytes. CC3D also implements the diffusive and active transport processes between the components of the blood and the hepatocytes. For the whole-body PBPK scale there is currently no accepted standard modeling modality. Since PBPK models are typically sets of coupled ODEs we have used SBML to model this scale as well. We use the SBML“compartment” concept to represent the PBPK tissue compartments.

A key advantage of developing models in SBML is the ability to directly annotate *biological components within the model*. For example, in SBML a chemical species can be annotated by reference to the *Chemical Entities of Biological Interest* ontology (https://www.ebi.ac.uk/chebi/), compartments by reference to cell (e.g., http://obofoundry.org/ontology/cl.html) or tissue (http://sig.biostr.washington.edu/projects/fm/) ontologies and reactions and biological processes to, for example, the *Gene Ontology* (http://geneontology.org/).

**Whole-body PBPK Module:** We use a standard PBPK compartment model approach to model the uptake, distribution and clearance of APAP following oral dosing (Fig 2:1b). In this model, boxes represent the quantity of APAP in an organ or blood compartment. Compartments are modeled as “well stirred” so that no concentration gradients exist within a compartment. Arrows represent the movement of drug-bearing blood between different compartments of the body. Typical parameters in PBPK models include the volumetric flow rates between compartments (*e*.*g*., organs), compartment perfusable volumes, partition coefficients, unbound fraction of xenobiotic in plasma and the hematocrit (RBC to blood volume ratio). The equations for the transfer rates are given in Table 1 and the base set of parameters are given in Table 2. The parameters for a typical PBPK model can be divided into two subsets: parameters that are independent of the compound being modeled, such as tissue volumes and blood flow rates, and parameters that are compound dependent, such as partition coefficients and uptake rates. These two types of parameters are listed separately in Table 2. Since there is no accepted standard for the specification of PBPK models we have used SBML as the specification language for the PBPK ODEs. The PBPK model of Wambaugh and Shah [31] was translated into SBML using SBW tools, particularly Jarnac [43] and JDesigner [44]. (The Systems Biology Workbench, including Jarnac, is available at http://sys-bio.org/.) Annotation was carried out using SBMLeditor [45] (http://www.ebi.ac.uk/compneur-srv/SBMLeditor.html) and the resulting SBML validated, including the units [46] (http://sbml.org/Facilities/Validator/). We paid particular attention to annotating the compounds (e.g., APAP and metabolites) and compartments (organs), and ensured that all parameters had SBML compliant unit definitions. Compartment volumes and blood flow rates used standard PBPK values [47] that were allometrically scaled to a 70 Kg human male. In this PBPK model the metabolism of APAP in the liver is represented by a single ODE representing removal (metabolism) of APAP. APAP is known to be extensively reabsorbed from the kidney tubules [36], in our model we represent this by modifying the glomerular filtration rate (Qgfr).

**Table 2:** PBPK Parameter Set REFSIM.

Parameter	Value	Definition (Source)
*Compound Independent Parameters*		
*pbpk*_*bw*	70*Kg*	Body weight (assumed)
*Compound Independent Parameters, allometrically scaled by Body Weight*		
*pbpk*_*QCardiac*	363.01*L*/*h*	Blood flow rate of cardiac output [47]
*pbpk*_*QGut*	74.42*L*/*h*	Blood flow rate of gut compartment [47]
*pbpk*_*QLiver*	19.42*L*/*h*	Blood flow rate of liver compartment [47]
*pbpk*_*QKidney*	80.37*L*/*h*	Blood flow rate of kidney compartment [47]
*pbpk*_*QRest*	188.80*L*/*h*	Blood flow rate of the rest of the body [47]
*pbpk*_*VArt*	1.50*L*	Volume of Arterial compartment (estimated based on [47, 48])
*pbpk*_*VVen*	3.41*L*	Volume of Venous compartment (estimated based on [47, 48])
*pbpk*_*VGut*	1.10*L*	Volume of Gut compartment (estimated based on [48])
*pbpk*_*VLiver*	1.71*L*	Volume of Liver compartment (estimated based on [48])
*pbpk*_*VKidney*	0.29*L*	Volume of Kidney compartment (estimated based on [48])
*pbpk*_*VLung*	0.51*L*	Volume of Lung compartment (estimated based on [47, 48])
*pbpk*_*VRest*	33.47*L*	Volume of the rest of the body (estimated)
*Compound Dependent Parameters*		
*pbpk*_*kGutabs*	1.5*h*^−1^	Gut absorption rate of APAP [16, 32, 49]
*pbpk*_*Fup*	0.8	Unbound fraction of APAP in plasma [50]
*pbpk*_*FupG*	1	Unbound fraction of APAPG in plasma (assumed)
*pbpk*_*FupS*	1	Unbound fraction of APAPS in plasma (assumed)
*pbpk*_*Kr*2*p*	1.6	Rest of body-to-Plasma partition coefficient of APAP (estimated from [16, 32])
*pbpk*_*Kr*2*pG*	0.4	Rest of body-to-Plasma partition coefficient of APAPG (estimated)
*pbpk*_*Kr*2*pS*	0.2	Rest of body-to-Plasma partition coefficient of APAPS (estimated)
*pbpk*_*Kk*2*p*	1	Kidney-to-Plasma partition coefficient of APAP (estimated from [16, 32])
*pbpk*_*Kk*2*pG*	1	Kidney-to-Plasma partition coefficient of APAPG (assumed)
*pbpk*_*Kk*2*pS*	1	Kidney-to-Plasma partition coefficient of APAPS (assumed)
*pbpk*_*Kl*2*p*	1	Liver-to-Plasma partition coefficient of APAP (estimated from [16, 32]) but overridden by the multiscale model
*pbpk*_*Kl*2*pG*	1	Liver-to-Plasma partition coefficient of APAPG (assumed) but overridden by the multiscale model
*pbpk*_*Kl*2*pS*	1	Liver-to-Plasma partition coefficient of APAPS (assumed) but overridden by the multiscale model
*pbpk*_*Rb*2*p*	1.09	Blood-to-Plasma ratio of APAP [51, 52]
*pbpk*_*Rb*2*pG*	0.55	Blood-to-Plasma ratio of APAPG (assumed no binding to RBCs)
*pbpk*_*Rb*2*pS*	0.55	Blood-to-Plasma ratio of APAPS (assumed no binding to RBCs)
*pbpk*_*Qgfr*	0.714*L*/*h*	Glomerular filtration rate of APAP [47]
*pbpk*_*QgfrG*	7.86*L*/*h*	Glomerular filtration rate of APAPG [47]
*pbpk*_*QgfrS*	9.96*L*/*h*	Glomerular filtration rate of APAPS [47]

Replicates of this APAP PBPK model, with differing compound specific parameters and initial conditions, was used to model the distribution and clearance of the two APAP phase II metabolites, APAPG and APAPS, in the complete multiscale model. This whole-body PBPK model has been deposited in the BioModels Database [63] (http://www.ebi.ac.uk/biomodels-main/) as MODEL1509230010.

**Tissue Module:** The tissue module describes the behavior of blood flow within a simplified hepatic sinusoid as well as the transport of xenobiotic between the blood components and hepatocytes. We used the open-source multicell modeling package CompuCell3D (CC3D) [53] to model the blood components and flow, the hepatocytes and transfer of APAP (and metabolites) between the blood and hepatocytes. CC3D, an implementation of the Cellular Potts Model [54], is a lattice based description of systems of generalized biological cells where each individual cell (or portion of an amorphous region like extracellular matrix or bone) is treated as a collection of pixels (2D) or voxels (3D). Each cell is characterized by an energy function designed to reproduce typical qualities and behaviors of cells including volume and surface area, cell-cell adhesion, chemotaxis etc. CC3D does not attempt to minimize the “energy” of the system but instead simulates a dynamics trajectory where the system evolves over time by passing through energetically “feasible” configurations. The feasibility of a particular state depends on the deviations from the target qualities (volume, surface area, …) of the cells. A Boltzmann function and a factor (roughly analogous to a temperature) controls the probability of visiting higher energy states. CC3D has been used to model many cell scale biological phenomena such as embryonic development [55], tumor growth with angiogenesis [56] and the retina [57].

Here we model the structure of a liver lobule as a simple sinusoid tube connecting the portal triad at the periportal region to the central vein at the centrilobular region with hepatocytes lining the sinusoidal pipe (Fig 2-2b). We do not explicitly model the endothelial cells lining the sinusoid nor the space of Disse. This simple 2-dimensional model is taken to be representative of all liver sinusoids. Blood, including red blood cells (RBCs) and serum portions, are modeled within this sinusoidal pipe. Blood flow simulated using CC3D includes the two components (RBCs and serum portions) and both components are created at the periportal end through division of pseudo-cells of type “blood source cell”. A constant force is exerted on both blood components to induce blood flow through the simulated sinusoid. When a blood component reaches the central vein end of the simulated sinusoid they are deleted from the model. The temporal scale was adjusted so that blood speed in the simulation is equivalent to 200 *μ*m/s, giving a transit time of a blood component through the sinusoid of one second.

**Fig 3:**
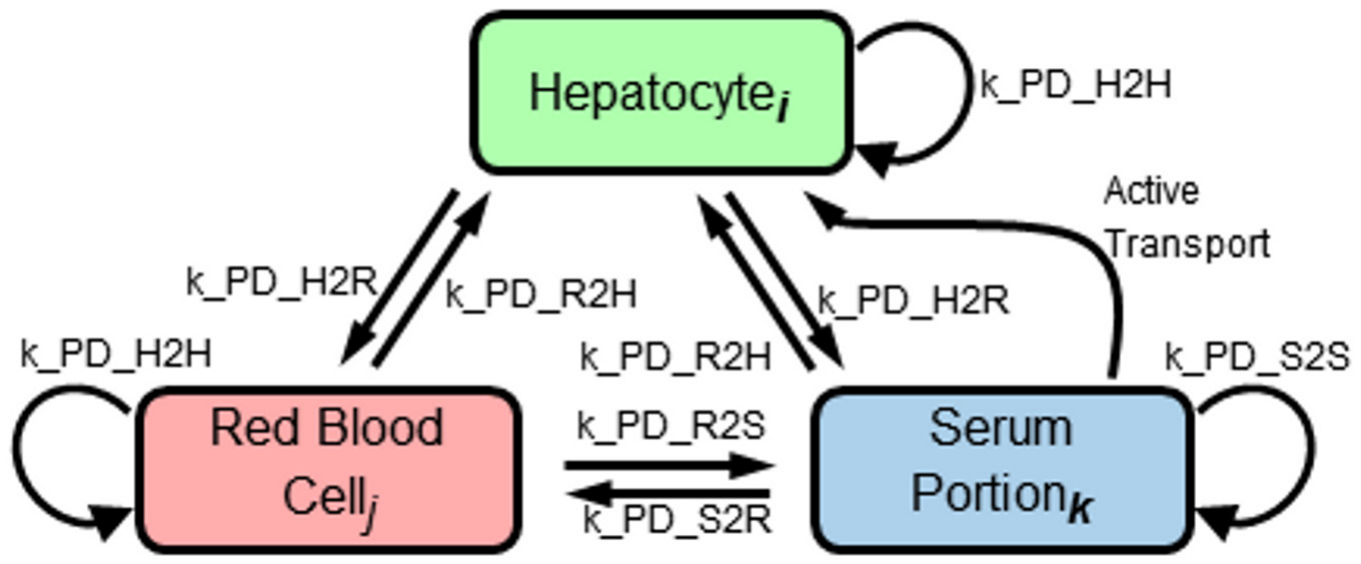
The diffusion model for the multicell scale sinusoid model. Transfer between each of the three cell, or pseudo-cell, *types* is described by this transfer map. Subscripts indicate that there are multiple cells of each of the types and transfer is calculated between all pairs of cells that are in contact at a particular instant. The paired arrows represent passive transport that equilibrates across pairs of cell types if they are in contact. Looped arrows represent passive transport between adjacent cells of the same type. The single arrow labeled “Active Transport” represents the Michaelis-Menten modeled import of APAP from the serum into hepatocytes.

APAP, carried by RBCs and plasma, enters this system from the periportal end and is forced to move towards the pericentral end. During transit, drug molecules diffuse between adjacent serum portions, RBCs and hepatocytes. These transfers are modeled as reversible first order mass action transfers and the transfer flux is scaled by the number of neighbors (RBCs, serum portions or hepatocytes)(Fig 3). In addition, inwardly rectified active transport between serum and hepatocytes is modeled as a separate Michaelis-Menten (saturable) process. The transfer rate constants are given in Table 3 and the parameters in Table 4. This tissue model is a mix of “well stirred” and explicit gradients; individual cells or serum portions are treated as “well stirred”, but transfer between individual cells and/or serum portions is explicitly modeled giving concentration gradients across the simulated sinusoid.

**Table 3:** Intercellular Molecule Transfer Rate Equations.

Transfer^†^	APAP^‡^	APAPG, APAPS
$C_{H}^{i}\to C_{H}^{j}$	$cc3d\_k\_PD\_H2H\cdot C_{H}^{i}/n^{i}$	
$C_{H}^{i}\to C_{R}^{j}$	$cc3d\_k\_PD\_H2R\cdot C_{H}^{i}/n^{i}$	$cc3d\_k\_AT\_APAPG\cdot C_{H}^{i}$, $cc3d\_k\_AT\_APAPS\cdot C_{H}^{i}$
$C_{R}^{i}\to C_{R}^{j}$	$cc3d\_k\_PD\_R2R\cdot C_{R}^{i}/n^{i}$	
$C_{S}^{i}\to C_{H}^{j}$	$cc3d\_k\_PD\_S2H\cdot C_{S}^{i}/n^{i}+\frac{cc3d\_Vmax\_AT\_APAP\cdot C_{S}^{i}}{C_{S}^{i}\;+\;cc3d\_Km\_AT\_APAP}\cdot pbpk\_Fup$	
$C_{H}^{i}\to C_{S}^{j}$	$cc3d\_k\_PD\_H2S\cdot C_{H}^{i}/n^{i}$	$cc3d\_k\_AT\_APAPG\cdot C_{H}^{i}$, $cc3d\_k\_AT\_APAPS\cdot C_{H}^{i}$
$C_{S}^{i}\to C_{R}^{j}$	$cc3d\_k\_PD\_S2R\cdot C_{S}^{i}/n^{i}$	
$C_{R}^{i}\to C_{S}^{j}$	$cc3d\_k\_PD\_R2S\cdot C_{R}^{i}/n^{i}$	
$C_{S}^{i}\to C_{S}^{j}$	$cc3d\_k\_PD\_S2S\cdot C_{S}^{i}/n^{i}$	

^†^Superscripts refer to the unique identifiers for each cell. The set of cells *C*^*j*^ are the contact neighbors of the cell *C*^*i*^. Subscripts identify the cell type: *H* = hepatocyte, *S* = serum portion, *R* = red blood cell

^‡^ *pbpk*_*Fup* = Fraction unbound to protein, *n*^*i*^ = number of contact neighbors.

The rate equations are available in Tables 4–6 of the S1 File.

**Table 4:** CC3D Parameters.

Parameter	Value	Definition	Source
*Compound Independent Parameters*			
*mcs*2*sec*	1/600 *sec*/*mcs*	Time conversion ratio; simulated time(second) = *mcs*2*sec* * Monte Carlo step	assumed
*px*2*um*	1 *μm*/*pixel*	Space conversion ratio; simulated distance (micrometer) = *px*2*μm* * visualized pixels	assumed
*Vol*_*HEP*_	20 * 20 * 20 *μm*^3^	Volume of hepatocyte	estimated
*l*_*SIN*_	200 *μm*	Length of sinusoid	estimated
*d*_*SIN*_	20 *μm*	Width of sinusoid (2D width)	estimated
*h*_*SIN*_	4 *μm*	Thickness of sinusoid (Assumed thickness/depth; 4 is used in transfer calculation)	assumed
*Vol*_*SIN*_	20 * 200 * 4 *μm*^3^	Volume of sinusoid lumen, effective diameter of sinusoid = $\sqrt{\frac{20*4}{\pi }}$	calculated
*Vol*_*RBC*_	100 * 4 *μm*^3^	Target volume of RBC	
*Vol*_*SP*_	25 * 4 *μm*^3^	Target volume of serum portion	assumed
*Vol*_*BSC*_	50 * 4 *μm*^3^	Target volume of blood source cell	assumed
*NeighborOrder*	2 *pixel*	Neighbor order	assumed
*freq*_*bloodInflow*_	20 *mcs*	Diffusion calculation frequency	assumed
*freq*_*transport*_	10 *mcs*	Diffusion calculation frequency	assumed
*freq*_*SBML*_*subcell*__	10 *mcs*	Subcellular SBML update frequency	assumed
*freq*_*SBML*_*pbpk*__	10 *mcs*	PBPK SBML update frequency	assumed
*J*_*H*,*H*_	5	Adhesion energy between hepatocytes	estimated
*J*_*R*,*R*_	10	Adhesion energy between RBCs	estimated
*J*_*S*,*S*_	5	Adhesion energy between serum portions	estimated
*J*_*B*,*B*_	5	Adhesion energy between blood source cells	estimated
*J*_*H*,*R*_	10	Adhesion energy between hepatocyte and RBC	estimated
*J*_*H*,*S*_	5	Adhesion energy between hepatocyte and serum portion	estimated
*J*_*H*,*B*_	5	Adhesion energy between hepatocyte and blood source cell	estimated
*J*_*R*,*S*_	5	Adhesion energy between RBC and serum portion	estimated
*J*_*R*,*B*_	40	Adhesion energy between RBC and blood source cell	estimated
*J*_*S*,*B*_	40	Adhesion energy between serum portion and blood source cell	estimated
*Compound Dependent Parameters*			
*cc*3*d*_*k*_*AT*_*APAPG*	0.00045*s*^−1^	Transport rate of APAPG from hepatocyte into sinusoid	[32]
*cc*3*d*_*k*_*AT*_*APAPS*	0.0019*s*^−1^	Transport rate of APAPS from hepatocyte into sinusoid	[32]
*cc*3*d*_*Km*_*AT*_*APAP*	0.01*mmol*/*L*	APAP concentration to reach half-maximum transport rate	estimated from [32]
*cc*3*d*_*Vmax*_*AT*_*APAP*	0.01*mmol*/*L*/*s*	Maximum transport rate of APAP from sinusoid into hepatocyte	estimated from [32]
*cc*3*d*_*k*_*PD*_*R*2*R*	0.001*s*^−1^	APAP diffusion rate between red blood cells	estimated from [32]
*cc*3*d*_*k*_*PD*_*R*2*S*	0.001*s*^−1^	APAP diffusion rate from red blood cells to serum portion	estimated from [32]
*cc*3*d*_*k*_*PD*_*R*2*H*	0.001*s*^−1^	APAP diffusion rate from red blood cells to hepatocyte	estimated from [32]
*cc*3*d*_*k*_*PD*_*S*2*R*	0.0012*s*^−1^	APAP diffusion rate from serum portion to red blood cells	estimated from [32]
*cc*3*d*_*k*_*PD*_*S*2*S*	0.010*s*^−1^	APAP diffusion rate between serum portion	estimated from [32]
*cc*3*d*_*k*_*PD*_*S*2*H*	0.001*s*^−1^	APAP diffusion rate from serum portion to hepatocyte	estimated from [32]
*cc*3*d*_*k*_*PD*_*H*2*R*	0.001*s*^−1^	APAP diffusion rate from hepatocyte to red blood cells	estimated from [32]
*cc*3*d*_*k*_*PD*_*H*2*S*	0.001*s*^−1^	APAP diffusion rate from hepatocyte to serum portion	estimated from [32]
*cc*3*d*_*k*_*PD*_*H*2*H*	0.001*s*^−1^	APAP diffusion rate between hepatocytes	estimated from [32]

During a simulation the hepatocytes are fixed in space and do not move. The CC3D model creates serum portions and RBCs representing venous and arterial flow at the periportal end of the sinusoid. Scaling of blood flow from the PBPK module to the sinusoid model uses the volumetric blood flow and liver volume from the PBPK module, the fraction of the liver volume that is sinusoid lumen, 7.4% [21], and the volume of the simulated sinusoid. The ratio of RBC to serum portions is based on the hematocrit value (typically 0.4–0.45 in humans), which is also used by the whole-body PBPK model. The ratio of arterial versus venous flow is taken as 1:3 [9]. When an RBC or serum portion is created it is “loaded” with an amount of APAP based on the concentration of APAP in the inflow streams (from the whole-body PBPK model) taking into account both the venous and arterial inflow concentrations. As the blood moves through the sinusoid the diffusion into and out of the blood is calculated as described above. As the blood reaches the pericentral end of the sinusoid the remaining load of APAP is collected into an integrator and the RBC or serum portion is deleted. The integrated load is then transferred back to the whole-body PBPK model as the output from the liver compartment.

**Subcellular Module:** Phase I and Phase II metabolism of APAP within individual hepatocytes (Fig 2-3b) is modeled using Michaelis-Menten kinetics and the ODEs are given in Table 5 and the parameters are given in Table 6. In addition, glutathione (GSH) synthesis and NAPQI-GSH formation are included in the subcellular module. Each hepatocyte is treated as a “well stirred” container. The subcell reaction network was defined in SBML and, as was the case for the whole-body PBPK model, the species, reactants and units were extensively annotated and the final SBML validated. Each simulated hepatocyte has an independent copy of the subcellular reaction model. This SBML model has been deposited in the BioModels Database as MODEL1509230011.

**Table 5:** Subcellular Reaction Kinetics Rate Equations.

Reaction	Rate Equation
*APAP* → *NAPQI*	*sc*_*Vmax*_2*E*1 ⋅ [*APAP*]/(*sc*_*Km*_2*E*1 + [*APAP*])
*NAPQI* + *GSH* → *NAPQI* − *GSH*	*sc*_*k*_*NAPQIGSH* ⋅ [*NAPQI*] ⋅ [*GSH*]
*APAP* → *APAPG*	*sc*_*Vmax*_*GLUC* ⋅ [*APAP*]/(*sc*_*Km*_*GLUC* + [*APAP*])
*APAP* → *APAPS*	*sc*_*Vmax*_*SULF* ⋅ [*APAP*]/(*sc*_*Km*_*SULF* + [*APAP*])
*ϕ* → *GSH*	*sc*_*kGSH* ⋅ ([*GSH*_*max*_] − [*GSH*])

The rate equations are available in the subcellular reaction kinetics SBML file and in Table 7 of the S1 File.

**Table 6:** Subcellular Reaction Kinetic Parameters.

Parameter	Value	Definition	Source
sc_Vmax_GLUC	0.001*mmol*/*L*/*s*	Maximum reaction rate	estimated based on [58]
sc_Km_GLUC	1*mmol*/*L*	Concentration to reach half-maximum reaction rate	estimated based on [58]
sc_Vmax_SULF	0.000175*mmol*/*L*/*s*	Maximum reaction rate	estimated based on [58]
sc_Km_SULF	0.2*mmol*/*L*	Concentration to reach half-maximum reaction rate	estimated based on [58]
sc_Vmax_2E1_APAP	0.00002*mmol*/*L*/*s*	Maximum reaction rate	[58]
sc_Km_2E1_APAP	1.29*mmol*/*L*	Concentration to reach half-maximum reaction rate	[58]
sc_kNAPQIGSH	0.1*mmol*/*L*/*s*	Reaction rate for NAPQI-GSH production	estimated
sc_kGsh	0.0001*mmol*/*L*/*s*	Synthesis rate of GSH	estimated
*GSH*_0_	9.9*mmol*/*L*	Initial cellular GSH concentration	[59]
*GSH*_*max*_	10*mmol*/*L*	Maximum cellular GSH concentration	[59]

**Module Integration:** The complete multi-scale model uses Python CC3D code as the “marshaling point”. CC3D runs the multi-cellular model (including blood flow and APAP transfer between components), time steps the sub-cellular SBML and whole body PBPK/SBML models, and transfers quantities between scales. We choose the blood concentration of APAP as a scale-insensitive variable to connect the models. We use the LibRoadRunner [60] package, a cross-platform, open-source, high performance C++ library for running SBML-compliant models. LibRoadRunner’s Python API allows the CC3D Python script to load the SBML models (including independent replicates of the same model coupled to each simulated hepatocyte), adjust parameters, time step the SBML models and set and retrieve variables. We use independent replicates of the base PBPK SBML model for APAP, APAPG and APAPS, each with compound specific parameters. Replicates of the subcellular metabolic SBML model are linked to each individual hepatocyte in the tissue model. In total, the complete model includes of 23 linked SBML models; 20 copies of the sub-cellular reaction kinetic model (one for each of the simulated hepatocytes) and three copies of the whole body PBPK/SBML model for APAP, APAPG and APAPS.

Zonation of the CYPs is imposed in the CC3D model by scaling the *V*_*max*_ for CYP metabolism as a function of the hepatocyte’s location along the sinusoid axis; values increase along the periportal—pericentral axis from 80% to 100% of the sc_Vmax_2E1_APAP value (Table 6).

When CC3D loads an SBML model any process(es) in the SBML model that are not needed in the context of the complete multiscale model are disabled. For example, the whole-body PBPK model for APAP includes a single ODE representing liver metabolism that is not needed in the complete model since that process is represented by the subcellular model. To disable that particular ODE the rate constant in the SBML model is simply set to zero by the CC3D script. In addition to disabling selected ODEs, the CC3D script can overwrite initial conditions within the imported SBML files. For example, replicates of the APAP-PBPK model are used to model the APAPG and APAPS distribution so the initial concentration of APAP in the gut lumen compartment of those replicate models is set to zero.

In a simulation step, the CC3D script sequences both the calculations it carries out directly as well as time stepping the linked SBML models. Computationally, we integrate and update each of the three modules sequentially with the same time step. A single computational time step cycle consists of:

1. Time step the PBPK models updating the quantities of APAP, APAPG and APAPS in each compartment of the PBPK module.
2. Fetch the quantities of APAP within “CArt” and “CGut” compartments of the PBPK model and, using the PBPK model’s flow rates and hematocrit values, calculate the concentration of APAP entering the inlet side of the tissue-level (CC3D) sinusoid module.
3. The CC3D model creates blood portions and RBCs representing venous and arterial flow that contain the amount of APAP (or APAPG or APAPS) based on the blood concentrations in the two blood flows in the whole-body PBPK models.
4. The CC3D model then simulates blood flow for a short period of time (we used a flow time step of 16.7milliseconds).
5. We then numerically integrate the transfer of drug molecules at the interface of blood (serum portions and RBCs) and hepatocytes.
6. The CC3D script then time steps the subcellular SBML models within each hepatocyte, which generates new values for the per-cell concentrations of APAP and the metabolites.
7. At the central vein terminus of the sinusoid the APAP (and metabolites) are returned to the exit point of the “CVen” compartment of the PBPK module.

### Model Outputs

The complete multiscale model makes predictions of the time evolution of a number of concentrations. These range from subcellular concentrations of APAP and metabolites, as well as GSH, in each of the individual hepatocytes to blood concentrations by compartment for APAP and the metabolites. To directly compare model outputs with available *in vivo* data we focus on APAP, APAPG and APAPS in the serum. The time course of these species can be described by typical ADME descriptors including the time to maximum serum concentration (*Tmax*), the maximum serum concentration (*Cmax*) and the area under the curve (*AUC*) for all three compounds of interest in the blood; APAP, APAPG and APAPS. To quantify the goodness of fit for the entire time course data set we use the Root-Mean-Square Error (*RMSE*_*i*_) defined as:

$$RMSE_{i}=\sqrt{\frac{\sum_{j}^{N}{\left(y_{j}^{sim}-y_{j}^{exp}\right)}^{2}}{N}}$$ (1)

Where *y* is the model output of interest, *N* is number of data points in the *in vivo* ADME dataset; *i* refers to APAP, APAPG or APAPS giving RMSEa, RMSEg and RMSEs, respectively. In addition, we calculate the sum of the three RMSEs giving RMSEsum. An additional model output is the excreted metabolic ratio (*metabRatio*) defined as:

$$metabRatio=\frac{\left[APAPG]+[APAPS\right]}{\left[APAP]+[APAPG]+[APAPS\right]}$$ (2)

Where the concentrations are those in the kidney tubules (“Tubules” compartment in Fig 2:1b).

### Model Parameterization

Initial values of the parameters (Tables 2, 4 and 6) in our simulations are based on previous studies [31, 32] or, if no previous studies included a particular parameter value then it was estimated. In this study, we specifically focus on fitting the serum level of APAP and its Phase II metabolites to *in* *vivo* experiments [58]. Our target is to minimize the summation RMSEsum, the sum of the errors for the three ADME curves in our simulation. Multiple preliminary simulations were run with small changes in the parameter set and the set that best reproduced the *in vivo* ADME for a 1.4g APAP dose in humans, including APAP, APAPG and APAPS, was selected as the starting parameter set (see Results). For further details see S1 File.

### Sensitivity Analysis

Because of the large number of parameters in the complete model, as well as the use of different modeling modalities at the various scales, we examined the sensitivities of the model outputs to the parameters. This examination can help to identify parameters that the model is most sensitive to, and therefore, need to be known as accurately as possible. In addition, the sensitivity analysis can identify those parameters that have minimal affect on the model’s output and that perhaps identify places where the model can be simplified.

The sensitivity analysis consisted of the following rounds. The first round of sensitivity analysis used the reference parameter set we determined via preliminary investigation (above) and included single-parameter-variation simulations where in each simulation one parameter is changed 25% from its original value. The second round, also starting from the reference parameter set, changed **all** parameters simultaneously by rescaling the individual parameters using a normal distribution on logarithmic scale, which distributes the parameters across orders of magnitudes of their original values. This approach was used to ensure that the initial choice of parameters did not bias our ability to find better combinations of parameters by searching a much wider region of parameter space. From these simulations we selected several “good” and “bad” parameter sets and treated each of them as new base parameter sets with their own subgroup of simulations. Single-parameter-variation simulations were carried out with these new models. Sensitivity coefficient *J*_*k*_(*x*_*i*_), which captures the influence of model parameter *x*_*i*_ on *k*_*th*_ model output (*J*_*k*_), was calculated for each parameter in each group. The sensitivity is defined as:

$$J_{k}\left(x_{i}\right)=\frac{y_{k}\left(x_{i}+\Delta \right)-y_{k}\left(x_{i}\right)}{\Delta }\frac{x_{i}}{y_{k}\left(x_{i}\right)}$$ (3)

where Δ = 25% ⋅ *x*_*i*_ in our case. *J*_*k*_(*x*_*i*_) is an approximation of the Jacobian matrix term on a logarithmic scale:

$$J_{i,k}=\frac{\partial ln(y_{k})}{\partial ln(x_{i})}$$ (4)

We have also explored the *pairwise interactions* between model parameters. A pair of parameters interact if the simultaneous influence of the parameters on a model output is not additive. Pairwise interaction analysis, like parameter sensitivity analysis, helps to identity parameters, in this case pairs of parameters, that have the largest affects on the model’s output. In addition, a laboratory measurement that defines one of the two parameters in a pair will often greatly reduce the possible domain of the paired parameter. The procedures are similar to single-parameter-variation simulations except that in each simulation a pair of parameters is varied simultaneously. If the effects of a particular pair of parameters are independent from each other then the observed variation in the model output should consists of the linear addition of the variation of model output from the two individual single-parameter-variation simulations. Any discrepancy between the former and latter suggest nonlinear interactions between a pair of model parameters.

### Population Variability

Human populations show great variation in sensitivity to xenobiotics. The inter-individual variability is often large enough to give significantly different biological responses between individuals given the same dose. To test the feasibility of using our modeling framework to simulate populations we ran simulations in which all model parameters were simultaneously varied by a small amount (25%) around the reference simulation’s parameter values. The average serum concentration of APAP, APAPG and APAPS monitored for 1000 simulated individuals plausibly reproduced the *in vivo* ADME data.

Population analysis was carried out by choosing multipliers for each of the 38 parameters in the complete model. The multipliers were chosen from a normal distribution with a mean of 1 and a coefficient of variation of 25%. Note that this is similar to performing a parameter sensitivity analysis. We used 1000 randomly generated parameter sets to represent a population of individuals. For further details see section 4.2 in the S1 File.

## Results

### Standalone simulations

All three sub-models are standalone models that can be run without the framework of the complete multiscale model. Results for each of the three sub-models are described below. The ability to individually run the sub-models greatly facilitated their development and verification.

**Fig 4:**
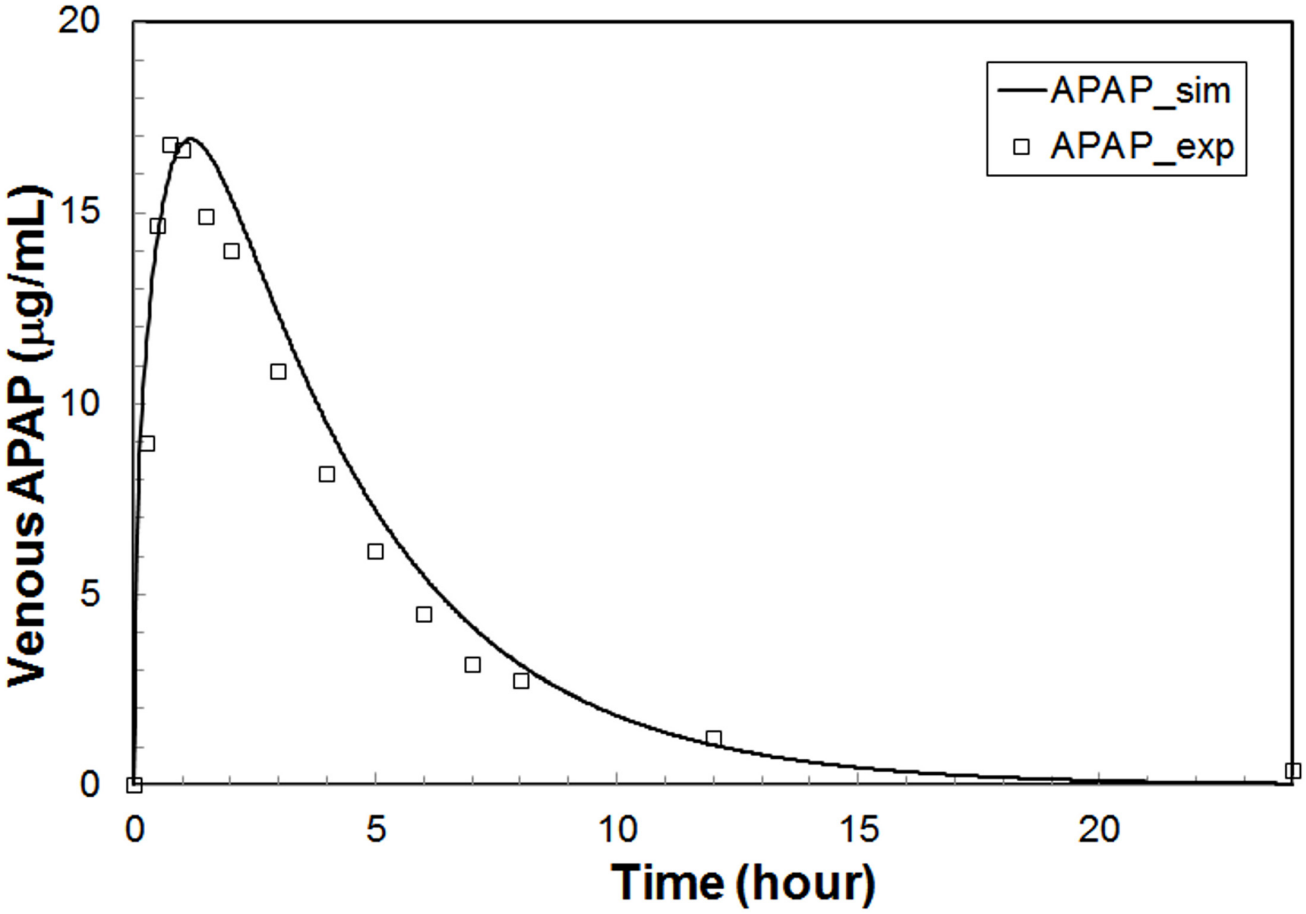
Standalone simulation of the PBPK module using the REFSIM parameters. This simulation is for a 1.4g oral APAP dose in a 70Kg male. The open symbols are *in vivo* data [58] and the line is the model output.

**Whole-body PBPK Module:** Standalone simulation results for the whole-body module are shown in Fig 4 using the parameter set REFSIM. This module’s SBML model was run directly in COPASI [37] (http://www.copasi.org/), an SBML compliant ODE modeling package.

**Fig 5:**
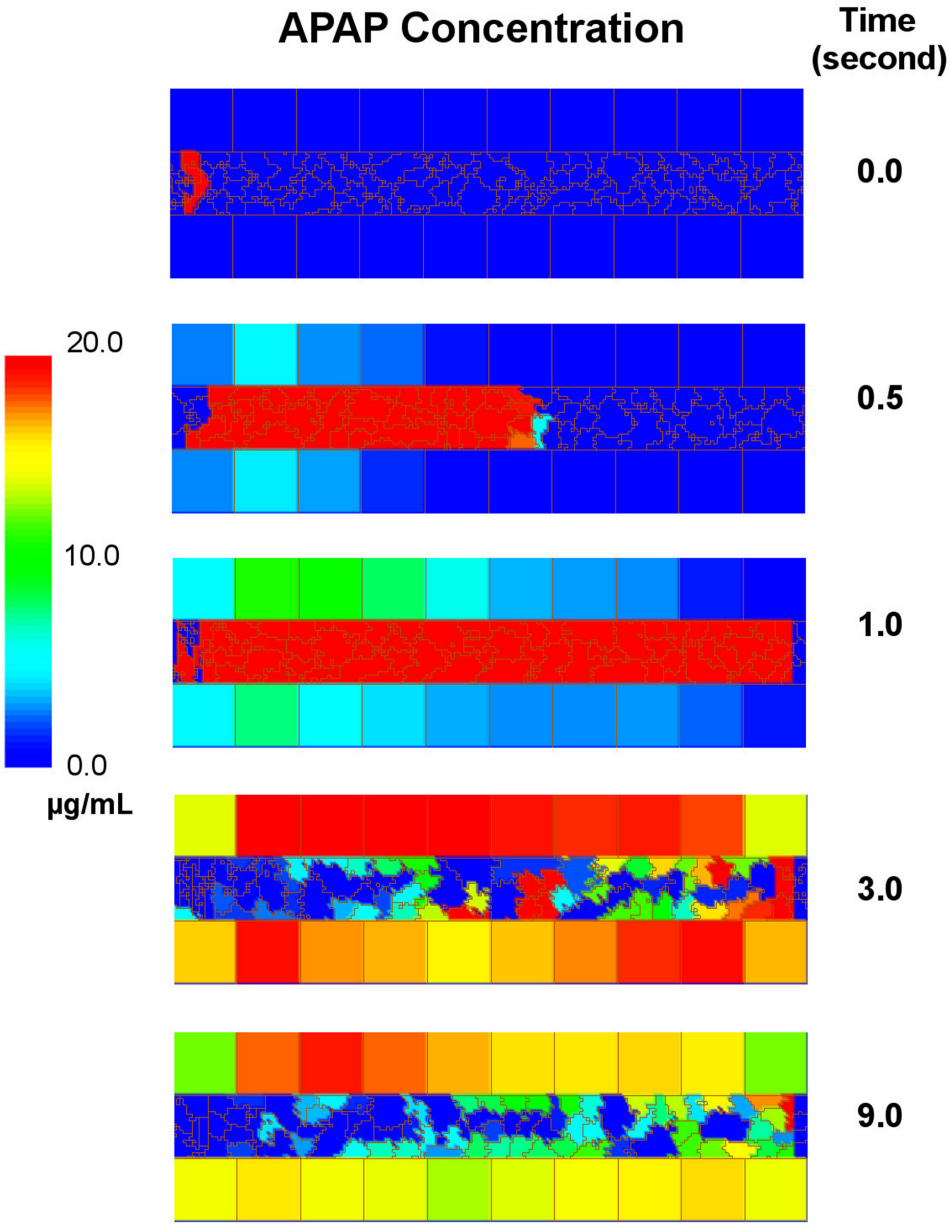
Time course of a standalone simulation of the sinusoid model in CC3D using the parameters set REFSIM. A simulated 3 second square pulse of APAP was pushed into the left end of the vessel lumen for three seconds starting one second into the simulation. The concentration of APAP in the blood and hepatocytes is given by the heat map scale at left and time progresses from top to bottom. Blood components are created at the periportal (left) end and a constant force is exerted on the blood components to induce blood flow through the simulated sinusoid. The temporal scales was adjusted so that the blood speed in the simulation was equivalent to 200 *μ*m/s, giving a transit time of a blood component through the sinusoid of one second.

**Multicell Liver Lobule Module:** Standalone simulation results for the multi-cell liver sinusoid module are shown in Fig 5 using the parameter set REFSIM. For the standalone simulation a square pulse of APAP was pushed into the sinusoid lumen for three seconds starting one second into the simulation as shown in Fig 5. During these 3 seconds, the APAP flux from blood to cells is dominant and APAP accumulates in the hepatocytes. This transfer is by both passive diffusion and active uptake of APAP. At later stages, when the APAP pulse has ended, intracellular APAP passively diffuses back out of the hepatocytes and into the blood flow. This reverse process is slower than the forward process since the forward process includes both passive diffusion and active import whereas the reverse process is passive diffusion only. An animation of this simulation is included (S1 Video).

**Fig 6:**
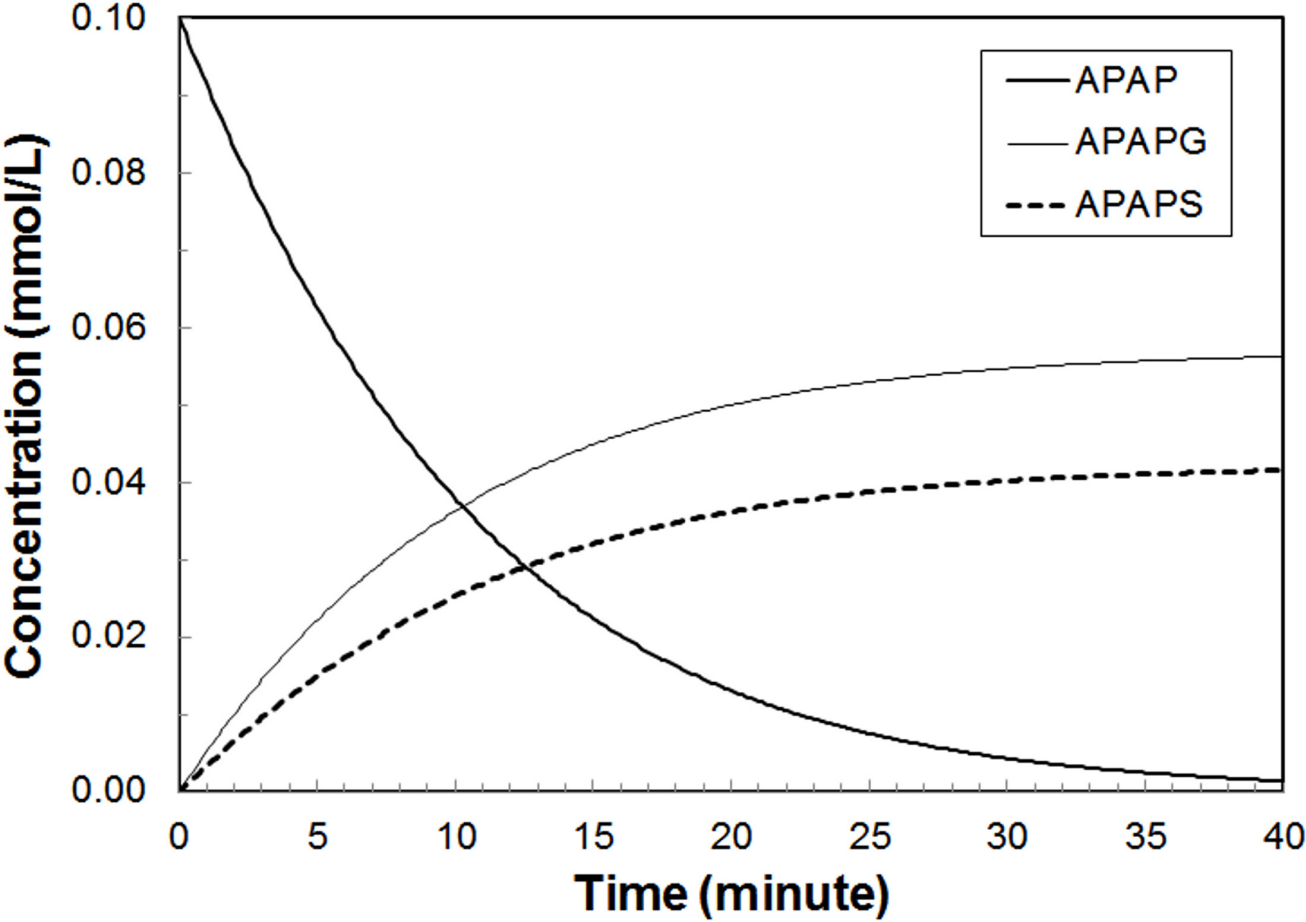
Standalone simulation of sub-cellular model. Results of the standalone run of the sub-cellular model using parameter set **REFSIM** and an initial concentration of APAP of 0.1mM (15*μ*g/ml).

**Subcellular Reaction Module:** Standalone simulation results for the sub-cellular metabolic reactions are shown in Fig 6. This SBML model was run directly in COPASI. The parameters were adjusted to reproduce the *in* *vivo* ratio of APAPG and APAPS.

Both the whole-body and sub-cellular SBML models can be easily replaced with more sophisticated models if additional data on the metabolic, uptake or clearance pathways becomes available. In addition, the SBML models can be changed to represent different species, or different chemical entities, without extensive modification of the complete multiscale model.

### Multiscale simulations

Linking the modules together for the three scales creates the complete in silico simulation framework providing a mechanism based model that incorporates effects at the various scales. In this study we used this framework to predict serum concentrations of APAP and the phase II metabolites following a therapeutic dose. This is as a first step in developing ADME and toxicity prediction simulations that may ultimately lead to improved techniques for prediction of toxicity of therapeutic agents and environmental toxicants while simultaneously reducing the need for animal toxicity studies.

We modeled APAP distribution following an oral dose of 20 mg/Kg in a normal male of 70 Kg body weight. To estimate an appropriate set of parameters, we validated the plasma concentrations of APAP, APAPG and APAPS against experimentally observed values [36, 58]. As described in the experimental section, we used *RMSE*_*i*_ as our primary metric to guide our selection of parameters by fitting our model to the experimental data in Critchley *et* *al*. [58]. We also used the maximum concentration (*Cmax*), time to arrive at maximum concentration (*Tmax*) and area under the curve (*AUC*) as model outputs for both comparison to the *in vivo* results and in the sensitivity analysis.

A single multiscale simulation requires 18 hours on a typical desktop machine using a two core, double threaded processor, e.g., an i3 level machine (CC3D is multicore and multithread capable). The majority of the compute times is taken up by the multicell model of blood flow through the simulated sinusoid. In addition, we made extensive use of Big Red II (BR2), Indiana University’s Cray XE6/XK7 supercomputer that has 21,824 processor cores. BR2 allowed us to run hundreds of simultaneous simulations, which was particular useful for parameter scanning and sensitivity analyses.

The whole-body and subcellular reaction kinetic models are deterministic when run on their own, whereas the multicell model has a stochastic component. The complete model therefore has a stochastic component. To explore the degree to which the stochasticity affects the model outputs (e.g. serum concentration versus time over a simulated 8 hour time period) we ran 50 simulations with the same parameter set and compared the results. Across all the model outputs (*RMSE*_*i*_, *Cmax*_*i*_, *Tmax*_*i*_, *AUC*_*i*_) the standard deviations were extremely small, in the range of part per billion (see S1 File).

**Fig 7:**
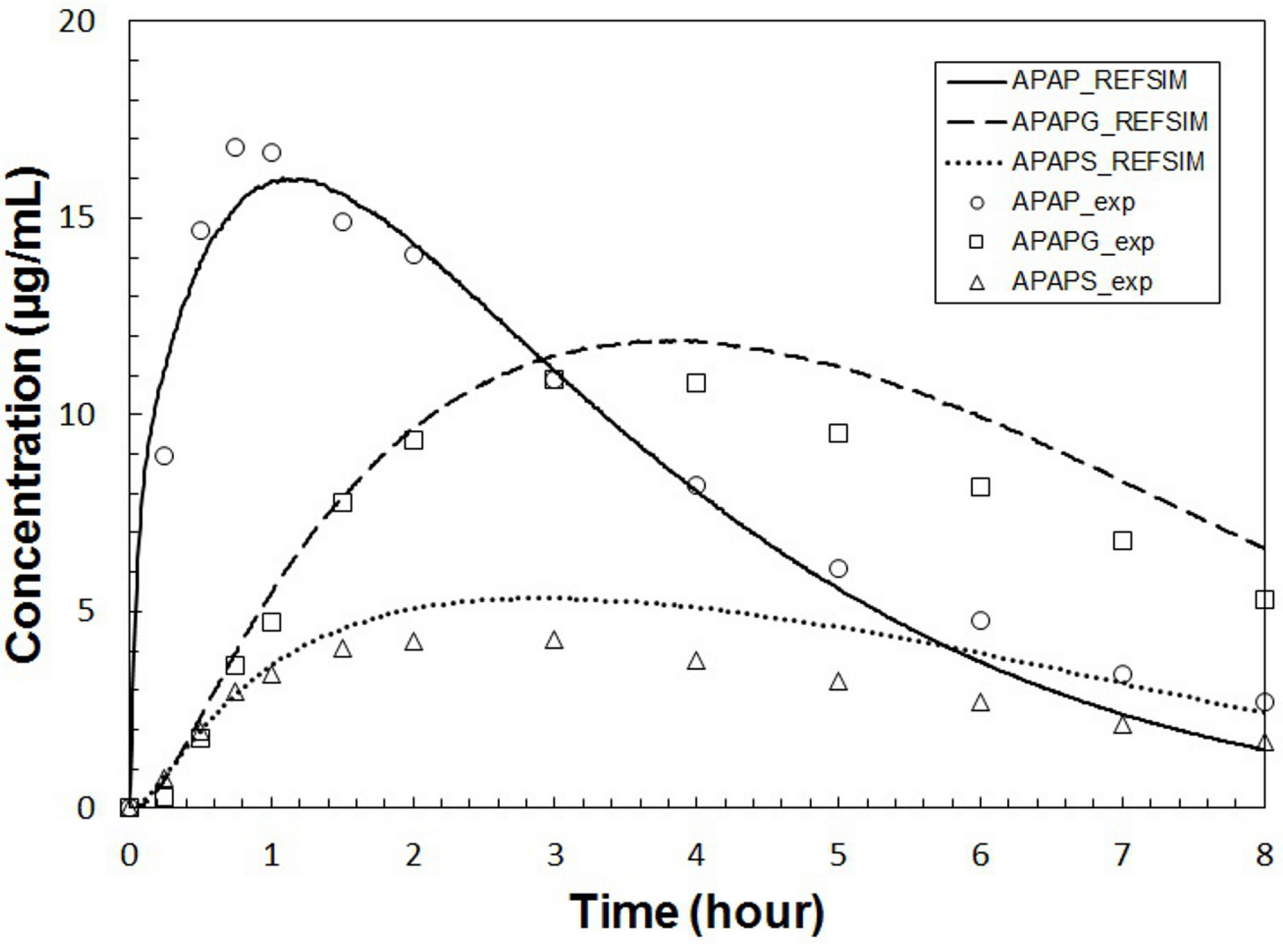
Plasma concentrations versus time for APAP and metabolites for REFSIM. Plasma concentrations versus time for APAP and metabolites simulated with the complete multiscale model using parameter set **REFSIM** (lines). Open symbols are *in* *vivo* average values from nine Caucasian subjects given a 1.4g oral dose of APAP [58].

**Fig 8:**
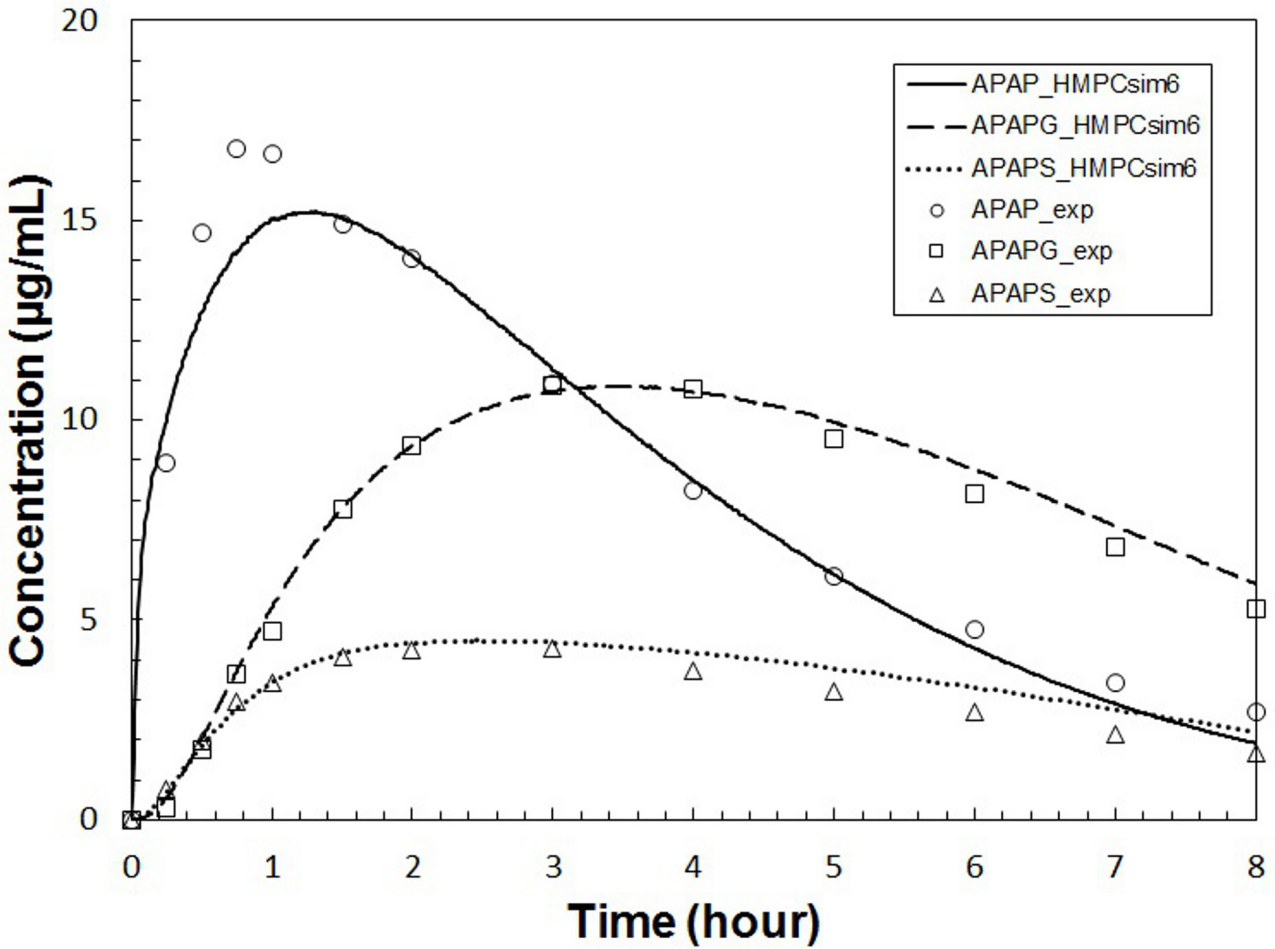
Plasma concentrations versus time for APAP and metabolites for HMPCsim6. Plasma concentrations versus time for APAP and metabolites simulated with the complete multiscale model using the best fit parameter set **HMPCsim6**. Symbols are as described in Fig 7.

With the initial parameter set (labeled “**REFSIM**”, see Tables 2–6), we obtained the plasma concentration curves for APAP, APAPG and APAPS in a simulated 8-hour time course as shown in Fig 7. An animation of a typical run, including data from all three modeling scales, is included (S2 Video). With minor changes (+/- 25%) of a portion of parameters, we generated another parameter set (labeled “**HMPCsim6**”) that gave a better fit with experimental data (Fig 8). The various named parameter sets are include in the S1 File. Despite the plausibly good fitting of the simulation result, **HMPCsim6** had notably weak performance in reproducing the peak of APAP curve, whereas the **REFSIM** parameter set had weak performance in reproducing the tails of all three concentration profiles.

**Fig 9:**
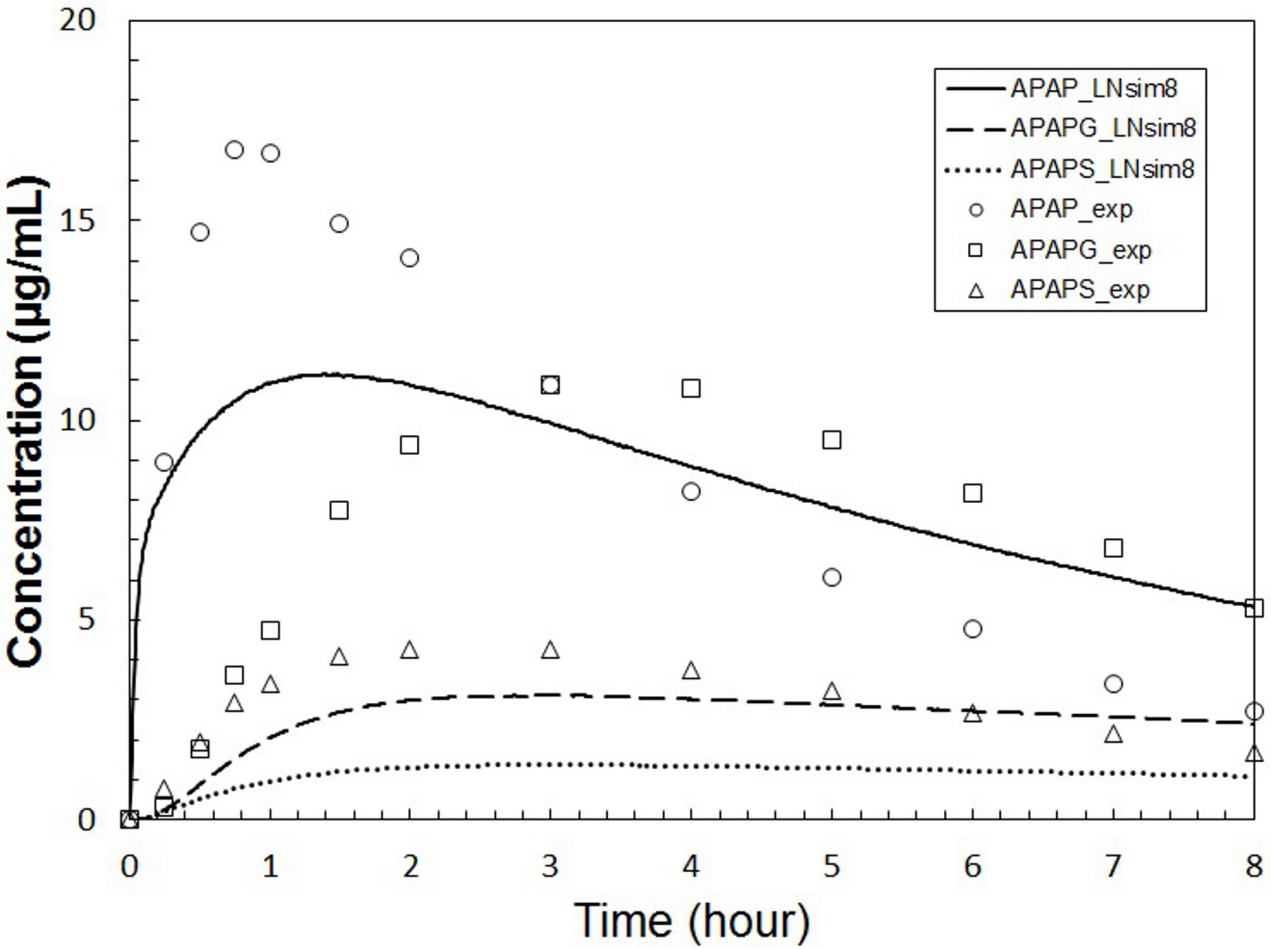
Plasma concentrations calculated using parameter set LNsim8. This parameter set represents a hypothetical chemical species with ADME behavior significantly different than APAP. Symbols are as described in Fig 7 and the APAP *in* *vivo* data is included for comparison.

**Fig 10:**
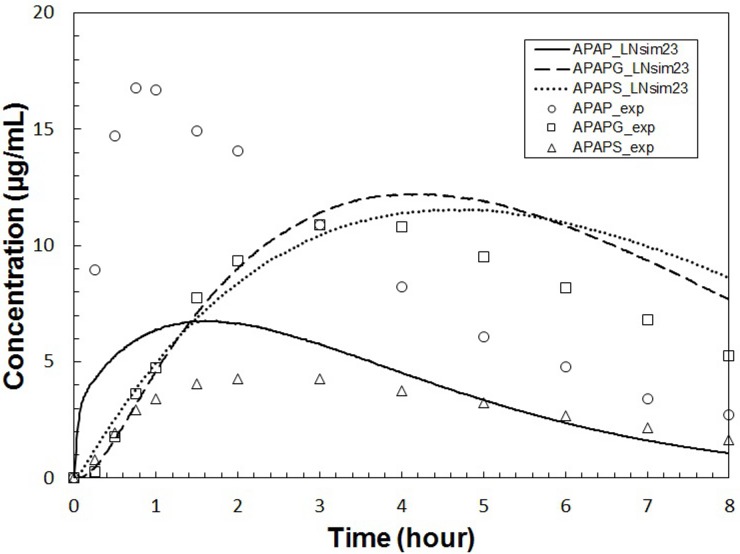
Plasma concentrations calculated using parameter set LNsim23. This parameter set represents a hypothetical chemical species with ADME behavior significantly different than APAP or the hypothetical species in Fig 9. Symbols are as described in Fig 7 and the APAP *in* *vivo* data is included for comparison.

To explore the numerical stability of our modeling framework across a larger parameter space, and to explore the parameter sensitivities as a function of the parameter space, we selected two other parameter sets (named “**LNsim8**” and “**LNsim23**”) whose ADME profiles are significantly different than the *in* *vivo* APAP data (see Figs 9 and 10). These two parameter sets can be thought of as representing chemical species with radically different ADME profiles compared to APAP. Both the **LNsim8** and **LNsim23** parameter sets are characterized by shifting of the equilibrium from the blood towards the rest of the body (pbpk_Kr2p). The **LNsim8** is also characterized by low active transport of APAP from the blood into the hepatocytes (cc3d_Vmax_AT_APAP) and faster formation of APAPG (sc_Vmax_GLUC). The **LNsim23** is further characterized by a slow rate of absorption in the gut (pbpk_kGutabs) and low kidney clearance rate for the glucuronide metabolite (pbpk_QgfrG). These two diverse parameter sets were also subjected to sensitivity analyses allowing us to compare the model’s parameter sensitivities across a range of ADME profiles. A table listing the complete set of parameters and sensitivities for all four fixed points is given in the S1 File.

**Fig 11:**
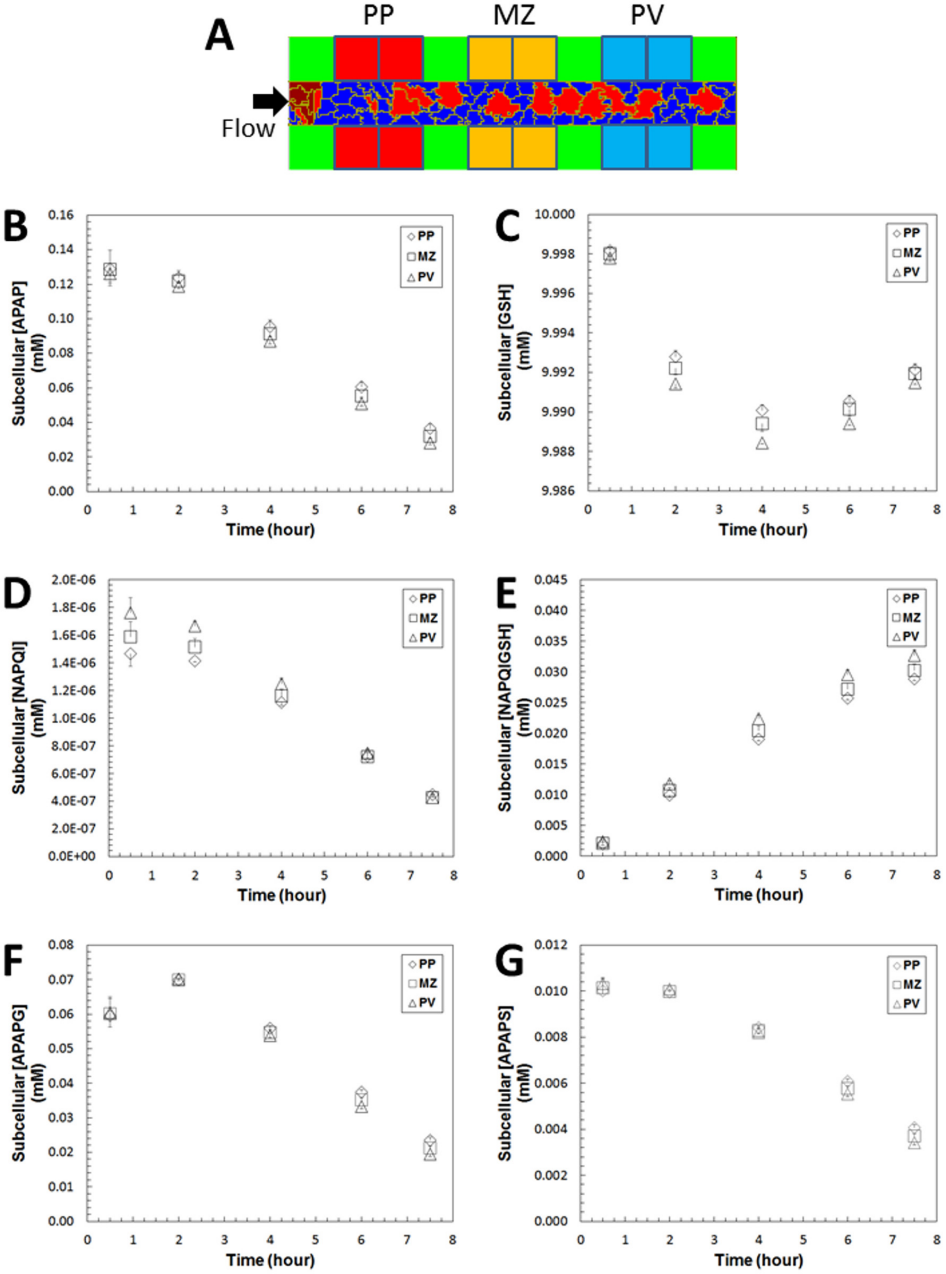
Subcellular concentration of APAP and Phase I metabolites in REFSIM simulation. Four cells were monitored in each of the three regions; (A), periportal (PP), midzonal (MZ) and perivenous (PV), and the average concentration in each group is plotted. (B) APAP, (C) GSH, (D) NAPQI, (E) NAPQI-GSH, (E) APAP-Glucuronide, and (E) APAP-Sulfate. Error bars are the standard deviation of the four cells in a region.

**Fig 12:**
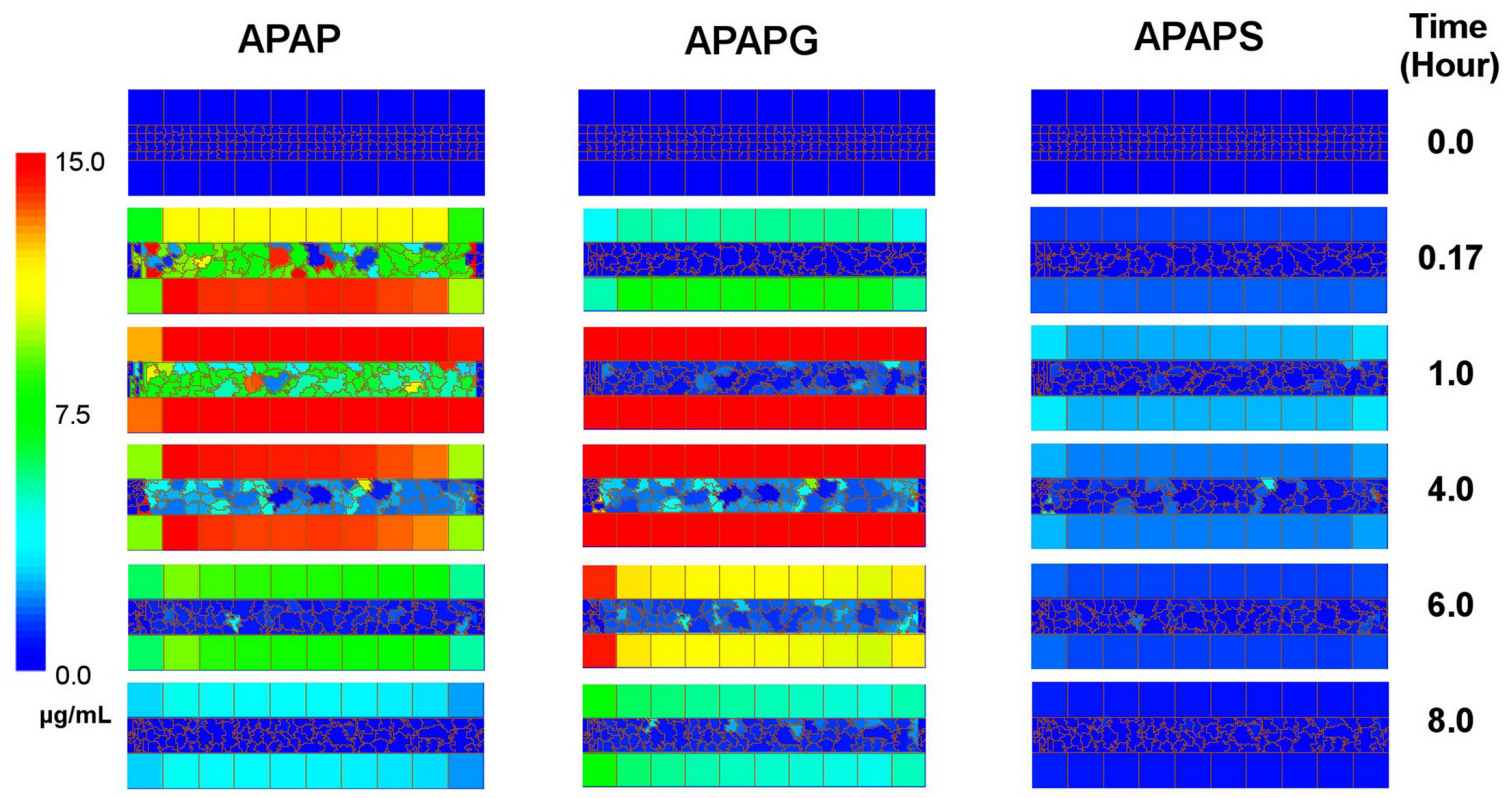
Sinusoid chemical concentration time course. Time course snapshots of the chemical field for the multi-cell scale of the complete model using parameter set **REFSIM**. Concentrations of APAP (left), APAPG (middle) and APAPS (right) are indicated by the color scale. This time course spans 8 simulated hours top to bottom.

Examination of the results for the complete model using either **REFSIM** or **HMPCsim6** shows that the subcellular concentration of APAP, APAPG and APAPS vary little along the sinusoid (Fig 11). However, NAPQI concentration is higher in pericentral hepatocytes than in periportal hepatocytes as shown in Fig 11. Largely due to the imposed zonation of CYP2E1 concentration, hepatocytes with higher Phase I metabolic capability at the centrilobular region had slightly greater consumption of cellular GSH and greater formation of NAPQI-GSH. Unlike the pattern of Phase I metabolites, the Phase II metabolites (APAPG and APAPS) were higher in the periportal region, due to the higher uptake of APAP in this region (Fig 11F and 11G). The subcellular concentrations of APAP, APAPG and APAPS across the simulated sinusoid were also visualized at several time points as shown in Fig 12. The lateral (top to bottom) asymmetry in the APAP concentration at 0.17 hours is due to the different concentrations of APAP in the arterial versus venous inflows to the sinusoid.

### Sensitivity analysis

**Fig 13:**
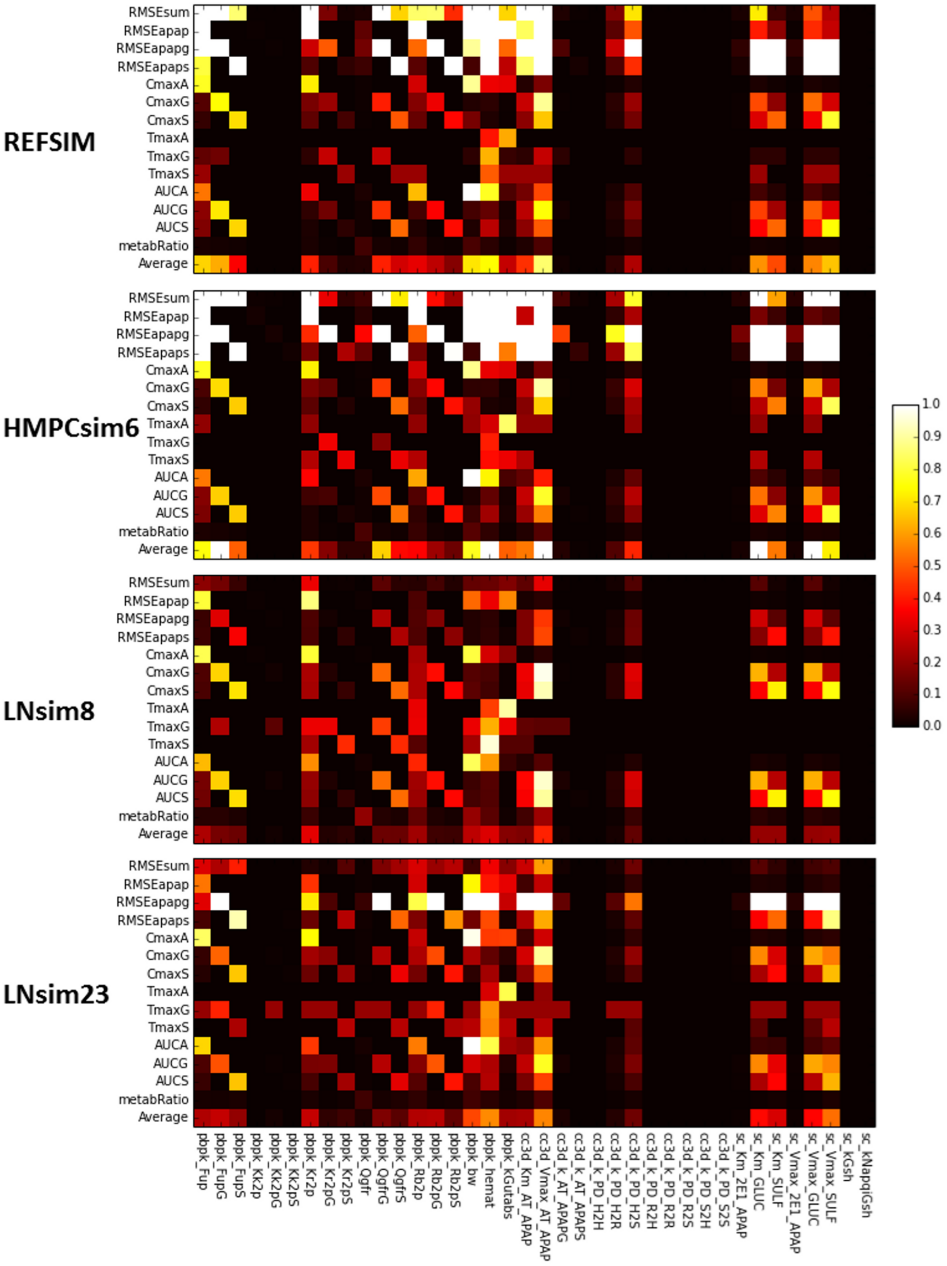
Fixed point sensitivity of model outputs to single parameter variations. The horizontal axis lists the model’s parameters grouped by the sub-model. Parameters for the PBPK model start with “pbpk_”, the sinusoid models with “cc3d_” and the subcellular model with “sc_”. The vertical axis lists the model’s outputs duplicated for each of the input parameter sets. The “Average” row is the average of all the sensitivities in the column for the particular parameter set. Each square in the heat map is the relative change of a model output divided by the relative change of the model parameter in single-parameter-variation simulation. Dark space indicates little influence of a parameter on the model outputs, bright regions reflect strong influence of a parameter and white regions represent sensitivities greater than one.

**Single-parameter variation:** We performed sensitivity analyses around the fixed points (base parameter sets) **REFSIM**, **HMPCsim6**, **LNsim8** and **LNsim23**. First, single-parameter-variation simulations were carried out around those nominal parameter values, and sensitivity indices were derived from the simulation results. The heat maps in Fig 13 reflects the extent to which the various model outputs change in response to single parameter changes about these four fixed points. Brighter regions indicate greater sensitivity of model outputs to individual parameters. As the most global measurement, RMSEsum captures the parameter’s influence on the serum profiles for all three compounds (APAP, APAPG, APAPS) when trying to reproduce the human ADME for APAP. As shown in the heat map, approximately half of the parameters contribute significantly to the RMSEsum value.

**Whole-body scale sensitivities:** Not surprisingly, two of the most influential parameters are body weight (pbpk_bw) and hematocrit (pbpk_hemat). Body weight affects many aspects in the model, such as organ size and blood flows, and accordingly directly affects the APAP distribution and concentration. The hematocrit is the ratio of RBC volume to blood volume, which directly affects the effective blood concentration and the uptake rate of APAP in both the liver and kidney compartments. The rate of gut absorption (pbpk_kGutabs), the three Qgfr (pbpk_Qgfr, pbpk_QgfrG, pbpk_QgfrS) values, and the parameters that control the partitioning of APAP and metabolites between the blood components (pbpk_Fup, pbpk_FupG, pbpk_FupS, pbpk_Kr2p) all significantly impact RMSEsum.

**Tissue scale sensitivities:** At the multicell/tissue scale the two Michaelis-Menten parameters (cc3d_Km_AT_APAP, cc3d_Vmax_AT_APAP) controlling the active uptake of APAP by the hepatocytes are most influential, and they strongly affect not only the RMSEsum but RMSE for the three individual compounds. In addition, the forward rate constant for transfer of APAP out of the hepatocytes and back into the blood flow (cc3D_k_PD_H2S) appreciably affects the model’s outputs. Interestingly, the rest of the rate constants for transfer between the tissue scale components (Fig 3) have minimal affects on the model’s outputs. This may indicate that the blood flow model can be simplified, perhaps by increasing the size of the serum portions and/or aggregating the serum and RBCs into a single component.

**Subcellular metabolism scale sensitivities:** Surprisingly, the model is relative insensitive to the rate of CYP metabolism as controlled by the two Michaelis-Menten parameters sc_Km_2E1_APAP and sc_Vmax_2E1_APAP. The RMSEs are much more sensitive to the parameters controlling the two Phase II reactions. The parameter sensitivities for parameters that directly control the Phase II reactions show output sensitivities that partition to the respective metabolites. For example, the parameters controlling sulfation strongly control RMSEapaps, CmaxS, TmaxS and AUCS and, by competition, the corresponding model outputs for APAPG.

In addition to the group of parameters that influence all the RMSEs, there are also sets of parameters that show a modular pattern of importance. These subsets of parameters further falls into two classifications. The first classification shows strict compound-specific grouping of heat blocks, and most of the PBPK module parameters belong to this group. For instance, model measurements RMSEapapg, CmaxG, AUCG are highly sensitive to parameters FupG, QgfrG and Rb2pG, while indifferent to corresponding parameters related to APAPS, and vice versa. In contrast, the second classification shows cross-compound influence in the heat map. The Michaelis-Menten constants describing the Phase II metabolism of APAP are in this group. This indicates a competition between the two pathways to process APAP. For example, a perturbation in a parameter controlling glucuronidation (APAPG) results in an opposite effect on the production of Sulfate conjugates (APAPS).

**Insensitive Parameters:** 17 out of 38 parameters (45%) examined have minimal impact on the model’s outputs for the **REFSIM** fixed point. Most of these parameters are for passive transfers, though the rate constant for CYP metabolism (mentioned above) and the rate constants for GSH synthesis and the conjugation of NAPQI with GSH are also in this group. These later parameter insensitivities may reflect the relatively low importance of Phase I metabolism on the ADME of APAP at pharmacological doses and these sensitivities might not be the same for overdoses of APAP.

The sensitivity heat maps for different base parameter sets (different fixed points) show generally similar sensitivity patterns (Fig 13). The observations outlined above, in general, were observed in all four maps. Two of the fixed point parameter sets (**REFSIM**, **HMPCsim6**) are for parameter sets that adequately reproduce the APAP *in vivo* ADME data. The other two parameter sets (**LNsim8** and **LNsim23**) were purposely chosen to represent hypothetical compounds with ADME profiles radically different from APAP. Though there are differences between the sensitivities of these “different compound’s” parameter sensitivities and the APAP specific parameter sets, the differences are generally minor. The major differences are in reproducing the highly compound-specific model outputs related to the *RMSE*_*i*_ values.

**Correlations between sensitivities across different fixed points:** To explore the similarity between the parameter sensitivities across the four fixed points we calculated the correlation coefficients between the sensitivity matrices. We calculated correlations for the entire tables (“All”), just using the “Average” sensitivity row (which omits the RMSEsum and metabRatio rows) and just using the rows excluding all the RMSEs and “Average”. In the last case, the correlation is done without taking into account how well the particular parameter set reproduces the APAP ADME data, and therefore represent the sensitivity of the model’s response regardless of how well it reproduces the APAP ADME data. As shown in Table 7, the “Average’ sensitivities correlate well, as does the third set of correlations (which omit the sensitivity to the APAP ADME data), across all four parameter sets even though the **LNsim8** and **LNsim23** sets represents compounds with significantly different ADME profiles than APAP. Across “All” model outputs the **REFSIM** and **HMPCsim6** parameter sets correlate very well with each other while the **LNsim8** and **LNsim23** sets correlate much less with both the APAP like parameter sets and with each other. *These results suggests that, for these four fixed points, the model sensitivities for outputs that are not calculated relative to a particular compound’s in vivo ADME profile (RMSEs) are similar across a range of ADME behaviors*.

**Table 7:** Correlation Coefficients and R^2^ values between sensitivities for the four parameter sets.

All Sensitivities	REFSIM	HMPCsim6	LNsim8	LNsim23
**REFSIM**	1	**0.72**	**0.58**	**0.58**
**HMPCsim6**	0.85	1	**0.12**	**0.53**
**LNsim8**	0.47	0.35	1	**0.32**
**LNsim23**	0.76	0.73	0.57	1
**Average Sensitivities**	**REFSIM**	**HMPCsim6**	**LNsim8**	**LNsim23**
**REFSIM**	1	**0.88**	**0.88**	**0.90**
**HMPCsim6**	0.94	1	**0.83**	**0.83**
**LNsim8**	0.94	0.91	1	**0.85**
**LNsim23**	0.95	0.91	0.92	1
**w/o RMSEs and Average**	**REFSIM**	**HMPCsim6**	**LNsim8**	**LNsim23**
**REFSIM**	1	**0.92**	**0.85**	**0.86**
**HMPCsim6**	0.96	1	**0.85**	**0.79**
**LNsim8**	0.92	0.92	1	**0.76**
**LNsim23**	0.93	0.89	0.87	1

The sensitivities are from Fig 13. Values below the diagonal are correlations coefficients and above the diagonal R^2^’s. The sub-table labeled “All Sensitivities” is the correlation for comparisons between the parameter sensitivities across all parameters and all model outputs (comparisons of 38x15 matrices). The sub-table labeled “Average Sensitivities” is the correlation for comparisons between the parameter sensitivities across all parameter sets and the model output“Average” rows (comparisons of 1x15 vectors). The “w/o RMSEs…” sub-table is similar to the “All” table but excludes the RMSEs (APAP ADME specific model outputs) and the “Average” rows from the sensitivity matrices (comparisons of 38x10 matrices).

**Fig 14:**
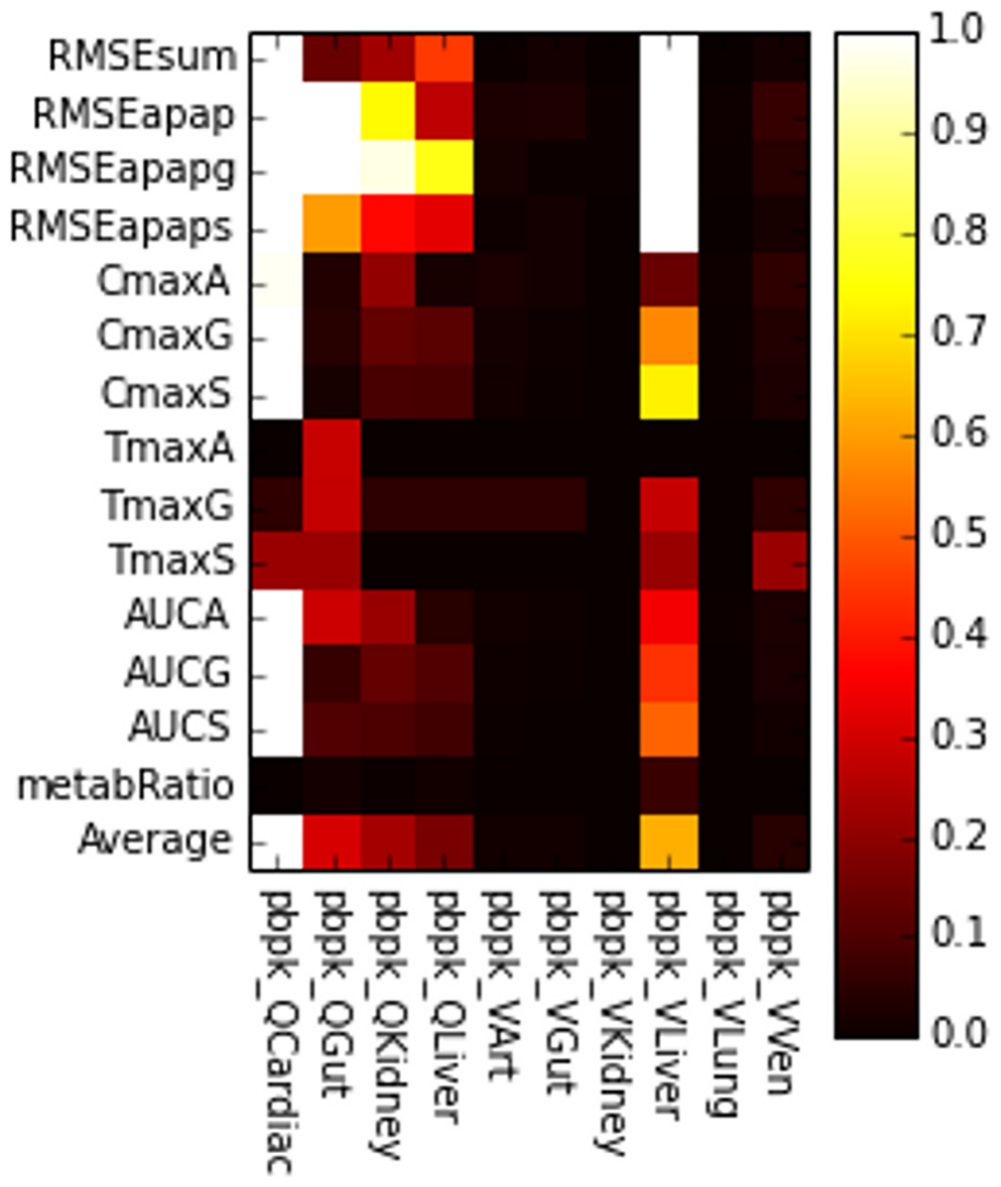
Compound-independent parameters sensitivity comparison. Comparison of the sensitivities for the compound-independent parameters in the whole-body PBPK model about the fixed point **REFSIM**. Axis are as described in Fig 13.

**Sensitivities for the compound independent parameters in the PBPK model:** The whole-Body PBPK models contain a number of parameters that depend on the individual being modeled and are independent of the chemical species (Table 2). The sensitivity results for the PBPK blood flow rates and compartment volumes are shown in Fig 14. By far the most influential parameter is the cardiac output (pbpk_QCardiac), which strongly affects most of the model’s outputs. The model is insensitive to the compartment volumes, over the range of +/-25%, with the exception of the volume of the liver (pbpk_VLiver).

**Fig 15:**
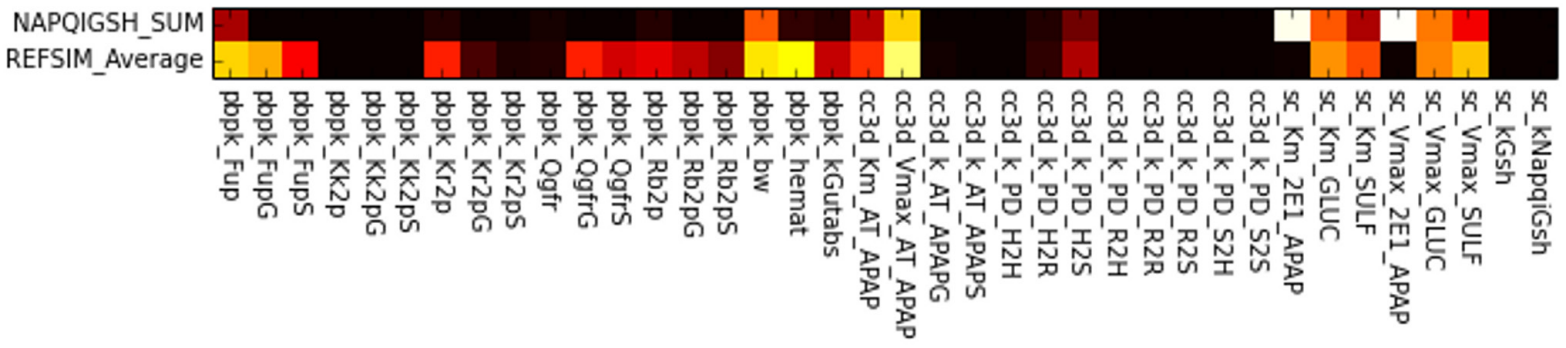
Sensitivity comparisons for formation of NAPQI-GSH. Comparison of the sensitivities for the formation of NAPQI-GSH (NAPQIGSH_Sum) versus the average parameter sensitivities about the fixed point **REFSIM**. Axis are as described in Fig 13.

**NAPQI Conjugation Sensitivities:** Though our model was not specifically designed nor calibrated for toxic doses of APAP it does include the Phase I metabolic pathway leading to NAPQI and subsequent formation of NAPQI-GSH. Fig 15 shows a comparison of the parameter sensitivities between the total amount of NAPQI-GSH formed (the sum across all the simulated hepatocytes) and the average sensitivity across all the other model outputs. As expected, the Michaelis-Menten parameters for the CYP2E1 conversion of APAP to NAPQI are the most important parameters controlling the amount of NAPQI-GSH formed. The other sensitive parameters are related to the two Phase II reactions (which are in competition with the Phase I reaction), the rate of uptake of APAP from the blood, and the body weight.

**Fig 16:**
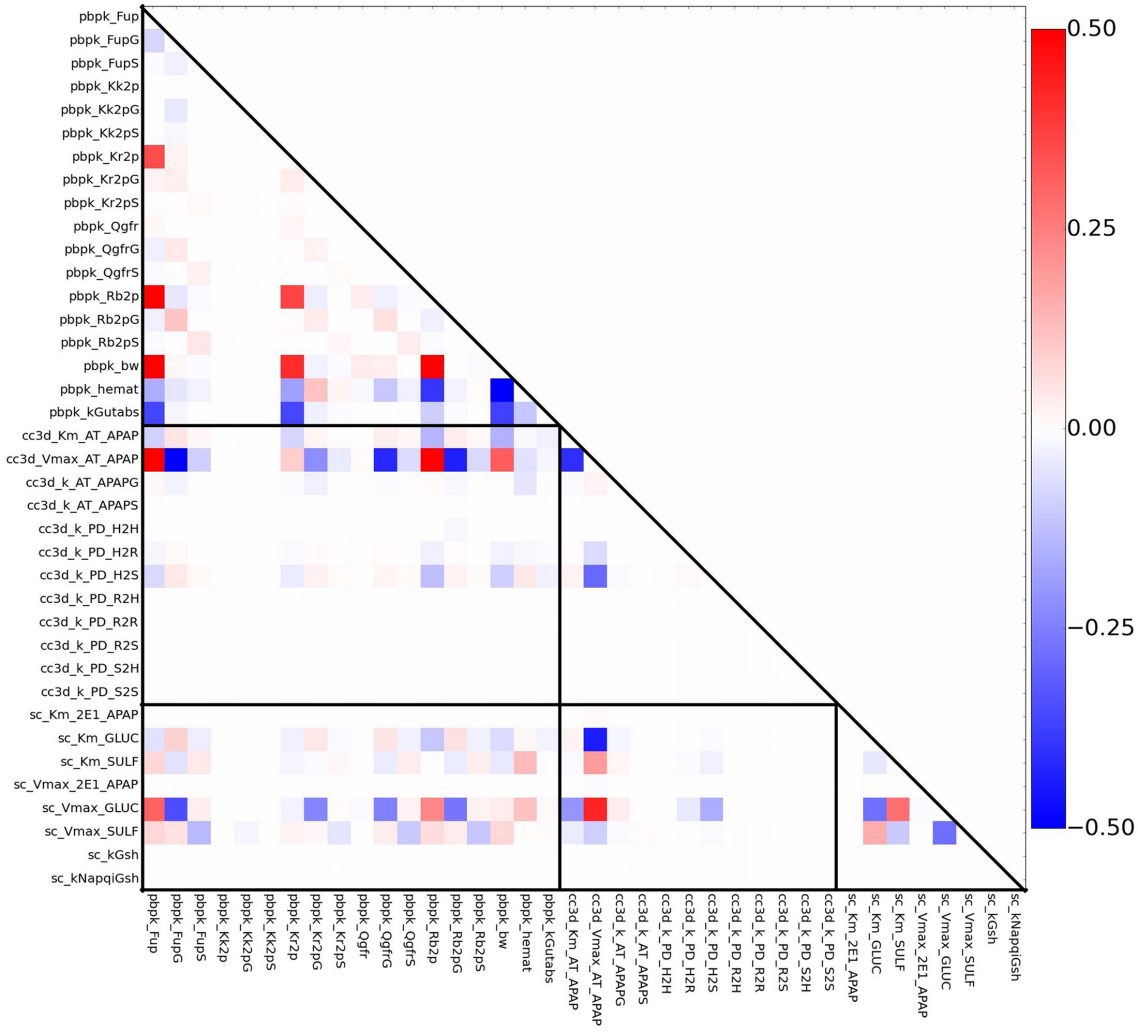
Nonlinear sensitivity of model outputs to PPV. Pairwise parameter interactions for model output RMSEsum. Each square in the heat map is the difference between the relative changes in model output (RMSEsum) for two individual single-parameter-variation simulations compared with the pairwise-parameter-variation simulation. White space indicates little interaction between a pair of parameters, red or blue regions reflect strong, non-linear parameter interactions. Horizontal and vertical lines separate the parameters of the three model scales with rectangular regions showing interactions that cross model scales and triangular regions for interactions within a model scale. Parameter variations are about the fixed point **REFSIM**.

**Pairwise-parameter-variation (PPV):** PPV simulations were carried out for parameter set **REFSIM** (Fig 16). Because of the large number of permutations of PPV coupled with the multiple model outputs we show here just the sensitivities of the RMSEsum. Each PPV interaction term is calculated by subtracting the observed relative variation of RMSEsum in the PPV simulations from the relative variation of RMSEsum in two single parameter variation simulations. If two parameters do not interact (that is, the two parameter’s effects are independent) then the PPV value is zero. If the two parameters do interact then the PPV value is non-zero and the sign indicates the relative direction of the interaction. As shown in Fig 16 less than half of the PPV displayed strong interactions. As an example of a strong parameter interaction, near the center of Fig 16, the cross interaction of the Michaelis-Menten parameters for the active transport of APAP from the blood into the hepatocytes (cc3d_Km_AT_APAP and cc3d_Vmax_AT_APAP) was of the strongest negative PPV coefficient. Since these two parameters occur in the same rate equation, but have opposite effects on the rate, they strongly interact over the range of parameters explored, giving the observed negative interaction coefficient.

An additional example of strong term interaction, in this case positive, is the interaction between the body weight (pbpk_bw) and both the hematocrit and rate of gut absorption (pbpk_hemat and pbpk_kGutabs).

Finally, there is a strong interactions between the PBPK module’s Fup and the partition coefficient between the rest of the body and the plasma (Kr2p) and in the ratio of blood concentration to plasma concentration (Rb2p) parameters. Examination of the PBPK ODEs shows that those three parameters are indeed related by nonlinear relationship.

In the above cases of strong interactions in PPVs, the nature of the interaction, and indeed the ability to predict the interaction, is often obvious from the particular module’s structure. For example, Michaelis-Menten Vmax and Km values will generally be strongly coupled. Perhaps the more interesting interactions are those that occur across modules, and hence, across scales. These interactions between modeling scales are much less obvious *de* *novo*. In Fig 16 triangular regions along the diagonal are interactions between parameters within the same module whereas rectangular regions are interactions between parameters of different modules. In our model we see large cross-module PPV coefficients for the interaction of the transport rate from the serum to hepatocytes (cc3d_Vmax_at_APAP), and to a lesser extent the related Km, with several parameters in the PBPK module (Fup, blood binding and tissue partition coefficients) and subcellular module (the Michaelis-Menten Vmax and Km for the Phase II reactions). In general, *de* *novo* prediction of inter-module parameter interactions is much less straightforward than intra-module.

### Population Variability

**Fig 17:**
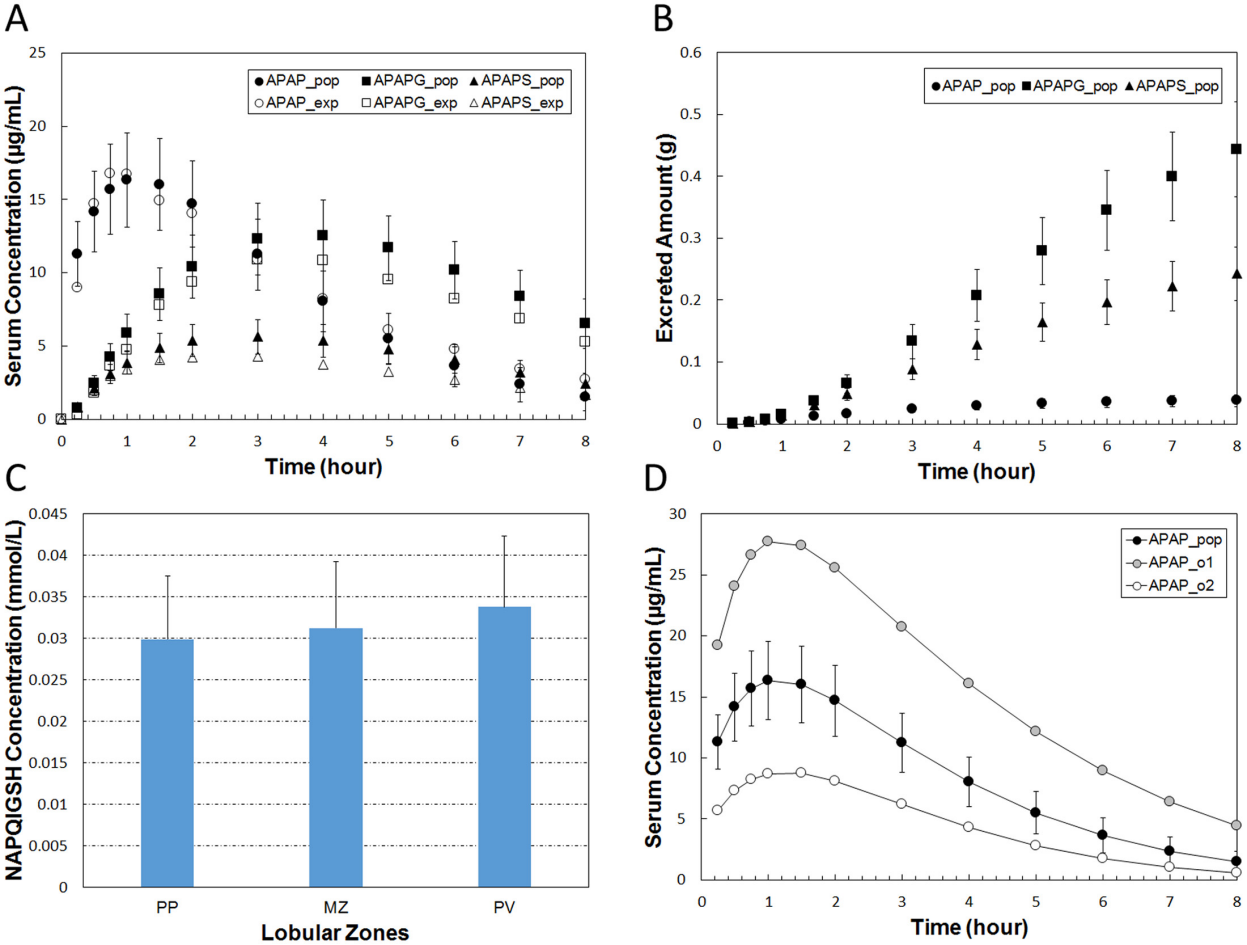
Population variability based on 1000 simulated individuals. The population was generated by assuming that for each in silico individual, each parameter was within a truncated normal distribution with coefficient of variation of 25% around the base parameter from the parameter set **REFSIM**. (A) Average serum concentrations of APAP and Phase II metabolites for the simulated population. Open symbols are human *in vivo* data, closed symbols simulation results. (B) Average urinary excretion of APAP and Phase II metabolites for the simulated population. (C) Average cellular NAPQI-GSH for the population (zones as defined in Fig 11A). (D) Comparison of the average response (closed symbols with error bars) of the simulated population with the simulated individuals that deviate the most, high and low, from the population average (symbols without error bars). Error bars are standard deviations.

Human populations show great variation in both exposure and sensitivity to APAP [58]. To extend our framework from modeling an individual to modeling a population, we ran simulations in which all model parameters were simultaneously varied by a small amount (25%) around the reference simulation’s parameter values. The average serum concentration of APAP, APAPG and APAPS monitored for 1000 simulated individuals plausibly reproduced the *in vivo* ADME data (Fig 17A). We also found that, on average, nearly 60% of APAP dose ends up as APAPG, more than 30% APAPS, and less than 10% as APAP (Fig 17B), which is consistent with the metabolic ratios seen *in* *vivo*. We also monitored the subcellular NAPQI-GSH concentration to explore the zonal extent of toxicity across the simulated population. The subcellular NAPQI-GSH level increased along the portal to central vein axis, though the difference is mild when compared to the population standard deviation (Fig 17C). The zonal difference of NAPQI concentration is partly due to the imposed zonation of CYP2E1 expression and partly due to the APAP gradient along the sinusoid.

In our simulated population the standard deviations are fairly large (Fig 17A), though the resulting variability in, for example, the serum concentration versus time for APAP is similar to what is typically seen clinically in ADME studies with a diverse population. However, the variability in Fig 17A is considerably larger than in the data of [58]. The data we used is from a population with fairly small diversity: Caucasian, n = 9, ages 23–44, body weight 55–97 Kg and male–female ratio of 6:3 [58]. Inclusion of the other population in the study (Hong Kong Chinese, n = 11, ages 21–32, body weight 46–71 Kg, male–female ratio 5:6) gives standard deviations approaching what we see for our simulated population [58]. This observation suggests that our simplistic method of generating a population gives inter-individual variabilities that are similar to a highly diverse population.

It is interesting to examine the most extreme outliers in our simulated population. Fig 17D shows a plot of serum levels for the entire population compared to the simulated individuals with the largest deviations from the population mean in terms of serum level of APAP versus time. These “one in a thousand” individuals have peak serum levels and AUCs that are significantly different than the population average. The simulated individual with the highest peak APAP serum concentration has a level nearly twice that of the population average whereas the simulated individual with the lowest level has half the peak level. These particular simulated individuals would be expected to respond to both therapeutic and toxic doses of APAP significantly different than the average individual. However, neither of these two simulated individuals has any single parameter that is outside a reasonable range of inter-individual variability. The most notable differences between the two outliers and the rest of the population is their body weights. The simulated individual with the highest APAP serum levels has a body weight of 57 Kg and the simulated individual with the lowest serum levels a body weight of 86Kg.

### Advantages of Separate Models for the Biological Scales

A significant advantage to our approach of using standard modeling modalities for each scale of the multi-scale model is the ease of exploring changes in parameters or model structure at a particular scale without needing to run the entire multiscale model. For example, calibration of the subcellular metabolic reactions using data from *in vitro* studies, such as metabolism in isolated hepatocytes, provides a partial route for *in vitro* to *in vivo* extrapolation (IVIVE) [61]. In addition, our approach allows us to leverage existing tools for tasks such as parameter estimation. As an example of this we have re-examined the initial step of the APAP PBPK model; the rate of absorption of APAP from the gut lumen. Our PBPK model is based upon published models [19, 31, 32] and assumes that the rate of absorption of APAP from the gut lumen follows simple first order kinetics. Close examination of published ADME data for therapeutic oral doses of APAP in humans determined by Prescott [36] strongly suggest that the uptake of APAP from the gut lumen is a transporter mediated process and not a first order process. In addition, it appear that at therapeutic doses the gut uptake transporters are saturated. From the data in [36] it appears that there is a 9 minute delay, presumably due to gastric emptying, followed by a linear increase in serum concentration giving a constant rate of uptake. Zeroth order kinetics can be modeled using Michaelis –Menten kinetics with *V*_*max*_ equal to the observed slope and *K*_*m*_ set to a value small compared to the initial concentration. We have modified the PBPK model and estimated the *V*_*max*_ and *k*_*m*_ by fitting the Prescott data using COPASI. The zeroth order model gives a much better fit to the experimental data than does the first order model used previously. Significant involvement of a saturable transporter in the gut for APAP has not generally been recognized [10]. The presence of a saturable transporter in the gut will significantly affect the serum levels of APAP in overdose cases.

This is a clear example where mathematical modeling of biological processes can extend our understanding of the underlying biology. In addition, this clearly demonstrates the power of having sub-models that can be run on their own since that allows simple tuning of one sub-model prior to incorporating the changes into the complete multi-scale model.

## Discussion

We have developed a multiscale modeling framework for xenobiotics. We focused on modeling the disposition of pharmacological doses of APAP in humans. However, this framework can be extended to other xenobiotics and species. Our multiscale framework is built upon open-source software packages. The use of open-source software facilitates the transparent use, distribution and reuse of multiscale models. In addition, we have separated the three main biological scales (whole-body, tissue, and subcellular) and modeled those scales in appropriate modeling platforms that leverages existing modeling modalities and tools. In particular, both the whole-body and subcellular reaction kinetic schemes are modeled in SBML, which allows us to leverage the extensive suite of SBML tools and allowed us to incorporate biological annotations directly in the SBML source code.

**Blood flow simulation using Cellular Potts Model:** At the tissue level we simulate blood flow using the Cellular Potts Model implemented in CC3D. Compared to other modeling approaches, that use advection-diffusion equations to model flow through a porous medium [24], or through pipe lined with a small number of compartments [32], our approach includes explicit RBC and serum portions and couples the local blood concentration to individual, explicitly modeled hepatocytes, lining a simulated sinusoid. Blood is a biphasic fluid containing both RBCs (with diameters similar to the diameter of a sinusoid blood vessel) and blood plasma whose biophysical properties differ. RBCs tends to travel at the center of blood vessel [62], which produces a cell-free layer near the vessel wall. In addition, small molecules typically have different partitioning and protein affinities within RBCs vs. blood plasma. This presents a challenge to using advection-diffusion equations to describe blood-borne small molecules, particular at the level of distribution into individual hepatocytes. Our simulated blood flow is composed of simulated cells representing RBCs and serum portions. Adhesive energies between RBCs, serum portions and hepatocytes influence the behavior of the blood flow. In our simulation, RBCs are less adhesive to hepatocytes and travel primarily at the center of the simulated sinusoid (see S1 Video). Both the initial concentrations within RBCs and serum portions, as well as the transfer from one to another, are tunable according to the biochemical properties of the compound being modeled, which together with RBC-plasma patterning, affects the concentration profile of molecules within the sinusoid.

The representation of all sinusoids in the liver by a single linear “pipe” is a gross, though often used, simplification of the complexity of blood flow in the liver. In the future this blood flow module will be extended and refined to better represent the complex structure of the liver microvasculature. Our modular approach will allow us to refine the sinusoid model without needing to rework the subcellular or whole-body modules.

**Model parameterization and sensitivity analysis:** We applied the multiscale framework of xenobiotics to modeling of APAP ADME at pharmacological doses and validated the model against experiment data for oral dosing in humans. Our initial parameter set was based on literature values and estimation. Refinement of the parameter set was achieved through large-scale screening of parameter values and fitting of the simulated ADME profiles with human data. We chose normal distributions on a logarithmic scale to perturb and explore parameter changes over several orders of magnitudes.

The sensitivity analyses revealed that a significant portion of the parameters (40%) have relatively little affect on the predicted ADME profiles. Interestingly, similar—though not identical—sensitivity patterns were observed for parameter sets characteristic of compounds with ADME profiles significantly different than APAP’s. This suggests that the parameter sensitivity pattern might be relatively independent of the exact xenobiotic being modeled. If we assume that these different ADME profiles correspond to models of other compounds, and all parameter sets coming out of a parameter screening procedure would map to a compound or group of compounds, then this suggests that the parameter sensitivity is an intrinsic property of the model, largely independent of the compound being studied. This observation would be quite useful since it reduces the parameter space.

**Population variability of model responses:** Using the assumption that a population of individuals can be represented as a set of parameter sets, in which each parameter is perturbed by sampling from a normal distribution with a 25% standard deviation, yields a population model that is consistent with the range of variability seen in typical ADME studies in humans. In addition, this approach provides insight into idiosyncratic responses that arise in rare individuals that are within the normal range for all parameters but interactions among the entire set of parameters gives rise to unexpected (idiosyncratic) behavior.

**Advantages of using standard modeling modalities:** Using standard modeling languages and tools to create models of biological processes has several critical advantages over *ad* *hoc* and “one-of” coding approaches. Use of standard tools reduces the need for verification, particularly between the mathematical model and the computational model, since many aspects of the tool can be verified independently, leaving only the need to verify the implementation of the characteristic parts of a model. Our use of CC3D is an example of using available software to model a particular scale that leverages a significant software development effort. The SBML models, both the PBPK and subcellular metabolic models, have several additional features that differentiate models in SBML from *ad* *hoc* modeling approaches. These features include the ability to include biological *annotations* directly in the SBML model (thus retaining a strong linkage between biological, mathematical and computational models), the ability to *define and validate units* directly in the model, the ease with which the model can be *understood and reused* by others, the ability to make the model *accessible* to others via a web search or in central database.

**Limitations and future extensions:** Currently, our representation of the liver as a single straight sinusoidal pipe representative of all sinusoids is obviously an oversimplification. This simplification results in the loss of the lobule sinusoidal network information and ignores the lobular distribution of blood flow velocity, which presumably is a contributing factor to zonal xenobiotic uptake and metabolism. We are exploring more complex lobule models ranging from using a set of sinusoids with various lengths and blood velocities to models consisting of much more complex sinusoidal anastomotic networks based on detailed histological examination of liver tissue.

We are currently extending the sub-cellular metabolic model to include necrosis caused by APAP overdose based on existing models [31, 32]. Once necrosis is included in the model, we can add the release of serum markers of necrosis (*e*.*g*., clinical measurements), such as alanine aminotransferase (ALT) and aspartate aminotransferase (AST), into all three modeling scales to couple necrosis to standard clinical markers of liver function. Our approach of separating the model into three scales will greatly facilitate refining, validating and extending the model at all scales leading to a defensible model for APAP in particular, and a reusable framework for xenobiotics in general.

## Supporting Information

S1 File Supporting Information. Supplemental information on the model development pathway, computational details, additional model parameters and output analysis.
(PDF)

S2 File Source Code. The CC3D, and SBML model files are included in this zip file.
(ZIP)

S3 File Analysis Script. Python code for generating job files for parameter scanning and output analysis.
(ZIP)

S1 Video APAP_pulse_10sec. Video file of the standalone multi-cell lobule simulation in CC3D.
(MP4)

S2 Video Complete_8hr. Video file of the complete eight hour multi-scale simulation.
(AVI)
