# Supplementary material for: A Liver-Centric Multiscale Modeling Framework for Xenobiotics
Source: PLoS One. 2016 Sep 16;11(9):e0162428. doi: 10.1371/journal.pone.0162428 (PMC5026379; doi:10.1371/journal.pone.0162428)
Supplement: S1 File — Supplemental information on the model development pathway, computational details, additional model parameters and output analysis. (PDF) [file pone.0162428.s001.pdf]

Supplementary Materials for "A Liver-centric Multiscale Modeling Framework for Xenobiotics"

James P. Sluka, Xiao Fu, Maciej Swat, Julio M. Belmonte, Alin Cosmanescu, Sherry G. Clendenon, John Wambaugh†, and James A. Glazier

Biocomplexity Institute, Indiana University
and
†US Environmental Protection Agency

March 10, 2016

Contents

1 Biological Components and Processes 4

2 Model Assumptions 6
2.1 Simplified Hepatic Lobule . . . 6
2.2 Diffusion and Transport of Acetaminophen . . . 6
2.3 Protein Binding of APAP and Metabolites . . . 6
2.4 Subcellular Metabolic Reactions . . . 6

3 Computational Methods 7
3.1 Physiologically Based Pharmacokinetic Model (PBPK) . . . 7
3.1.1 Allometric Scaling of the PBPK model's Compartment Volumes and Blood Flow Rates 7
3.1.2 PBPK ODEs . . . 7
3.2 Intercellular Molecular Transfer Model . . . 10
3.2.1 Intercellular Molecular Transfer Rate Equations ODEs . . . 10
3.3 Subcellular Reaction Kinetics Model . . . 11
3.3.1 Subcellular Reaction Kinetics ODEs . . . 11
3.4 Cellular Potts Model in Compucell3D . . . 11
3.4.1 Cell Contact, Volume Constraint . . . 12
3.4.2 External Potential . . . 12
3.4.3 Fields and Flux . . . 12
3.5 Simulation Parameters and Initial Configuration . . . 13
3.5.1 Geometrical Parameters . . . 13
3.5.2 Temporal Parameters . . . 13
3.5.3 Initial Configuration and Condition . . . 13

4 Parameter Scans and Population Simulations 13
4.1 Parameter Searching . . . 13
4.2 Population Variability . . . 14

5 Simulation Code Files 14
5.1 Subcellular reaction kinetics module . . . 14
5.2 Physiologically based pharmacokinetics module . . . 14
5.3 Multi-cell flow and transport module . . . 14
5.4 Parameter scanning scripts . . . 15

6 Model Parameters 15
6.1 Named Model Parameter Sets . . . 16

7 Model Outputs 18
7.1 Model Reproducibility . . . 18
7.2 Sensitivity of model output to model parameters . . . 18

8 Human *In Vivo* Reference Data Set 22

9 Simulation Animations 23
9.1 CC3D Standalone Simulation Animation . . . 23
9.2 Complete Eight Hour Simulation Animation . . . 23

List of Figures

1 Screen shot of the standalone multi-cell lobule simulation in CompuCell3D. The full animation is in the Supplemental file: APAP_pulse_10sec.avi. . . . 23

2 Screen shot of the complete multi-scale eight hour simulation. The full animation is in the Supplemental file: Complete_8hr.avi. 23

List of Tables

1 Model Objects and Processes 4
2 Allometric Scaling Factors 8
3 Derived ODEs for the Whole-Body PBPK Model† 9
4 Derived ODEs for the Intercellular Molecular Transfer Model of APAP 10
5 Derived ODEs for the Intercellular Molecular Transfer Model of APAPG 10
6 Derived ODEs for the Intercellular Molecular Transfer Model of APAPS 10
7 Derived ODEs for the Subcellular Reaction Kinetics Model† 11
8 CC3D Multi-cell Lobule Model Energy and Volume Parameters† 15
9 CC3D Multi-cell Lobule Model Spatiotemporal Parameters 16
10 Named Model Parameter Sets 17
11 Model Outputs for Named Parameter Sets 18
12 Stochasticity Test Results 19
13 Sensitivity of Model Output RMSEsum to Parameters 19
14 Average Sensitivity of Model Outputs to Parameters 21
15 Digitized‡ data points from Figure 1 of Critchley *et al.*† 22

1 Biological Components and Processes

A list of the biological components and behaviors (processes), along with ontological references, are given in Table 1.

Table 1: Model Objects and Processes

**Tissue Compartments**	**Properties and Behaviors [Ontology Reference: class name and URI]**
Art	Volume of the arterial blood. Note that this value is modified by allometric scaling in an initial assignment or rule equation. [Arterial Blood http://identifiers.org/fma/FMA:83066]
Gut	Perfusable volume of the Gut (small intestine). The compartment volume is allometrically scaled and is assigned in an initialAssignment. [Intestine http://identifiers.org/fma/FMA:7199]
GutLumen	Lumen of the Gut. [Lumen of gut http://identifiers.org/fma/FMA:45677]
Lung	Perfusable volume of the Lungs. The compartment volume is allometrically scaled and is assigned in an initialAssignment. [Lung http://identifiers.org/fma/FMA:7195]
Ven	Volume of the venous blood. The compartment volume is allometrically scaled and is assigned in an initialAssignment. [Venous blood http://identifiers.org/fma/FMA:83067]
Rest	Perfusable volume of the Rest of the body, that is, all tissues and blood volumes not explicitly described by other compartments. The compartment volume is allometrically scaled and is assigned in an initialAssignment. [Body http://identifiers.org/fma/FMA:20394]
Liver	Perfusable volume of the Liver. The compartment volume is allometrically scaled and is assigned in an initialAssignment. [Liver http://identifiers.org/fma/FMA:7197]
Kidney	Perfusable volume of the Kidney. The compartment volume is allometrically scaled and is assigned in an initialAssignment. [Kidney http://identifiers.org/fma/FMA:7203]
KidneyTubules	Kidney (renal) Tubules (lumen). This, like the gut lumen, is treated as a volumeless compartment. [Renal tubule http://identifiers.org/fma/FMA:15627]

**Generalized Cells**	**Properties and Behaviors [Ontology Reference]**
Blood Source Cell (BSC)	Source cells divide to form Red blood cells and serum portions at the periportal end of sinusoid. The source cells are constrained to the periportal end of the simulated sinusoid.
Red Blood Cell (RBC)	RBCs are created at the periportal end and destroyed at the pericentral end of the simulated sinusoid. RBCs are forced to move from periportal to perivenous end of the sinusoid by both the expansion of periportal blood component cells and by an applied external force directed towards the central vein. RBCs carry load of APAP (intitalized based on the input blood concentration when the RBC is created) during transit. This load diffuses into / out of the cell and exchanges with neighboring model components. [Erythrocyte http://identifiers.org/cl/CL:0000232]

Serum Portions (SP)	SPs are portions of blood serum modeled as generalized cells. SPs are created at the periportal end and destroyed at the pericentral end of the simulated sinusoid. SPs are forced to move from periportal to perivenous end of the sinusoid by both the expansion of periportal blood component cells and by an applied external force directed towards the central vein. SPs carry load of APAP (initialized based on the input blood concentration when the SP is created) during transit. This load diffuses into / out of the cell and exchanges with neighboring model components. SPs also carry a load of APAP Phase II which also diffuses into/out of the SP and exchanges with neighboring model components metabolites during transit. [Plasma http://identifiers.org/fma/FMA:62970]
Hepatocytes (HEP)	Hepatocytes are frozen (not allowed to move), and in contact with the local blood flow components. Hepatocytes each contain a sub-cellular reaction kinetic model of APAP Phase I and Phase II metabolism as well as reactions for GSH synthesis and NAPQI's reaction with GSH, the product of which does not leave the hepatocyte. APAP and Phase II metabolites diffuse into / out of hepatocytes and neighboring model components. [Hepatocyte http://identifiers.org/cl/CL:0000182]

**Chemicals**	**Properties and Behaviors [Ontology Reference]**
Acetaminophen (APAP)	APAP exists in HEP, RBC, and SP generalized cells. APAP passively diffuses across formal cell boundaries between RBC, SP, HEP giving nine passive transfer processes; $HEP \leftrightarrow HEP$; $RBC \leftrightarrow RBC$; $SP \leftrightarrow SP$; $HEP \leftrightarrow RBC$; $HEP \leftrightarrow SP$; $RBC \leftrightarrow SP$. In addition, APAP is actively transported across the HEP plasma membrane and this one active transport route ($SP \rightarrow HEP$) is modeled. Both active and passive transport is modeled only between cells (and pseudo cell SP) that are in contact. APAP undergoes both Phase I and Phase II metabolic processes and a total of three metabolic reactions; $APAP \rightarrow APAPG$; $APAP \rightarrow APAPS$; $APAP \rightarrow NAPQI$ are modeled. Each simulated HEP has its own copy of the metabolic network, working on its own pool of APAP and producing its own pool of metabolites. [Paracetemol http://identifiers.org/chebi/CHEBI:46195]
Acetaminophen Glucuronide (APAPG)	APAPG exists in HEP and SP but not in RBC, and is created (from APAP) in HEP. APAPG is actively exported across HEP plasma membranes into blood (RBC and SP) modeled by one active transport ODE; $HEP \rightarrow SP$. APAPG is formed via Phase II metabolism of APAP represented as one irreversible first order ODE. [4-Aacetamidophenyl beta-D-glucopyranosiduronic acid http://identifiers.org/chebi/CHEBI:32636]
Acetaminophen Sulfate (APAPS)	APAP exists in HEP and SP but not in RBC), and is created (from APAP) in HEP. APAPS is actively exported across HEP plasma membranes into blood (RBC and SP) modeled by one active transport ODE; $HEP \rightarrow SP$. APAPS is formed via Phase II metabolism of APAP represented as one irreversible first order ODE. [Paracetamol Sulfate http://identifiers.org/chebi/CHEBI:32635]
N-Acetyl-p-benzoquinone Imine (NAPQI)	NAPQI exists in only HEP. NAPQI is formed via Phase I metabolism of APAP, and 1 metabolic reaction is modeled. [N-Acetyl paraquinone imine http://identifiers.org/chebi/CHEBI:29132]
Glutathione (GSH)	GSH exists in only HEP and is formed in a first order ODE with a maximum concentration limitation of 10mM. GSH form conjugates with NAPQI in a second order reaction ($NAPQI + GSH \rightarrow NAPQIGSH$). GSH doe not leave the HEP it was formed in. [gamma-L-Glutamyl-L-Cysteinyl-Glycine http://identifiers.org/chebi/CHEBI:16856]

NAPQI-GSH conjugate (NAPQIGSH)	NAPQIGSH exists in only HEP. NAPQIGSH is formed via conjugation of GSH with NAPQI in a second order ODE. NAPQIGSH doe not leave the HEP it was formed in. [Acetaminophen glutathione conjugate http://identifiers.org/chebi/CHEBI:32639]

2 Model Assumptions

2.1 Simplified Hepatic Lobule

The liver is composed of millions of hepatic lobules. Liver lobules are "plumbed" in parallel and a portion of blood, while transiting the liver once, passes through exactly one lobule. Each hepatic lobule consists of various cell types including hepatocytes, Kupffer cells, stellate cells and endothelial cells and each of these contribute to normal liver function. In our simplified tissue model we focus on the liver's parenchymal cells (hepatocytes) and the two main components of blood (red blood cells and blood plasma). In addition, we have significantly simplified the sinusoidal network of the lobule by assuming a two dimensional, tubular structure lined with hepatocytes. Thus, we only modeled a small section of vascular-tissue volume and extend the result to whole liver by simple multiplication. Coupling of the Hepatic Lobule model to the whole-body PBPK model is done via the *concentration* of APAP (and metabolites) in the blood as the blood moves from the higher scale (PBPK) to the lower scale (Lobule) and back again. Since concentration is an *intensive* parameter it is scale insensitive.

2.2 Diffusion and Transport of Acetaminophen

In the lobule model we modeled the diffusion and transport using a coarse-grained PDE method. The cells (and pseudo-cell serum portions) are the coarse-graining scale and diffusion and transport is modeled only at that scale. Individual cells are treated as "well stirred" and diffusion within a cell is not explicitly modeled. In the whole-body PBPK model the individual tissue compartments are treated as "well stirred" compartments.

2.3 Protein Binding of APAP and Metabolites

We include protein binding of APAP in blood serum characterized by the **Fup** in the PBPK module. In our model, APAP-*Fup* applies only to transport between blood serum portions and hepatocytes and between blood serum portions and the kideny tubules. We neglect protein binding of APAP within RBCs and hepatocytes. We also assumed that the two APAP metabolites (APAPG and APAPS) do not bind to serum proteins and are not taken up by RBCs.

2.4 Subcellular Metabolic Reactions

We include representative common Phase I and Phase II metabolic reactions. For APAP these reactions represent the vast majority of the *in vivo* metabolism. Many other xenobiotics undergo similar reactions and the sub-cellular metabolic model is at least a starting point for modeling a wide range of chemical entities. The single Phase I reaction modeled represent Cytochrome P450 oxidation of APAP by a pool of enzymes (e.g., Cyp2E1 and Cyp1A2) to give NAPQI. NAPQI reacts rapidly with cellular nucleophiles such as glutathione. The high concentration of cellular glutathione in hepatocytes is generally sufficient to scavenge the NAPQI formed by therapeutic doses of APAP. Phase II metabolism of APAP is known to be quite extensive with both sulfate and glucuronide conjugates being formed. We model these two separately as first order reactions between APAP and unlimited pools of sulfate and glucuronic acid precursors. We are aware that APAP overdose-induced liver necrosis is likely to involve other processes, as well as saturation of the the processes we included, leading to tissue death. However, for the study of *therapeutic* dose of APAP, we assumed that necrosis-related pathways could be omitted.

3 Computational Methods

3.1 Physiologically Based Pharmacokinetic Model (PBPK)

The whole-body PBPK model is encoded in standard System Biology Markup Language (SBML) format (http://sbml.org/) for representing sets of couple Ordinary Differential Equations (ODEs). This SBML model can be run by itself using standard SBML tools. See http://sbml.org/SBML_Software_Guide/ for a listing of SBML tools for building and running SBML models. When this model is run as a sub-model of the multiscale model we use the LibRoadRunner package. LibRoadRunner is a cross-platform, open-source, high performance C++ library for running SBML-compliant models. LibRoadRunner's Python API allows the CC3D Python script to load the SBML models (including independent replicates of the same model coupled to each simulated hepatocyte), adjust parameters, time step the SBML models and set and retrieve variables. For details of LibRoadRunner see Sauro *et al*. 2013: http://biorxiv.org/content/early/2013/12/12/001230, the software is available at http://sourceforge.net/projects/libroadrunner/files/libroadrunner-1.2.1/ .

The parameters of the PBPK model can be divided into two sets: (1) Parameters that are only dependent on the individual (species, gender, body weight...) being simulated and independent of the compound and (2) Parameters that are only dependent on the compound but independent of the individual.

3.1.1 Allometric Scaling of the PBPK model's Compartment Volumes and Blood Flow Rates

Table 2 lists the allometric scaling factors used to calculate the tissue compartment volumes and volumetric blood flow rates. The scaling equation uses the ratio of the body weight (BW) (in Kg) to a reference body weight (BW_{ref}) raised to the 3/4 power for parameters such as cardiac output and Qgfr;

$$P_i = (P_{i,ref})(BW/BW_{ref})^{3/4} \tag{1}$$

Where $P_{i,ref}$ is a standard value at the standard BW. For parameters that don't scale as the 3/4 power, such as the total volume of blood, the scaling equation is;

$$P_i = (P_{i,ref})(BW/BW_{ref}) \tag{2}$$

The scaling factors and fraction values used were for humans and are given in Table 2.

The PBPK compartments are of three types; "Circulatory" (arterial and venous compartments), "Organ" (liver, kidney, ...) and "Lumen" (gut lumen, kidney lumen). The transfer equations for movement of compound are generally based on the compartment type and developed based on the transfer out of a compartment. See the main paper for the complete set of transfer ODEs.

3.1.2 PBPK ODEs

The complete set of ODEs for the PBPK model are given in Table 3. In addition, the ODEs can be extracted from the SBML model file or exported from the SBML model file into R, Matlab, *etc.* code using standard SBML tools (*cf.* http://sbml.org/Software/SBMLToolbox).

Table 2: Allometric Scaling Factors

**Parameter**	**Factor** (P_{ref})	**Type**†	**Value**‡	**Description**
BW_{ref}		Kg	70	Reference body weight
**Blood flow rates (Q's) in Liter/hour.**				
QArt	15.0	per $Kg^{3/4}$	363	Arterial blood flow (cardiac output)
QGut	0.2050	F of QArt	74.4	Gut blood flow
QLiver	0.0535	F of QArt	19.4	Liver arterial blood flow. Total Liver blood flow = QLiver + QGut
QKidney	0.2214	F of QArt	80.4	Kidney blood flow
QGfr	0.0390	per $Kg^{3/4}$	0.944	Glomerular (kidney) filtration rate
QRest	=QArt - QGut - QLiver - QKidney		189	Rest (of body) blood flow
**Compartment perfusable volumes (V's) in Liters.**				
VTotal	1.7355	per $Kg^{3/4}$	42.0	Total perfusable volume of the body.
VArt	0.0357	F of VTotal	1.50	Arterial compartment; a blood only compartment.
VGut	0.0263	F of VTotal	1.10	Gut compartment; a blood + tissue compartment.
VKidney	0.0070	F of VTotal	0.294	Kidney compartment; a blood + tissue compartment.
VLiver	0.0408	F of VTotal	1.71	Liver compartment; a blood + tissue compartment.
VLung	0.0121	F of VTotal	0.508	Lung compartment; a blood + tissue compartment.
VVen	0.0812	F of VTotal	3.41	Venous compartment; a blood only compartment.

† "F" fraction.
‡ Calculated value for a 70Kg human male.

Table 3: Derived ODEs for the Whole-Body PBPK Model†

$$\frac{\mathrm{d}}{\mathrm{d}t}\mathrm{CArt} = \underbrace{\frac{\mathtt{QCardiac}}{\mathrm{vol}\,(\mathtt{VLung})}\cdot\mathtt{CLung}}_{v_3} - \underbrace{\frac{\mathtt{QGut}}{\mathrm{vol}\,(\mathtt{VArt})}\cdot\mathtt{CArt}}_{v_1} - \underbrace{\mathtt{QRest}\cdot\frac{\mathtt{CArt}}{\mathrm{vol}\,(\mathtt{VArt})}}_{v_5} - \underbrace{\frac{\mathtt{QLiver}}{\mathrm{vol}\,(\mathtt{VArt})}\cdot\mathtt{CArt}}_{v_7}$$

$$= -\underbrace{\frac{\mathtt{QKidney}}{\mathrm{vol}\,(\mathtt{VArt})}\cdot\mathtt{CArt}}_{v_{11}}$$

$$\frac{\mathrm{d}}{\mathrm{d}t}\mathrm{CGut} = \underbrace{\frac{\mathtt{QGut}}{\mathrm{vol}\,(\mathtt{VArt})}\cdot\mathtt{CArt}}_{v_1} + \underbrace{\mathtt{kGutabs}\cdot\mathtt{AGutlumen}}_{v_2} - \underbrace{\frac{\mathtt{QGut}}{\mathrm{vol}\,(\mathtt{VGut})}\cdot\mathtt{CGut}}_{v_9}$$

$$\frac{\mathrm{d}}{\mathrm{d}t}\mathrm{AGutlumen} = -\underbrace{\mathtt{kGutabs}\cdot\mathtt{AGutlumen}}_{v_2}$$

$$\frac{\mathrm{d}}{\mathrm{d}t}\mathrm{CLung} = \underbrace{\frac{\mathtt{QCardiac}}{\mathrm{vol}\,(\mathtt{VVen})}\cdot\mathtt{CVen}}_{v_4} - \underbrace{\frac{\mathtt{QCardiac}}{\mathrm{vol}\,(\mathtt{VLung})}\cdot\mathtt{CLung}}_{v_3}$$

$$\frac{\mathrm{d}}{\mathrm{d}t}\mathrm{CVen} = \underbrace{\frac{\frac{\mathtt{QRest}}{\mathrm{vol}(\mathtt{VRest})}\cdot\mathtt{CRest}\cdot\mathtt{Ratioblood2plasma}}{\mathtt{KRest2plasma}\cdot\mathtt{Fraction\_unbound\_plasma}}}_{v_6} + \underbrace{\frac{\frac{\mathtt{QLiver+QGut}}{\mathrm{vol}(\mathtt{VLiver})}\cdot\mathtt{CLiver}\cdot\mathtt{Ratioblood2plasma}}{\mathtt{Kliver2plasma}\cdot\mathtt{Fraction\_unbound\_plasma}}}_{v_{10}}$$

$$= +\underbrace{\frac{\frac{\mathtt{QKidney}}{\mathrm{vol}(\mathtt{VKidney})}\cdot\mathtt{CKidney}\cdot\mathtt{Ratioblood2plasma}}{\mathtt{Kkidney2plasma}\cdot\mathtt{Fraction\_unbound\_plasma}}}_{v_{13}} - \underbrace{\frac{\mathtt{QCardiac}}{\mathrm{vol}\,(\mathtt{VVen})}\cdot\mathtt{CVen}}_{v_4}$$

$$\frac{\mathrm{d}}{\mathrm{d}t}\mathrm{CRest} = \underbrace{\mathtt{QRest}\cdot\frac{\mathtt{CArt}}{\mathrm{vol}\,(\mathtt{VArt})}}_{v_5} - \underbrace{\frac{\frac{\mathtt{QRest}}{\mathrm{vol}(\mathtt{VRest})}\cdot\mathtt{CRest}\cdot\mathtt{Ratioblood2plasma}}{\mathtt{KRest2plasma}\cdot\mathtt{Fraction\_unbound\_plasma}}}_{v_6}$$

$$\frac{\mathrm{d}}{\mathrm{d}t}\mathrm{CLiver} = \underbrace{\frac{\mathtt{QLiver}}{\mathrm{vol}\,(\mathtt{VArt})}\cdot\mathtt{CArt}}_{v_7} + \underbrace{\frac{\mathtt{QGut}}{\mathrm{vol}\,(\mathtt{VGut})}\cdot\mathtt{CGut}}_{v_9} - \underbrace{\frac{\mathtt{CLmetabolism}\cdot\mathtt{CLiver}}{\mathtt{Kliver2plasma}\cdot\mathtt{Fraction\_unbound\_plasma}}}_{v_8}$$

$$= -\underbrace{\frac{\frac{\mathtt{QLiver+QGut}}{\mathrm{vol}(\mathtt{VLiver})}\cdot\mathtt{CLiver}\cdot\mathtt{Ratioblood2plasma}}{\mathtt{Kliver2plasma}\cdot\mathtt{Fraction\_unbound\_plasma}}}_{v_{10}}$$

$$\frac{\mathrm{d}}{\mathrm{d}t}\mathrm{CMetabolized} = \underbrace{\frac{\mathtt{CLmetabolism}\cdot\mathtt{CLiver}}{\mathtt{Kliver2plasma}\cdot\mathtt{Fraction\_unbound\_plasma}}}_{v_8}$$

$$\frac{\mathrm{d}}{\mathrm{d}t}\mathrm{CKidney} = \underbrace{\frac{\mathtt{QKidney}}{\mathrm{vol}\,(\mathtt{VArt})}\cdot\mathtt{CArt}}_{v_{11}} - \underbrace{\frac{\frac{\mathtt{Qgfr}}{\mathrm{vol}(\mathtt{VKidney})}\cdot\mathtt{CKidney}}{\mathtt{Kkidney2plasma}}}_{v_{12}} - \underbrace{\frac{\frac{\mathtt{QKidney}}{\mathrm{vol}(\mathtt{VKidney})}\cdot\mathtt{CKidney}\cdot\mathtt{Ratioblood2plasma}}{\mathtt{Kkidney2plasma}\cdot\mathtt{Fraction\_unbound\_plasma}}}_{v_{13}}$$

$$\frac{\mathrm{d}}{\mathrm{d}t}\mathrm{CTubules} = \underbrace{\frac{\frac{\mathtt{Qgfr}}{\mathrm{vol}(\mathtt{VKidney})}\cdot\mathtt{CKidney}}{\mathtt{Kkidney2plasma}}}_{v_{12}}$$

†Species quantities are in mass units.

3.2 Intercellular Molecular Transfer Model

The molecular transfer model is coded and run in CC3D.

3.2.1 Intercellular Molecular Transfer Rate Equations ODEs

The complete set of ODEs for the Molecular Transfer model for APAP, APAPG and APAPS are given in Table 4,5,6. Superscripts refer to the unique identifiers for each cell. The set of cells C^j are the contact neighbors of the cell C^i. Subscripts identify the cell type: H = hepatocyte, S = serum portion, R = red blood cell. $pbpk\_Fup$ = Fraction unbound to protein, n^i = number of contact neighbors.

Table 4: Derived ODEs for the Intercellular Molecular Transfer Model of APAP

$$\frac{d}{dt}C^i_H = \sum_j^{\text{neighbors of i}}[\delta(\sigma_j,H)\cdot(\texttt{cc3d\_k\_PD\_H2H}\cdot C^j_H/n^j - \texttt{cc3d\_k\_PD\_H2H}\cdot C^i_H/n^i)+ \delta(\sigma_j,R)\cdot(\texttt{cc3d\_k\_PD\_R2H}\cdot C^j_R/n^j - \texttt{cc3d\_k\_PD\_H2R}\cdot C^i_H/n^i)+ \delta(\sigma_j,S)\cdot(\texttt{cc3d\_k\_PD\_S2H}\cdot C^j_S/n^j - \texttt{cc3d\_k\_PD\_H2S}\cdot C^i_H/n^i + \frac{\texttt{cc3d\_Vmax\_AT\_APAP}\cdot C^j_S}{C^j_S+\texttt{cc3d\_Km\_AT\_APAP}}\cdot \texttt{pbpk\_Fup})]$$

$$\frac{d}{dt}C^i_R = \sum_j^{\text{neighbors of i}}[\delta(\sigma_j,R)\cdot(\texttt{cc3d\_k\_PD\_R2R}\cdot C^j_R/n^j - \texttt{cc3d\_k\_PD\_R2R}\cdot C^i_R/n^i)+ \delta(\sigma_j,S)\cdot(\texttt{cc3d\_k\_PD\_S2R}\cdot C^j_S/n^j - \texttt{cc3d\_k\_PD\_R2S}\cdot C^i_R/n^i)+ \delta(\sigma_j,H)\cdot(\texttt{cc3d\_k\_PD\_H2R}\cdot C^j_H/n^j - \texttt{cc3d\_k\_PD\_R2H}\cdot C^i_R/n^i]$$

$$\frac{d}{dt}C^i_S = \sum_j^{\text{neighbors of i}}[\delta(\sigma_j,S)\cdot(\texttt{cc3d\_k\_PD\_S2S}\cdot C^j_S/n^j - \texttt{cc3d\_k\_PD\_S2S}\cdot C^i_S/n^i)+ \delta(\sigma_j,H)\cdot(\texttt{cc3d\_k\_PD\_H2S}\cdot C^j_H/n^j - \texttt{cc3d\_k\_PD\_H2S}\cdot C^i_S/n^i - \frac{\texttt{cc3d\_Vmax\_AT\_APAP}\cdot C^i_S}{C^i_S+\texttt{cc3d\_Km\_AT\_APAP}}\cdot \texttt{pbpk\_Fup})+ \delta(\sigma_j,R)\cdot(\texttt{cc3d\_k\_PD\_R2S}\cdot C^j_R/n^j - \texttt{cc3d\_k\_PD\_S2R}\cdot C^i_S/n^i)]$$

Table 5: Derived ODEs for the Intercellular Molecular Transfer Model of APAPG

$$\frac{d}{dt}C^i_H = \sum_j^{\text{neighbors of i}}[\delta(\sigma_j,R)\cdot(-\texttt{cc3d\_k\_AT\_APAPG}\cdot C^i_H)+ \delta(\sigma_j,S)\cdot(-\texttt{cc3d\_k\_AT\_APAPG}\cdot C^i_H)]$$

$$\frac{d}{dt}C^i_R = \sum_j^{\text{neighbors of i}}[\delta(\sigma_j,H)\cdot(\texttt{cc3d\_k\_AT\_APAPG}\cdot C^j_H)]$$

$$\frac{d}{dt}C^i_S = \sum_j^{\text{neighbors of i}}[\delta(\sigma_j,H)\cdot(\texttt{cc3d\_k\_AT\_APAPG}\cdot C^j_H)]$$

Table 6: Derived ODEs for the Intercellular Molecular Transfer Model of APAPS

$$\frac{d}{dt}C^i_H = \sum_j^{\text{neighbors of i}}[\delta(\sigma_j,R)\cdot(-\texttt{cc3d\_k\_AT\_APAPS}\cdot C^i_H)+ \delta(\sigma_j,S)\cdot(-\texttt{cc3d\_k\_AT\_APAPS}\cdot C^i_H)]$$

$$\frac{d}{dt}C^i_R = \sum_j^{\text{neighbors of i}}[\delta(\sigma_j,H)\cdot(\texttt{cc3d\_k\_AT\_APAPS}\cdot C^j_H)]$$

$$\frac{d}{dt}C^i_S = \sum_j^{\text{neighbors of i}}[\delta(\sigma_j,H)\cdot(\texttt{cc3d\_k\_AT\_APAPS}\cdot C^j_H)]$$

3.3 Subcellular Reaction Kinetics Model

The metabolic reaction kinetics model is coded and run in SBML in the same way as the PBPK model.

3.3.1 Subcellular Reaction Kinetics ODEs

The complete set of ODEs for the reaction kinetic model are given in Table 7. In addition, the ODEs can be extracted from the SBML model file or exported from the SBML model file into R, Matlab, *etc.* code using standard SBML tools (*cf.* http://sbml.org/Software/SBMLToolbox).

Table 7: Derived ODEs for the Subcellular Reaction Kinetics Model†

$$\frac{\mathrm{d}}{\mathrm{d}t}[\mathrm{APAP}] = -\underbrace{\frac{\mathtt{Vmax\_2E1\_APAP}\cdot[\mathtt{APAP}]}{\mathtt{Km\_2E1\_APAP}+[\mathtt{APAP}]}}_{v_1} - \underbrace{\frac{\mathtt{Vmax\_PhaseIIEnzGlu\_APAP}\cdot[\mathtt{APAP}]}{\mathtt{Km\_PhaseIIEnzGlu\_APAP}+[\mathtt{APAP}]}}_{v_4} - \underbrace{\frac{\mathtt{Vmax\_PhaseIIEnzSul\_APAP}\cdot[\mathtt{APAP}]}{\mathtt{Km\_PhaseIIEnzSul\_APAP}+[\mathtt{APAP}]}}_{v_5}$$

$$\frac{\mathrm{d}}{\mathrm{d}t}[\mathrm{NAPQI}] = \underbrace{\frac{\mathtt{Vmax\_2E1\_APAP}\cdot[\mathtt{APAP}]}{\mathtt{Km\_2E1\_APAP}+[\mathtt{APAP}]}}_{v_1} - \underbrace{\mathtt{kNapqiGsh}\cdot[\mathtt{NAPQI}]\cdot[\mathtt{GSH}]\cdot\mathrm{vol}\,(\mathtt{compartment})\cdot\mathrm{vol}\,(\mathtt{compartment})}_{v_2}$$

$$\frac{\mathrm{d}}{\mathrm{d}t}[\mathrm{GSH}] = \underbrace{\mathtt{kGsh}\cdot(\mathtt{GSHmax}-[\mathtt{GSH}])\cdot\mathrm{vol}\,(\mathtt{compartment})}_{v_3} - \underbrace{\mathtt{kNapqiGsh}\cdot[\mathtt{NAPQI}]\cdot[\mathtt{GSH}]\cdot\mathrm{vol}\,(\mathtt{compartment})\cdot\mathrm{vol}\,(\mathtt{compartment})}_{v_2}$$

$$\frac{\mathrm{d}}{\mathrm{d}t}[\mathrm{NAPQIGSH}] = \underbrace{\mathtt{kNapqiGsh}\cdot[\mathtt{NAPQI}]\cdot[\mathtt{GSH}]\cdot\mathrm{vol}\,(\mathtt{compartment})\cdot\mathrm{vol}\,(\mathtt{compartment})}_{v_2}$$

$$\frac{\mathrm{d}}{\mathrm{d}t}[\mathrm{APAPconj\_Glu}] = \underbrace{\frac{\mathtt{Vmax\_PhaseIIEnzGlu\_APAP}\cdot[\mathtt{APAP}]}{\mathtt{Km\_PhaseIIEnzGlu\_APAP}+[\mathtt{APAP}]}}_{v_4}$$

$$\frac{\mathrm{d}}{\mathrm{d}t}[\mathrm{APAPconj\_Sul}] = \underbrace{\frac{\mathtt{Vmax\_PhaseIIEnzSul\_APAP}\cdot[\mathtt{APAP}]}{\mathtt{Km\_PhaseIIEnzSul\_APAP}+[\mathtt{APAP}]}}_{v_5}$$

† Square brackets indicate concentrations.

3.4 Cellular Potts Model in Compucell3D

Cellular Potts Model (CPM) defines a generalized **cell** as a collection of *lattice sites* or *voxels* at location $\vec{l}$, acquiring common *cell index*, σ, in a **cell-lattice**. Generalized cells refer to either biological cells (hepatocytes, red blood cells) or pseudo cells (blood plasma, blood source cells). Each cell has a **cell type**

associated with it. Evolution of **cell-lattice** configuration in the system is governed by *Effective Energy*, which is constructed in this model using factors introduced below.

3.4.1 Cell Contact, Volume Constraint

The basic expression of *effective energy* (H) in CPM has terms contributed by adhesion and volume constraint:

$$H = \sum_{\substack{\vec{i},\vec{j} \\ neighbors}} J(\tau(\sigma_{\vec{i}}), \tau(\sigma_{\vec{j}}))(1 - \delta(\sigma_{\vec{i}}, \sigma_{\vec{j}})) + \sum_{\sigma} [\lambda_{vol}(\sigma)(v(\sigma) - V_t(\sigma))^2] \quad (3)$$

The summation of $J(\tau(\sigma_{\vec{i}}), \tau(\sigma_{\vec{j}}))$ calculates the *contact energy* between two neighboring cells. $J(\tau(\sigma_{\vec{i}}), \tau(\sigma_{\vec{j}}))$ represents energy per contact area between the cell $\sigma_{\vec{i}}$ with cell type $\tau(\sigma_{\vec{i}})$ located at cell lattice site $\vec{i}$ and the cell $\sigma_{\vec{j}}$ with cell type $\tau(\sigma_{\vec{j}})$ located at cell lattice site $\vec{j}$. And calculation excludes the pair of lattice sites belonging to the same cell by multiplication with a factor $(1 - \delta(\sigma_{\vec{i}}, \sigma_{\vec{j}}))$. The second summation over all generalized cells σ defines a penalty energy for cells with a volume deviated from its target volume $V_t(\sigma)$, and the strength of penalty is described by $\lambda_{vol}(\sigma)$.

3.4.2 External Potential

The external potential is incorporated by imposing a directed force on the generalized cells. It's used to achieve persistent cell movement. The additional term in the effective energy (H) caused by the external force is:

$$\Delta H_{ext} = \sum_{\vec{i}} [-\vec{\lambda}_{ext}(\sigma(\vec{i})) \cdot \vec{c}] \quad (4)$$

The summation calculates the contribution of external force to the total effective energy in the system. The term $-\vec{\lambda}_{ext}(\sigma(\vec{i}))$ is the force vector for cell $\sigma(\vec{i})$ at cell lattice site $\vec{i}$. $\vec{c}$ is the direction of the pixel copy attempt.

3.4.3 Fields and Flux

We used a course-grained method to model the diffusion processes in the multi-cell sinusoid model. Chemicals are considered uniformly distributed within each generalized cell. Concentration gradients only exists across the interface between different instances of generalized cells. For passive transport, flux is driven by the concentration gradient across the common surface of two cells. For active transport, flux is dependent on the concentration within the source cell.

APAP APAP exists in all generalized cells except BSC (blood source cells). Transport flux of APAP falls into two categories; Passive transport of APAP is modeled between RBC, SP and HEP. Active transport of APAP is modeled for the uptake by HEP ($SP \rightarrow HEP$). Passive transport processes is modeled as a linear ODE, active transport is modeled assuming Michaelis-Menton kinetics. Reaction of APAP to form Phase I and Phase II metabolites are modeled, within each HEP, as Michaelis-Menton kinetics.

APAPG and APAPS We assume that APAPG and APAPS exist only in serum portions (SPs) and this is enforced in the PBPK model by the blood-to-plasma partition coefficient. APAPG and APAPS are assumed to not be taken up by other tissues except the kidney.

GSH, NAPQI and NAPQIGSH We assume that GSH, NAPQI and NAPQIGSH exist only in HEPs and do not transfer between HEPs. GSH participates in a conjugation reaction with NAPQI (to form NAPQIGSH), and GSH is replenished by a relatively slow reaction from unlimited, and unspecified, precursors.

3.5 Simulation Parameters and Initial Configuration

In our multiscale model, simulation parameters span several scales. At whole-body level, parameters include blood volumetric flow rate $\boldsymbol{Q}$s, organ perfusable volume $\boldsymbol{V}$s, body weight ***bw***, hematocrit ***hemat***, partition coefficient $\boldsymbol{K}$s and $\boldsymbol{R}$s; At sub-cellular level, parameters are mainly reaction rate coefficients ***Vmax***s, ***Km***s and ***k***s.

At the multi-cell level, parameters can be divided into chemical-dependent and chemical-independent sub-groups. Chemical-independent parameters include sinusoid geometry, blood flow rate, blood composition and cellular qualities such as volume and adhesion energies between the various cell and pseudo-cell types. Chemical-dependent parameters include diffusion and transport rate constants (or partition coefficients). CC3D parameters include adhesion energy terms $J(\tau(\sigma_{\vec{i}}), \tau(\sigma_{\vec{j}}))$, volumetric constraint strength λ_{vol}, external potential strength λ_{ext}. Transport parameters are mainly passive transport rate coefficients ***k_PD***s, active transport Michaelis-Menton constants ***Vmax***s, ***Km***s. These parameters were estimated by fitting to *invivo* human data for therapeutic doses of APAP. Geometrical parameters are determined according to the simplified model architecture (see below).

3.5.1 Geometrical Parameters

Our model focuses on a liver lobule section including one sinusoid vessel and two rows of hepatocytes in a 2D plane. The whole lobule was treated as a collection of repeats of this minimal structure. The entire liver is treated as a collection of lobules. The model has a 2D interface but a pseudo-3D setup with thickness allowing estimation of 3D diffusion calculations (*e.g.*, subcellular RK input). In the multi-cell model, the relationship between model pixels and simulated distances is given by the scaling factor *px2mm*. We assume that each sinusoid vessel has a length of $200\mu m$ (approximately 10 hepatocytes), connecting a portal triad to a central vein, and a cross sectional area of $20\mu m \times 4\mu m$. We assume the hepatocytes are cubical with edges of $20\mu m$.

3.5.2 Temporal Parameters

Simulated time is a key component of all three of the sub-models and proper synchronization of time across the sub-models is critical. We take one CC3D "Monte Carlo Step" (**MCS**) as the minimal temporal length in our model. Every MCS the multicell model is updated. In addition, the CC3D model handles the transport as an innate part of the multicell module and are integrated in CC3D python code. Every 10 MCS, the ODE-based modules are integrated and updated.

3.5.3 Initial Configuration and Condition

Each simulation starts with regularly aligned blood source cells (4 in total) and blood serum portions (4 in each stack) filling in the sinusoid vessel. Hepatocytes are located laterally in contact with the vessel walls (10 for each edge). The key input of the model is the APAP oral dose (in grams) specified in the CC3D script. The dose value is copied to the PBPK module at the start of the run.

4 Parameter Scans and Population Simulations

4.1 Parameter Searching

Parameter searching was carried out by choosing multipliers for each of the 38 parameters in the complete model. The multipliers were chosen from a Log-normal distribution with a mean of 0 and a coefficient of variation of 25% with the restrictions outlined below. The parameter assignment rules for the parameter searching modeling were:

$$P_{new} = 10^{\alpha} \cdot P_{old} \tag{5}$$

Where α is selected from a normal distribution with mean of 1 and standard deviation of 0.25. The choice of α is further constrained by;

$$\text{Constraint } C_1 : -2 < \alpha < 2 \qquad (P_{old}/1000 < P_{new} < P_{old} \cdot 1000)$$
$$\text{Constraint } C_2 : -0.1 < \alpha < 0.1 \qquad (P_{old}/1.26 < P_{new} < P_{old} \cdot 1.26)$$

The parameter scaling and constraints were applied to:

Parameters not changed : pbpk_dose, pbpk_FupG, pbpk_FupS,
Parameters obeying C_2 : pbpk_Rb2p, pbpk_Rb2pG, pbpk_Rb2pS, pbpk_hemat,
: pbpk_bw and pbpk_Fup
Parameters obeying C_1 : all other parameters

4.2 Population Variability

Population analysis was carried out by choosing multipliers for each of the 38 parameters in the complete model. The multipliers were chosen from a normal distribution with a mean of 1 and a coefficient of variation of 25% with the restrictions outlined below. We used 839 randomly generated parameter sets to represent a population of individuals. The parameter assignment rules for the population variability modeling were:

$$P_{new} = \beta \cdot P_{old} \tag{6}$$

Where β is selected from a normal distribution with mean of 1 and standard deviation of 0.25. The choice of β is further constrained by;

$$\text{Constraint } C_1 : 0.75 < \beta < 1.25$$
$$\text{Constraint } C_2 : 0.75 < \beta < 1$$

The parameter scaling and constraints were applied to:

Parameters not changed : pbpk_dose
Parameters obeying C_2 : pbpk_FupG, and pbpk_FupS
Parameters obeying C_1 : all other parameters

5 Simulation Code Files

5.1 Subcellular reaction kinetics module

Refer to file **APAP_GSH_Liver_Metab_Sul_Glu__final.xml**. Standalone simulations can be done using Jarnac within Systems Biology Workbench (`http://sbw.sourceforge.net/`) or COPASI (`http://www.copasi.org/`).

5.2 Physiologically based pharmacokinetics module

Refer to file **vLiver_PBPK_based_v4__final.xml**. Standalone simulations can be done using Jarnac or COPASI. COPASI will complain about the unit definitions, since COPASI doesn't fully support the SBML unit standards, but the model runs correctly. Since a number of the model parameters (compartment volumes and volumetric flows in particular) are assigned via SBML `IntialAssignment` statements those values will be displayed incorrectly in many user interfaces. When the model is run the correct parameters will be used.

5.3 Multi-cell flow and transport module

Refer to the sub-directory **"cc3dCode"** for reference simulation files. XML files in this directory are not annotated. See the Compucell3D website for tutorials if necessary (`http://Compucell3D.org/`).

5.4 Parameter scanning scripts

Refer to sub-directories **log_normal**, **single_parameter**, **pairwise_parameter**, and **population_variability** for scripts to generate multiple simulation files based on a particular reference simulation parameter set, and relevant submission scripts.

6 Model Parameters

Detailed lists of parameters for each of the sub-models is given in the tables that follow. Energy and volume parameters for the multi-cell lobule model in CC3D are given in Table 8 and the spatiotemporal parameters in Table 9.

Table 8: CC3D Multi-cell Lobule Model Energy and Volume Parameters†

**Name**	**Value**	**Units‡**	**Description**
$J_{HEP,RBC}$	10	$E/(pixel\ edge)$	Adhesion energy per unit contact area between a pair of cells with cell types hepatocyte–red blood cells
$J_{HEP,SP}$	5	$E/(pixel\ edge)$	Adhesion energy per unit contact area between a pair of cells with cell types hepatocyte–serum portions
$J_{RBC,RBC}$	10	$E/(pixel\ edge)$	Adhesion energy per unit contact area between a pair of cells with cell types red blood cells–red blood cells
$J_{RBC,SP}$	5	$E/(pixel\ edge)$	Adhesion energy per unit contact area between a pair of cells with cell types red blood cells–serum portions
$J_{SP,SP}$	5	$E/(pixel\ edge)$	Adhesion energy per unit contact area between a pair of cells with cell types serum portions–serum portions
$V_t(HEP)$	400	$pixel$	Target volume of hepatocytes
$V_t(RBC)$	100	$pixel$	Target volume of red blood cells
$\lambda_{vol}(RBC)$	10	$E/pixel$	Energy penalty for deviation from target volume of red blood cells
$V_t(SP)$	25	$pixel$	Target volume of serum portions
$\lambda_{vol}(SP)$	10	$E/pixel$	Energy penalty for deviation from target volume of serum portions

†Since the hepatocytes are fixed in the simulation their J and λ_{vol} parameters are irrelevant and not shown.
‡Energy units are on an arbitrary scale.

Table 9: CC3D Multi-cell Lobule Model Spatiotemporal Parameters

**Category**	**Name**	**Value**	**Units**	**Description**
Geometrical	$px2mm$	1×10^{-3}	$^{mm}/_{pixel}$	Geometrical scaling factor to convert spatial sizes in model pixels units to real units
Geometrical	$length$	200	$pixel$	Length of sinusoid vessel
Geometrical	$width$	20	$pixel$	Width of sinusoid vessel
Geometrical*	$depth_{sin}$	4	$pixel$	Depth of sinusoid vessel
Geometrical	$size_{sin}$	20	$pixel$	Edge length of square hepatocyte
Geometrical*	$depth_{hep}$	20	$pixel$	Depth of hepatocyte
Geometrical	$size_{hep}$	5	$pixel$	Size of square serum portions (initial setup)
Temporal	$mcs2sec$	1.25×10^{-3}	$^{seconds}/_{MCS}$	Conversion factor between model time units (Monty Carlo Steps, MCS) and simulated time
Temporal	δt	10	MCS	Time of integration for reaction ODEs and transport fluxes

6.1 Named Model Parameter Sets

The Table 10 includes a listing of the parameter sets (fixed points) described in the paper. The parameter sets **REFSIM** and **HMPCsim6** both give reasonably good agreement with *in vivo* human data for a 1.4g oral dose of APAP. Paramter sets **LNsim8** and **LNsim23** were purposely chosen to represent compounds with ADME characteristics significantly different from APAP.

Table 10: Named Model Parameter Sets

Parameter	Unit	REFSIM	HMPCsim6	LNsim8	LNsim23
pbpk_Fup	unitless	8.E-1	8.E-1	6.864E-1	6.795E-1
pbpk_FupG	unitless	1.	1.	1.	1.
pbpk_FupS	unitless	1.	1.	1.	1.
pbpk_Kk2p	unitless	1.	1.	2.124	9.389E-1
pbpk_Kk2pG	unitless	1.	1.	5.154	1.587
pbpk_Kk2pS	unitless	1.	1.	6.805E-1	1.511
pbpk_Kr2p	unitless	1.6	1.7	3.241	3.586
pbpk_Kr2pG	unitless	4.E-1	2.5E-1	8.326E-2	2.745E-1
pbpk_Kr2pS	unitless	2.E-1	1.E-1	1.703E-1	3.752E-1
pbpk_Qgfr	L/hr	7.14E-1	7.14E-1	6.91E-1	1.188
pbpk_QgfrG	L/hr	7.86	7.86	8.998	2.988
pbpk_QgfrS	L/hr	9.96	9.96	1.013E+1	4.486
pbpk_Rb2p	unitless	1.09	1.09	1.234	1.27
pbpk_Rb2pG	unitless	5.5E-1	5.5E-1	5.747E-1	6.86E-1
pbpk_Rb2pS	unitless	5.5E-1	5.5E-1	6.2E-1	5.495E-1
pbpk_bw	Kg	7.E+1	7.E+1	7.969E+1	7.934E+1
pbpk_dose	g	1.4			
pbpk_hemat	unitless	4.5E-1	4.5E-1	5.226E-1	4.802E-1
pbpk_kGutabs	$1/hr$	1.5	1.4	1.527	8.643E-1
cc3d_Km_AT_APAP	$mmol/L$	1.E-2	1.E-2	1.141E-2	5.175E-3
cc3d_Vmax_AT_APAP	$mmol/L/s$	1.E-2	1.E-2	3.181E-3	1.088E-2
cc3d_k_AT_APAPG	$1/s$	4.5E-4	4.5E-4	3.826E-4	4.753E-4
cc3d_k_AT_APAPS	$1/s$	1.9E-3	1.9E-3	1.144E-3	4.409E-3
cc3d_k_PD_H2H	$1/s$	1.E-3	1.E-3	1.969E-3	1.27E-3
cc3d_k_PD_H2R	$1/s$	1.E-3	1.E-3	1.17E-3	6.067E-4
cc3d_k_PD_H2S	$1/s$	1.E-3	1.E-3	2.267E-3	8.969E-4
cc3d_k_PD_R2H	$1/s$	1.E-3	1.E-3	2.758E-4	6.241E-4
cc3d_k_PD_R2R	$1/s$	1.E-3	1.E-3	2.909E-3	5.87E-4
cc3d_k_PD_R2S	$1/s$	1.E-3	1.E-3	8.486E-4	8.718E-4
cc3d_k_PD_S2H	$1/s$	1.E-3	1.E-3	1.404E-3	1.804E-3
cc3d_k_PD_S2R	$1/s$	1.2E-3	1.2E-3	1.229E-3	1.362E-3
cc3d_k_PD_S2S	$1/s$	1.E-2	1.E-2	8.786E-3	7.864E-3
sc_Km_2E1_APAP	$mmol/L$	1.29	1.29	2.003	6.382E-1
sc_Km_GLUC	$mmol/L$	1.	1.5	1.461	1.138
sc_Km_SULF	$mmol/L$	2.E-1	3.E-1	2.77E-1	1.484E-1
sc_Vmax_2E1_APAP	$mmol/L/s$	2.E-5	2.E-5	1.689E-5	1.36E-5
sc_Vmax_GLUC	$mmol/L/s$	1.E-3	9.E-4	1.736E-3	7.427E-4
sc_Vmax_SULF	$mmol/L/s$	1.75E-4	1.5E-4	2.411E-4	2.219E-4
sc_kGsh	$mmol/L/s$	1.E-4	1.E-4	5.338E-5	5.65E-5
sc_kNapqiGsh	$mmol/L/s$	1.E-1	1.E-1	1.291E-1	1.614E-1

7 Model Outputs

The complete list of model outputs for each named parameter set is given in table 11.

Table 11: Model Outputs for Named Parameter Sets (Fixed Points)

**Output**	**Unit**	**REFSIM**	**HMPCsim6**	**LNsim8**	**LNsim23**
RMSEsum	$\mu g/ml \cdot hr$	2.902	1.791	1.028E+1	1.331E+1
RMSEapap	$\mu g/ml \cdot hr$	8.675E-1	1.032	3.16	5.717
RMSEapapg	$\mu g/ml \cdot hr$	1.118	3.513E-1	5.108	1.688
RMSEapaps	$\mu g/ml \cdot hr$	9.164E-1	4.073E-1	2.008	5.907
CmaxA	$\mu g/ml$	1.601E+1	1.522E+1	1.116E+1	6.774
CmaxG	$\mu g/ml$	1.189E+1	1.085E+1	3.109	1.218E+1
CmaxS	$\mu g/ml$	5.346	4.488	1.378	1.154E+1
TmaxA	hr	1.083	1.308	1.45	1.583
TmaxG	hr	3.883	3.475	2.817	4.425
TmaxS	hr	2.967	2.425	2.992	4.708
AUCA	$\mu g/ml \cdot hr$	6.676E+1	6.755E+1	6.768E+1	3.302E+1
AUCG	$\mu g/ml \cdot hr$	7.153E+1	6.531E+1	2.057E+1	7.324E+1
AUCS	$\mu g/ml \cdot hr$	3.237E+1	2.77E+1	9.316	7.237E+1
metabRatio	unitless	9.115E+1	9.016E+1	7.971E+1	9.168E+1

7.1 Model Reproducibility

While the PBPK and sub-cellular reaction kinetic ODE models are deterministic, the multi-cell lobule module is stochastic, hence the entire model is stochastic. To examine the run-to-run variability caused by the stochasticity we ran 50 replicates of the complete model using the same set of initial conditions and the **REFSIM** parameter set. As shown in Table 12, the standard deviations for all of the model outputs are parts per billion or less, indicating no significant run-to-run uncertainty or variability caused by the stochasticity of the lobule sub-model.

† The resolution of time outputs is limited by the frequency that the multi-scale model writes data to a log file. For these 50 simulations the time resolution in the log file is approximately 5 minutes and any variation of less than that amount is not visible.

‡ See Table 11 for unit definitions.

7.2 Sensitivity of model output to model parameters

The sensitivity of the model's output to the models input is given in the following two tables. Table 13 gives the sensitivity of the single model output RMSEsum to the parameter values in the four named parameter sets. Table 14 gives the average sensitivity across *all* of the model outputs (listed in the first column in Tables 11 or 12) to the parameter values in the four named parameter sets.

Table 12: Stochasticity test results for 50 replicates of REFSIM simulation

**Model Output**	**Average**[‡]	**Standard Deviation**
RMSEsum	2.847640781	2.01989E-09
RMSEapap	1.061049366	4.3401E-10
RMSEapapg	0.962405447	1.09677E-09
RMSEapaps	0.824185969	7.46289E-10
CmaxA	15.90877634	1.81448E-09
CmaxG	11.8412282	2.00659E-09
CmaxS	5.277321332	2.27096E-09
TmaxA[†]	1.183333333	8.97196E-16
TmaxG[†]	3.725	3.58878E-15
TmaxS[†]	3	0
AUCA	64.41943234	4.71818E-09
AUCG	70.46221715	8.57752E-09
AUCS	31.59780491	5.17515E-09
metabRatio	91.38942054	1.5353E-09

Table 13: Sensitivity of Model Output RMSEsum to Parameters

**Parameter**[†]	**REFSIM**	**HMPCsim6**	**LNsim8**	**LNsim23**
pbpk_Fup	1.108	1.961	1.965E-1	3.143E-1
pbpk_FupG	1.974	2.709	1.586E-1	2.427E-1
pbpk_FupS	8.57E-1	1.084	7.002E-2	4.072E-1
pbpk_Kk2p	1.795E-3	9.548E-3	2.403E-3	2.253E-4
pbpk_Kk2pG	1.617E-3	4.317E-3	3.077E-3	1.684E-4
pbpk_Kk2pS	2.981E-4	3.573E-3	2.757E-4	3.126E-3
pbpk_Kr2p	1.068	1.626	3.37E-1	3.681E-2
pbpk_Kr2pG	1.687E-1	3.372E-1	7.812E-3	1.257E-2
pbpk_Kr2pS	1.677E-2	5.642E-2	1.116E-2	1.135E-1
pbpk_Qgfr	2.892E-2	7.843E-2	5.605E-4	1.014E-2
pbpk_QgfrG	1.296	1.413	1.231E-1	1.724E-1
pbpk_QgfrS	6.803E-1	7.221E-1	4.748E-2	2.303E-1
pbpk_Rb2p	8.513E-1	1.107	4.337E-2	3.092E-1
pbpk_Rb2pG	8.517E-1	3.812E-1	8.95E-2	2.182E-1
pbpk_Rb2pS	4.258E-1	2.286E-1	3.755E-2	2.589E-1
pbpk_bw	1.876	3.026	1.347E-1	7.43E-2
pbpk_dose				
pbpk_hemat	1.075	2.417	1.408E-1	3.469E-1
pbpk_kGutabs	6.856E-1	1.83	1.789E-1	1.88E-1
cc3d_Km_AT_APAP	1.281	1.137	1.284E-1	2.86E-1
cc3d_Vmax_AT_APAP	1.876	3.714	3.289E-1	6.015E-1
cc3d_k_AT_APAPG	3.436E-2	9.279E-2	6.417E-3	1.714E-2

cc3d_k_AT_APAPS	5.855E-3	1.46E-2	1.729E-3	1.866E-4
cc3d_k_PD_H2H	7.871E-4	1.078E-3	7.743E-5	2.892E-4
cc3d_k_PD_H2R	1.72E-1	2.352E-1	1.684E-2	3.082E-2
cc3d_k_PD_H2S	7.087E-1	7.854E-1	1.066E-1	1.506E-1
cc3d_k_PD_R2H	9.901E-4	8.036E-4	1.472E-4	1.077E-4
cc3d_k_PD_R2R	2.307E-8	4.589E-8	4.087E-10	3.276E-9
cc3d_k_PD_R2S	1.629E-4	1.7E-4	1.323E-5	4.251E-5
cc3d_k_PD_S2H	1.611E-3	1.267E-3	8.631E-4	3.13E-4
cc3d_k_PD_S2R				
cc3d_k_PD_S2S	4.846E-4	4.442E-4	3.532E-5	1.046E-4
sc_Km_2E1_APAP	2.391E-2	4.205E-2	8.052E-4	9.112E-3
sc_Km_GLUC	7.226E-1	1.681	1.144E-1	1.161E-1
sc_Km_SULF	7.532E-2	6.083E-1	1.631E-2	5.809E-2
sc_Vmax_2E1_APAP	2.428E-2	4.393E-2	7.63E-4	1.011E-2
sc_Vmax_GLUC	4.868E-1	1.956	1.144E-1	8.507E-2
sc_Vmax_SULF	2.568E-1	1.246	1.728E-2	1.049E-1
sc_kGsh	1.924E-9	2.123E-9	6.812E-10	2.675E-9
sc_kNapqiGsh	1.854E-9	4.269E-9	3.114E-10	1.593E-9

† See Tables 10 for unit definitions.

Table 14: Average Sensitivity of Model Outputs to Parameters

**Parameter**[†]	**REFSIM**	**HMPCsim6**	**LNsim8**	**LNsim23**
pbpk_Fup	6.814E-1	7.576E-1	2.446E-1	2.469E-1
pbpk_FupG	6.211E-1	1.239	1.607E-1	2.774E-1
pbpk_FupS	3.62E-1	5.051E-1	1.467E-1	2.065E-1
pbpk_Kk2p	1.023E-3	2.132E-3	1.676E-3	4.96E-4
pbpk_Kk2pG	8.434E-4	2.232E-3	1.313E-2	1.864E-2
pbpk_Kk2pS	3.778E-4	1.57E-3	3.689E-4	1.621E-3
pbpk_Kr2p	4.121E-1	4.47E-1	3.243E-1	2.775E-1
pbpk_Kr2pG	9.557E-2	1.874E-1	3.535E-2	5.533E-2
pbpk_Kr2pS	3.418E-2	5.391E-2	5.13E-2	7.962E-2
pbpk_Qgfr	3.697E-2	5.221E-2	4.945E-3	3.049E-2
pbpk_QgfrG	4.134E-1	6.792E-1	1.462E-1	1.729E-1
pbpk_QgfrS	2.982E-1	3.787E-1	1.435E-1	1.205E-1
pbpk_Rb2p	3.309E-1	3.661E-1	2.234E-1	2.523E-1
pbpk_Rb2pG	2.686E-1	2.215E-1	7.872E-2	2.591E-1
pbpk_Rb2pS	1.853E-1	1.477E-1	7.767E-2	1.341E-1
pbpk_bw	7.106E-1	7.756E-1	2.671E-1	4.717E-1
pbpk_dose				
pbpk_hemat	7.454E-1	1.007	3.159E-1	5.72E-1
pbpk_kGutabs	2.789E-1	5.104E-1	1.859E-1	2.269E-1
cc3d_Km_AT_APAP	4.354E-1	5.451E-1	1.73E-1	2.375E-1
cc3d_Vmax_AT_APAP	8.539E-1	1.67	4.166E-1	5.628E-1
cc3d_k_AT_APAPG	1.49E-2	4.069E-2	1.453E-2	3.051E-2
cc3d_k_AT_APAPS	2.003E-3	5.633E-3	1.599E-3	2.478E-4
cc3d_k_PD_H2H	3.17E-4	5.192E-4	1.097E-4	4.341E-4
cc3d_k_PD_H2R	5.816E-2	1.085E-1	2.084E-2	4.211E-2
cc3d_k_PD_H2S	2.474E-1	4.192E-1	1.319E-1	1.515E-1
cc3d_k_PD_R2H	3.549E-4	3.853E-4	1.879E-4	9.106E-5
cc3d_k_PD_R2R	7.757E-9	1.391E-8	5.344E-9	3.461E-9
cc3d_k_PD_R2S	5.245E-5	7.406E-5	1.582E-5	3.23E-5
cc3d_k_PD_S2H	5.848E-4	6.227E-4	1.101E-3	2.698E-4
cc3d_k_PD_S2R				
cc3d_k_PD_S2S	1.64E-4	1.947E-4	4.22E-5	8.42E-5
sc_Km_2E1_APAP	9.342E-3	2.078E-2	1.048E-3	7.173E-3
sc_Km_GLUC	5.853E-1	1.03	2.095E-1	3.76E-1
sc_Km_SULF	4.738E-1	5.463E-1	2.074E-1	3.08E-1
sc_Vmax_2E1_APAP	9.508E-3	2.175E-2	9.934E-4	8.017E-3
sc_Vmax_GLUC	5.634E-1	1.14	2.088E-1	3.686E-1
sc_Vmax_SULF	6.567E-1	7.254E-1	2.151E-1	5.296E-1
sc_kGsh	1.347E-9	1.673E-9	1.001E-9	1.369E-9
sc_kNapqiGsh	1.268E-9	2.583E-9	8.232E-10	9.918E-10

[†] See Tables 10 for unit definitions.

8 Human *In Vivo* Reference Data Set

Table 15 lists the values used for calibration and parameter fitting of the complete model. This data is from a study† of nine Caucasians with average BW of 68 Kg following a 20mg/kg (1.4g for a 70 Kg individual) oral dose of APAP syrup. Values are plasma concentrations.

Table 15: Digitized‡ data points from Figure 1 of Critchley *et al.*†

**Time(h)**	**APAP(μg/ml)**	**APAPS(μg/ml)**	**APAPG(μg/ml)**
0	0	0	0
0.3	8.94	0.76	0.33
0.5	14.67	1.97	1.75
0.75	16.77	2.96	3.65
1.0	16.63	3.41	4.71
1.5	14.89	4.06	7.74
2.0	13.98	4.24	9.32
3.0	10.85	4.24	10.78
4.0	8.14	3.71	10.75
5.0	6.12	3.20	9.48
6.0	4.45	2.68	8.13
7.0	3.13	2.15	6.78
8.0	2.72	1.66	5.18
12	1.20	0.72	2.05
24	0.38	0.12	0.27

† Critchley, J. A., Critchley, L. A. H., Anderson, P. J., and Tomlinson, B. “Differences in the single-oral-dose pharmacokinetics and urinary excretion of paracetamol and its conjugates between Hong Kong Chinese and Caucasian subjects.” *Journal of clinical pharmacy and therapeutics*, **30:2** , 179–84, (2005).

‡ Digitized using `http://arohatgi.info/WebPlotDigitizer/`.

9 Simulation Animations

9.1 CC3D Standalone Simulation Animation

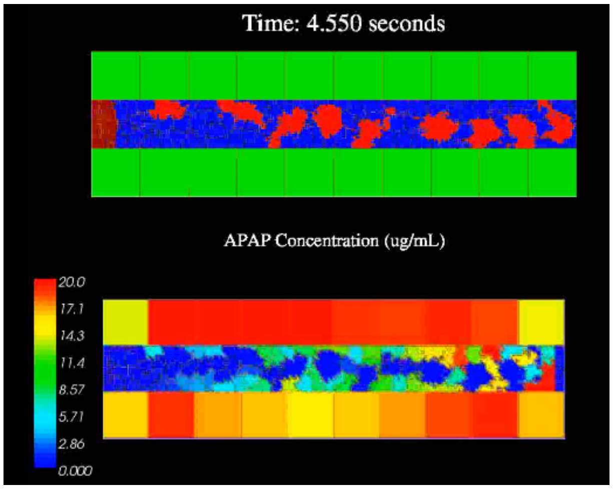

Figure 1: Screen shot of the standalone multi-cell lobule simulation in CompuCell3D. The full animation is in the Supplemental file: APAP_pulse_10sec.avi.

9.2 Complete Eight Hour Simulation Animation

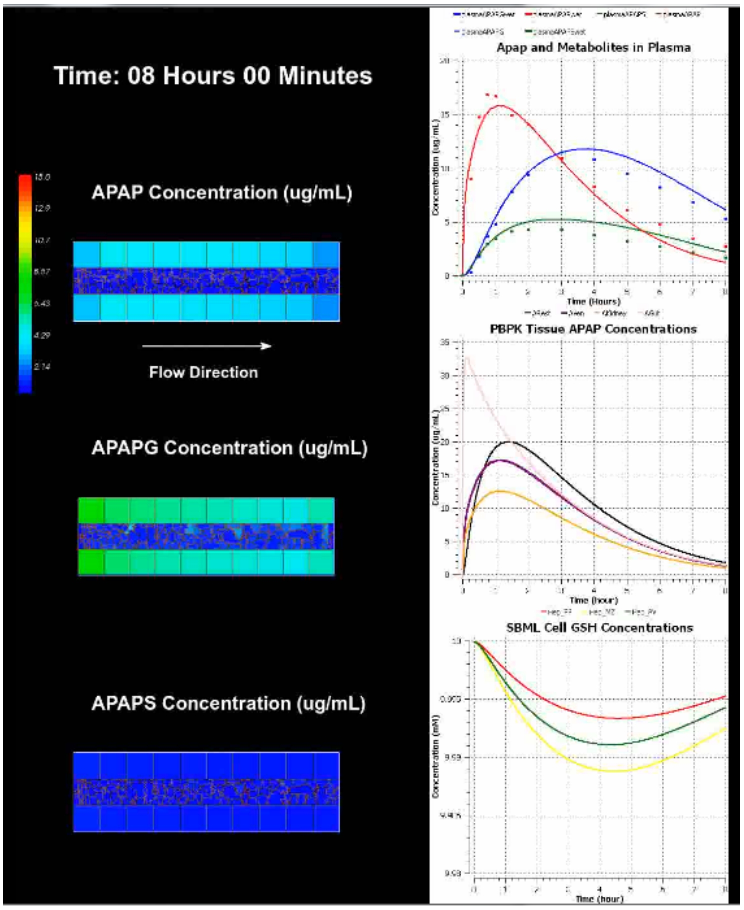

Figure 2: Screen shot of the complete multi-scale eight hour simulation. The full animation is in the Supplemental file: Complete_8hr.avi.
