# Supplementary material for: A Liver-Centric Multiscale Modeling Framework for Xenobiotics
Source: PLoS One. 2016 Sep 16;11(9):e0162428. doi: 10.1371/journal.pone.0162428 (PMC5026379; doi:10.1371/journal.pone.0162428)
Supplement: S3 File — Python code for generating job files for parameter scanning and output analysis. (ZIP) [file pone.0162428.s003.zip › Parameter Scan Script/scanning code routine.docx]

**Lognormal (parameter search)**

Basic equation: $p_{new}={10}^{\alpha}\cdot p_{old}$, where $\alpha$ follows normal distribution $(0,0.25)$. There are two constrains $∁_{1}:-2<\alpha<2; ∁_{2}:-0.1<\alpha<0.1$

1. Parameters not changed are

[pbpk_dose, pbpk_FupG, pbpk_FupS]

1. Parameters obeying $∁_{2}$ are

[pbpk_Rb2p, pbpk_Rb2pG, pbpk_Rb2pS, pbpk_hemat, pbpk_bw, pbpk_Fup]

1. All other parameters obey $∁_{1}$

**PopulationVariability**

Basic equation: $p_{new}=\beta\cdot p_{old}$, where $\alpha$ follows normal distribution $(1,0.25)$. There are two constrains $∁_{1}:0.75<\beta<1.25; ∁_{2}:0.75<\beta<1$

1. Parameters not changed are

[pbpk_dose]

1. Parameters obeying $∁_{2}$ are

[pbpk_FupG, pbpk_FupS]

1. All other parameters obey $∁_{1}$
